# Origin and adaptation to high altitude of Tibetan semi-wild wheat

**DOI:** 10.1038/s41467-020-18738-5

**Published:** 2020-10-08

**Authors:** Weilong Guo, Mingming Xin, Zihao Wang, Yingyin Yao, Zhaorong Hu, Wanjun Song, Kuohai Yu, Yongming Chen, Xiaobo Wang, Panfeng Guan, Rudi Appels, Huiru Peng, Zhongfu Ni, Qixin Sun

**Affiliations:** 1 State Key Laboratory for Agrobiotechnology, Key Laboratory of Crop Heterosis and Utilization (MOE), Beijing Key Laboratory of Crop Genetic Improvement, China Agricultural University, Beijing, 100193 China; 2 AgriBio, Centre for AgriBioscience, Department of Economic Development, Jobs, Transport, and Resources, 5 Ring Road, La Trobe University, Bundoora, VIC 3083 Australia; 3 University of Melbourne, FVAS, Grattan Street, Parkville, VIC 3010 Australia

**Keywords:** Agricultural genetics, Phylogenomics, Natural variation in plants, Plant domestication

## Abstract

Tibetan wheat is grown under environmental constraints at high-altitude conditions, but its underlying adaptation mechanism remains unknown. Here, we present a draft genome sequence of a Tibetan semi-wild wheat (*Triticum aestivum* ssp. *tibetanum* Shao) accession Zang1817 and re-sequence 245 wheat accessions, including world-wide wheat landraces, cultivars as well as Tibetan landraces. We demonstrate that high-altitude environments can trigger extensive reshaping of wheat genomes, and also uncover that Tibetan wheat accessions accumulate high-altitude adapted haplotypes of related genes in response to harsh environmental constraints. Moreover, we find that Tibetan semi-wild wheat is a feral form of Tibetan landrace, and identify two associated loci, including a 0.8-Mb deletion region containing *Brt1/2* homologs and a genomic region with *TaQ-5A* gene, responsible for rachis brittleness during the de-domestication episode. Our study provides confident evidence to support the hypothesis that Tibetan semi-wild wheat is de-domesticated from local landraces, in response to high-altitude extremes.

## Abstract (web summary)

Mechanism of high altitude adaptation of wheat remains unknown. Here, the authors assemble the draft genome of a Tibetan semi-wild wheat accession and resequence 245 wheat accessions to reveal that Tibetan semi-wild wheat has been de-domesticated from local landraces to adapt to high altitude.

## Introduction

**Fig. 1:**
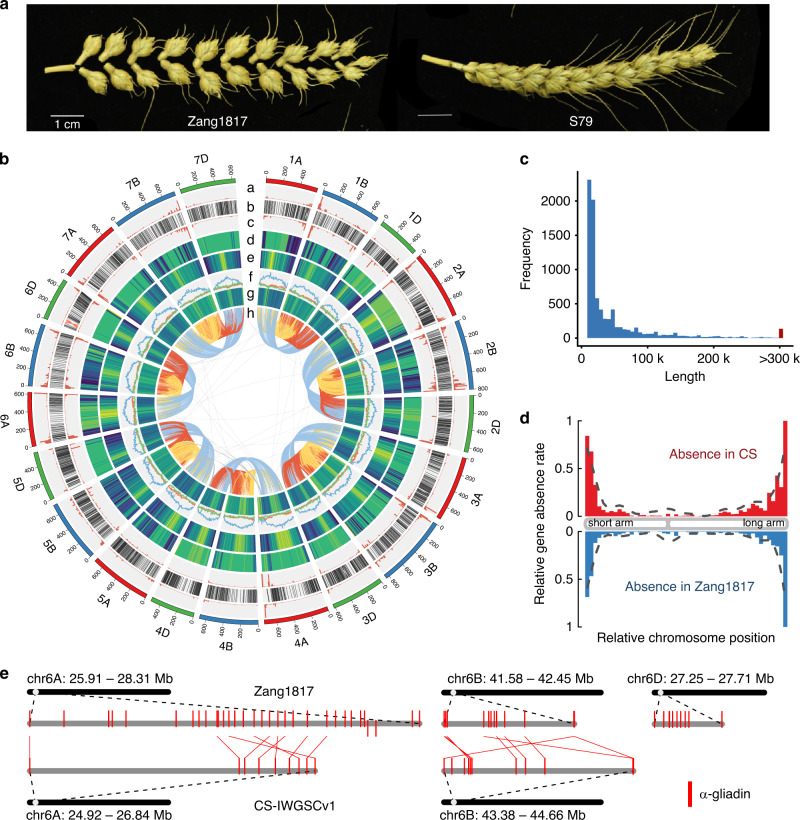
Phenotypes of Tibetan wheat and overview of the Zang1817 assembly. **a** Phenotype of rachis brittleness for Tibetan semi-wild wheat Zang1817 and Tibetan wheat landrace S79. **b** Concentric circle diagram shows distribution of the genomic features of Zang1817: **a** presence–absence variation (PAV) of genes absent in CS; **b** collinear gene pairs between CS and Zang1817 (discordant matching regions are marked in red); **c** PAV genes absent in Zang1817; **d** density of genomic variations (0 to 240,098 bp per 10 Mb); **e** density of high-confidence genes (HC; 0–253 genes per 10 Mb); **f** transposable element (TE) density in 5-kb windows; **g** distribution of K-mer frequency; **h** homeologous gene pairs between subgenomes. **c** Frequency distribution of PAV segment length (>10 kb). **d** Density distribution of the PAV genes across chromosome model. Red, Zang1817-specific retained genes. Blue, CS-specific retained genes. **e** Comparison analysis of genomic regions containing α-gliadin genes on group six homeologous chromosomes. Source data underlying Fig. 1c, d are provided as a Source Data file.

Understanding the evolution and domestication of plants has accelerated crop breeding through being able to target new germplasm for crossing into elite lines and creating a more secure world food supply^1–3^. Hexaploid or bread wheat (*Triticum aestivum*, AABBDD) is a staple food crop for more than one-third of the world population^4^ and originated ~10,000 years ago in the Near-Eastern Fertile Crescent through hybridization between domesticated tetraploid wheat, *Triticum turgidum* (AABB) and wild goatgrass, *Aegilops tauschii* (DD)^5^. As a temperate crop, hexaploid wheat is widely distributed around the world, including the Tibetan Plateau, which has an average altitude of 4268 m above sea level^6^, and is characterized by extensive UV-B irradiation, extremely low temperature and strong hypoxia stress. Although such environmental constraints would be expected to trigger genomic variation of wheat associated with adaptation to high-altitude (HA), the underlying molecular changes are still unknown. Thus, Tibetan wheat provides a significant resource to understand genomic adaptation to the environmental extremes at high altitude conditions. In addition, although wild forms of bread wheat are not found, a unique Tibetan semi-wild hexaploid wheat (*Triticum aestivum* ssp. *tibetanum* Shao) is discovered on the Tibetan Plateau, China^7^. Huang and his colleagues^6^ investigated 17 agronomic traits of 77 Tibetan accessions and 277 Tibetan semi-wild wheat, and found that Tibetan semi-wild wheat phenotypically resembles Tibetan local landraces but differs in brittle rachis characteristics (Fig. 1a). Thus, they suspected that Tibetan semi-wild wheat was a de-domesticated form of Tibetan local landraces as a result of natural selection. Yet, no genetic or genomic evidence is provided to support this hypothesis. Brittle rachis is generally controlled by genes on the short arm of the group 3 chromosomes, especially chr3D^8^, as well as alleles of the *Q* locus on 5A^9^. However, there is a lack of understanding of the genomic variations that contribute to the Tibetan wheat wild-like traits at the population scale.

To address these knowledge gaps, we de novo assemble the genome sequence of a high-altitude wheat accession (Tibetan semi-wild wheat Zang1817), and resequence 245 wheat accessions, including Tibetan semi-wild wheat accessions, Tibetan landraces and cultivars, representing the largest resequencing of world-wide wheat landraces and accessions to date. In combination with previously published 73 re-sequenced accessions^10^, we demonstrate that adaptation to high-altitude environments likely trigger extensive reshaping of the wheat genomes. In particular, we argue that the *TaHY5-like*-mediated signaling pathway might play a role in the adaptation to high-altitude extreme environments in Tibetan wheat and possibly other plants. Furthermore, we uncover a feral origin of Tibetan semi-wild wheat, which is probably, in effect, a de-domesticated form of Tibetan landraces, suggesting that the Tibetan wheat is a resource for fine tuning wheat germplasm to certain environments.

## Results

### De novo assembly of Tibetan semi-wild wheat Zang1817 genome

Genomic variation in HA adapted wheat compared with low-altitude (LA) wheat was determined by generating a de novo genome assembly of Tibetan semi-wild wheat Zang1817. First, we generated >240× coverage sequencing reads from a series of PCR-free Illumina paired-end libraries and mate-paired libraries. The contig and scaffold assembly was performed using the DeNovoMAGIC3 software platform (NRGene, Nes Ziona, Israel), and additional Chromium 10X genomic data were utilized to support scaffold validation and further elongation. Finally, 384,307 scaffolds with N50 of 37.62 Mb were generated, and formed the basis of a total assembly size of 14.71 Gb for Zang1817 (Table 1). The assembled scaffolds were ordered into chromosomes with the aid of the Chinese Spring (CS) IWGSCv1 reference genome^11^. A total of 95.51% scaffolds were ordered to form the 21 high-confidence pseudochromosomes, comprising 1067 scaffolds with 14.05 Gb (Fig. 1b), which is similar to that of the reference CS genome (14.07 Gb)^11^. The Zang1817 genome assembly reported here is a sequenced genome of a semi-wild form of hexaploid wheat to date.

**Table 1:** Summary statistics for the Zang1817 assembly.

	Scaffolds	Contigs
Total scaffolds	384,307	822,893
Assembly size (bp)	14,707,915,341	14,553,951,571
Gaps size (bp)	153,963,770	
Gaps	1.05%	
N50 (bp)	37,620,242	66,264
N50 #sequences	102	63,361
N90 (bp)	5,215,097	13,297
N90 #sequences	499	242,400
MAX (bp)	207,426,720	994,222

A total of 118,078 high-confidence protein-coding genes were inferred from the Zang1817 assembly using protein-homology-based prediction methods supported by RNA-sequencing data. Repetitive elements, a major component of complex genomes, were widely dispersed throughout the wheat genome^11,12^. Up to 82.74% of the Zang1817 assembly sequence were annotated as repeat sequences, including retrotransposons (63.91%), DNA transposons (18.45%) and other unclassified sequence (1.57%). *Copia* and *Gypsy* family retrotransposons accounted for ~44.16% and 15.28% of the Zang1817 assembly sequence, respectively (Supplementary Table 1). The composition of the different classes of repetitive DNA in Zang1817 was highly similar to that in the CS genome. We further evaluated the annotation with 1440 Benchmarking Universal Single Copy Orthologs (BUSCO)^13^ genes, and found 99.51% genes were recalled in the genome, with 94.38~96.81% among the three subgenomes individually, a percentage similar to that of the CS genome (99.72%), thus indicating a near completeness of Zang1817 genome assembly (Supplementary Table 2).

Extensive genomic variations have been reported in maize and rice^14,15^. We found that genomic variation between Zang1817 and CS, when the pseudochromosomes were aligned, mainly occurred in non-coding regions. By comparing the two genomes, we determined the present/absent variation (PAV) for segments longer than 10 kb (Fig. 1c) and identified 345.73 Mb Zang1817-specific genomic segments, including 1875 Zang1817-specific genes compared with CS (Supplementary Data 1). GO analysis revealed that these genes were predominantly enriched in “polysaccharide binding” (GO:0030247) and “serine-type endopeptidase inhibitor activity” (GO:0004867) categories. We also identified 389.29 Mb of genomic segments containing 2540 genes that were absent from the Zang1817 assembly in comparison with CS genome (Supplementary Data 2). These genes were mostly involved in photosynthesis related GO terms including “photosystem II reaction center” (GO:0009539), “chloroplast thylakoid membrane” (GO:0009535), “photosynthesis, light reaction” (GO:0019684) and “cytochrome b6f complex” (GO:0009512). Interestingly, the PAV genes were unevenly distributed across the genome and tended to be distributed at the ends of chromosomes and devoid in centromeric regions (Fig. 1d). A genomic inversion at 366~375 Mb region of chromosome 6D in Zang1817 was revealed based on the genomic comparison, which shared a fine collinearity between Zang1817 assembly and *Aegilops tauschii* genome^12^ (Supplementary Fig. 1), further validating the high-quality of the Zang1817 genome assembly.

The α-gliadin genes confer wheat the unique viscoelastic properties, which facilitate the processing of flour into bread, pasta, noodles, and other food products. An in-depth sequence comparison of the terminal regions of the chromosome group 6 indicated that the duplication and expansion of α-gliadin genes in the three homeologous groups occurred extensively in Zang1817 assembly compared with CS genome. For example, the 2.4 Mb region at 25.91–28.31 Mb on chromosome 6 A from the Zang1817 genome contained 34 α-gliadin genes, more than triple the number from the CS genome (9, region at 24.92–26.84 Mb), and the differences in terms of sequence length between the sequences were mostly caused by differential insertion of repetitive DNA elements. Similar results were revealed for α-gliadin-containing regions at chromosome 6B and 6D (Fig. 1e). On a broader scale, a total of 22,782,409 SNPs were identified in the aligned syntenic blocks between Zang1817 and CS genomes, with 8,085,857, 13,049,811 and 1,646,741 in subgenomes A, B, and D, respectively. Subgenome B had the highest number of SNPs, whereas subgenome D had the lowest number. Although ~99.61% of the Zang1817 sequences matched with CS sequences in syntenic blocks, we were still able to identify 184,913 SNPs that resided in the protein-coding genes, including 95,734/1634 SNPs resulting in amino-acid changes/stop codon occurrences between the Zang1817 and CS genomes, respectively.

### Adaptation mechanism to high-altitude environment

**Fig. 2:**
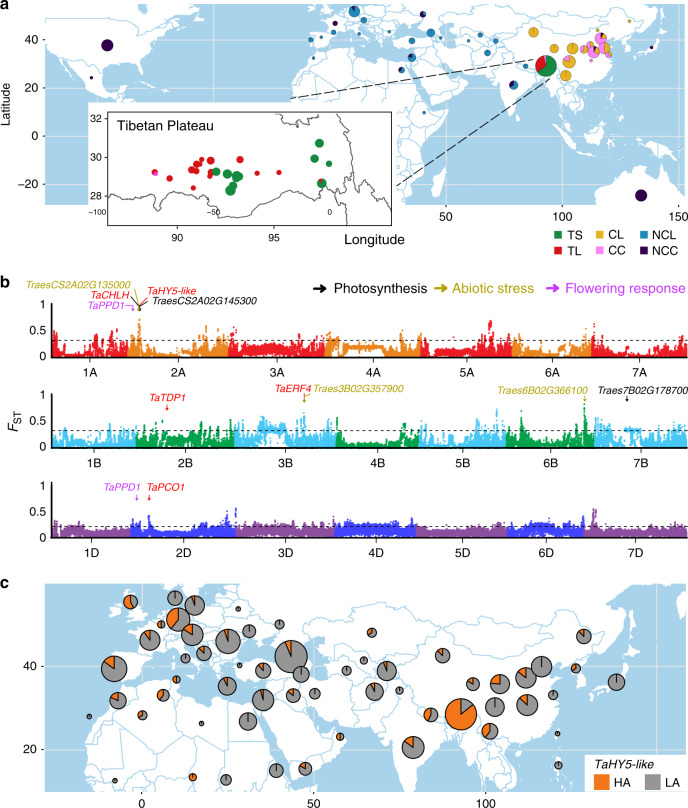
Geographic locations and candidate regions responsive to high-altitude extremes. **a** Global distribution of wheat accessions. NCL, Non-Chinese landrace; NCC, Non-Chinese cultivar; CL, Chinese landrace (excluding Tibetan landrace); CC, Chinese cultivar; TS, Tibetan semi-wild wheat; TL, Tibetan landrace. **b** Genome-wide distribution of genetic differentiation regions between high-altitude (HA) and low-altitude (LA) wheat accessions identified by *F*_ST_ analysis. Each point indicates a 100k-bp window. Dashed lines indicate the top 5% thresholds. Key candidate genes are labeled with arrows. **c** Geographic distribution of different haplotypes of *TaHY5-like* gene. The haplotype distribution analysis was determined using 308 resequencing accessions and 1026 wheat whole-exome capture samples. Orange indicates the proportion of HA adapted haplotype of *TaHY5-like* gene. HA, high-altitude wheat accessions. LA, low-altitude accessions. Geographic maps in **a** and **c** were generated using R packages, including maps, ggmaps, ggplot2, and sf, with geographic information data edited from original version of the Natural Earth project database (version 2013). Source data underlying Fig. 2b are provided as a Source Data file.

High-altitude plants and animals are subjected to genetically adaptive evolution that contributes to their survival in the harsh environment of long exposure to high UV-B irradiation, low temperature and hypoxia. To identify candidate genes and regulatory pathways for plant adaptation to high altitude environments, we re-sequenced 109 Tibetan wheat accessions (including 74 Tibetan semi-wild wheat and 35 Tibetan landraces) as well as 136 representative world-wide wheat landraces and cultivars at a 6.07-fold coverage on average (Fig. 2; Supplementary Data 3). Together with previously published data of 63 hexaploid wheat samples^10^, a total of 46,431,479 filtered SNPs were employed for further analysis. Of these, 0.77% SNPs resided in coding sequences of high-confidence genes, and led to 2813 gains of stop codons (Supplementary Data 4). Phylogenetic analysis revealed that Tibetan semi-wild wheat (69/74) and Tibetan landraces (23/35) grouped together (Supplementary Fig. 2). Interestingly, two landraces originated from Nepal, a high altitude region south of Tibetan Plateau also showed a close relatedness with Tibetan wheat (Supplementary Fig. 2), indicating a potential genetic affinity between each other.

To determine the genomic regions of Tibetan wheat associated with adaptation to the HA environments on Tibetan Plateau, *F*_ST_ for searching genetic differentiation regions was carried out between HA wheat accessions and LA wheat accessions (Fig. 2b; Supplementary Data 5). In total, we identified 1‚905 significant divergent genomic regions including 3847 genes. GO enrichment analysis of those genes showed that functional terms of “serine-type peptidase activity” (GO:0008236) was significantly over-presented. In addition, photosynthesis related GO terms (“chloroplast thylakoid, GO:0009534” and “chlorophyllide a oxygenase [overall] activity, GO:0010277”) and “DNA repair” (GO:0006281) were also enriched (Supplementary Fig. 3). These findings were consistent with previous reports, which indicated that genes involved in stress tolerance and DNA repair are enriched or expanded in high-altitude plants, such as *Eutrema* species^16^, *Crucihimalaya himalaica*^17^, and Maca (*Lepidium meyenii*)^18^.

Significant genomic differentiation was identified on chromosome 2 A, where TraesCS2A02G142800, a homolog of *ELONGATED HYPOCOTYL 5* (*HY5*) in Arabidopsis (Supplementary Fig. 4), was diverged between HA and LA groups featured by two missense variations (designated as *TaHY5-like*, c.1049 G > A|p.Arg350Gln and c.1034 G > T|p.Gly345Val) (Fig. 2b; Supplementary Fig. 5). With the missense SNPs identified between HA (AT haplotype) and LA (GC haplotype) groups in *TaHY5-like*, two major haplotypes were characterized for the 308 resequencing wheat lines together with 1026 whole-exome capture wheat accessions^19^. Significantly, we found that over 86% of Tibetan wheat carry the AT haplotype, compared to wheat from LA environments, which mainly possessed GC haplotypes (Fig. 2c, Supplementary Fig. 5). In addition, we found that wheat orthologues of *TDP1* and *ATG10* in Arabidopsis (TraesCS2B02G244300 and TraesCS2B02G371600) (Supplementary Figs. 6 and 7), which encode a tyrosyl-DNA phosphodiesterase and an E2-conjugating enzyme, respectively, might play important roles in DNA repair in response to UV-B induced DNA damage^20–22^ (Supplementary Figs. 8 and 9), and *TaERF4* (TraesCS3B02G357500) (Supplementary Fig. 10), whose homolog in Arabidopsis is involved in cold acclimation^23^, were also significantly diverged between HA and LA wheat samples (Fig. 2b, Supplementary Fig. 11). Furthermore, TraesCS2A02G134000 (*TaCHLH*) (Supplementary Figs. 12 and 13), a homolog of *CHLH* involving in light-dependent chlorophyll accumulation^24^, together with other photosynthesis related genes including TraesCS2A01G145300 (Supplementary Fig. 14) and TraesCS7B02G178700 (Supplementary Fig. 15), also showed haplotype divergence between HA and LA wheat groups (Fig. 2b; Supplementary Fig. 16). Interestingly, the downstream genes *ERF4* and *CHLH* are known to be positively regulated by HY5 in Arabidopsis, the promoter regions of *TaERF4*, *TaPCO1*, *TaCHLH,* and *TaTDP1* also contained HY5 regulating cis-element (CACGTG), indicating that they are potentially regulated by TaHY5-like in wheat. The HY5 is a master regulator extensively involved in regulating light signaling, DNA repair, cold acclimation, chlorophyll synthesis and hypoxia response in plants^25^. Given that light intensities, low temperature, and hypoxia frequently occur simultaneously on Tibetan Plateau, it is likely that the signaling modules have co-evolved to cope with these stimuli. We found that the *TaHY5-like* hub as well as *TaHY5-like*-mediated pathway of Tibetan wheat were the targets of positive selection, leading to their adaptation to the extreme. Interestingly, the geographic distribution analysis of haplotype diversity of wheat homologs of *TaHY5-like, TaERF4*, *TaPCO1*, *TaCHLH,* and *TaTDP1* showed that the combination of five high-altitude specific haplotypes mainly exhibited in Tibetan plateau (Fig. 2c, Supplementary Fig. 17). These observations suggest that a rapid evolution of *TaHY5-like*-mediated functional pathway occurred in Tibetan wheat in response to the harsh constraints in HA environments (Supplementary Fig. 18). To support our hypothesis, we analyzed the haplotypes of Nepal wheat lines, which is also planted at high altitude (~1200–4800 meters high), and these HA adapted wheat accessions possessed the same haplotype to Tibetan wheat, including *TaHY5-like, TaERF4, TaCHLH, TaPCO1, TaTDP1*. To confirm the hypothesis, further evidence is needed to functionally validate their biological significance in adaptation to high altitude.

Regulation of flowering time also contributes to the environmental adaptation in wheat^26^, and Tibetan semi-wild wheat accessions exhibited delayed heading date compared with other wheat species sowed with the same sowing date^7^. *PPD1* gene is a major determining factor affecting the photoperiod response of wheat^27^ and barley^28^. Consistent with this observation, we identified related genes in HA wheat that may have been targeted by natural selection during the adaptation process. The *TaPPD1-2A* (TraesCS2A02G081900) (Supplementary Fig. 19) and *TaPPD1-2D* (TraesCS2D02G079600) (Supplementary Fig. 19) genes, two homeologs underlying major quantitative trait locus for photoperiod-dependent flowering^29^, were dramatically diversified between HA and LA wheat accessions based on *F*_ST_ analysis (Supplementary Figs. 20 and 21), suggesting strong selection on this locus in high-altitude wheat populations. A comparison of the haplotypes between LA and HA wheat groups at these two genes identified two differentiated SNPs for *TaPPD1-2A* (c.1202 A > G|p.Asp401Gly) and *TaPPD1-2D* (c.1018 C > T|p.Arg340*), respectively. These two candidate causal mutations were fixed in HA wheat, which together accounted for 87.61%, and has low allele frequency (33.33%) in LA population. The HA-haplotype combination of *TaPPD1-2A* and *TaPPD1-2D* only occurred in Tibetan Plateau occupying 51% of the whole HA group at Tibetan Plateau (Supplementary Fig. 22). This observation suggested that Tibetan wheat have been selected to achieve appropriate flowering time to complete their life cycles in Tibet.

### Origin of Tibetan semi-wild wheat

**Fig. 3:**
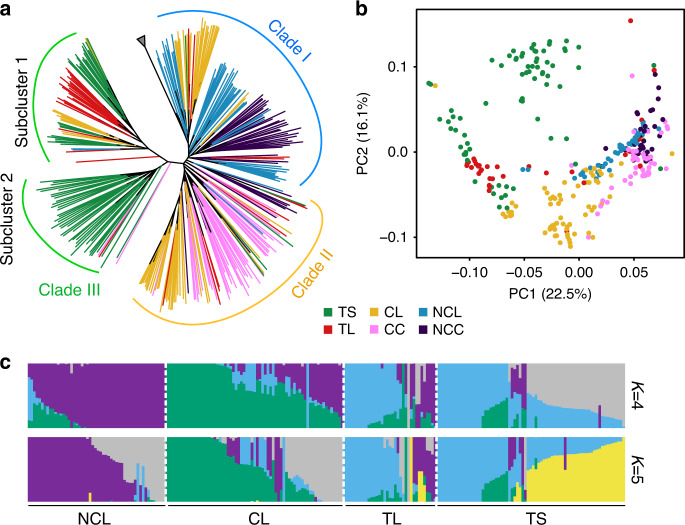
Population structure analysis of resequencing wheat accessions. **a** A neighbor joining (NJ) phylogenetic tree of wheat resequencing samples using 364,856 high-confidence SNPs on subgenome D. Gray triangle, the outgroup, *Aegilops tauschii*, which is wild diploid progenitor for D genome in wheat. **b** PCA plot of resequencing samples based on SNPs in subgenome D. **c** Individual ancestry coefficients of resequencing accessions inferred using ADMIXTURE using SNPs in subgenome D. Each color represents one population. The length of each segment in each vertical bar represents the proportion contributed by ancestral populations. Source data are provided as a Source Data file.

Tibetan semi-wild wheat is a unique form of hexaploid wheat^30^. To provide insights into their evolutionary origin, we performed a comprehensive population structure analyses of available accessions based on 364,856 high-confidence homologous SNPs on subgenome D using *Aegilops tauschii* accessions as an outgroup. This use of the D subgenome was necessitated based on previous analyses that extensive wild-relative introgressions were observed in the subgenome A and B of hexaploid wheat^19^, rendered the analysis using these subgenomes together to be too complex and ambiguous. The resulting phylogenetic tree clearly encompassed three major clades, among which, a portion of non-Tibetan Chinese landraces (CL, 26/67) together with almost all of non-Chinese landraces (NCL, 49/52) and non-Chinese cultivars (NCC, 35/38) formed Clade I, indicating a potential contribution of non-Chinese wheat to the genetic diversity of Chinese wheat (Fig. 3a). In contrast, most of the remaining non-Tibetan Chinese landraces (32/41) and almost all of the elite cultivars (CC, 36/42) clustered into Clade II, suggesting that Chinese landraces contributed significantly to varietal replacement during breeding process in China. Tibetan semi-wild wheat and Tibetan landraces were grouped into Clade III, which was further separated into two distinct subclusters in the phylogenetic tree, that is, a total of 41 (55.41%) Tibetan semi-wild wheat accessions were grouped into a monophyletic group (Subcluster I), whereas 28 (37.84%) Tibetan semi-wild wheat accessions together with 63% Tibetan landraces (TL, 22/35) formed a second subcluster (Subcluster II), revealing that the Tibetan semi-wild accessions shared a closer genetic relationship with Tibetan landraces compared with the other wheat samples (Fig. 3a). We also performed phylogenetic analysis of wheat accessions using SNPs in the whole-genome and A&B subgenomes, respectively, and revealed consistent observations (Supplementary Fig. 23).

**Fig. 4:**
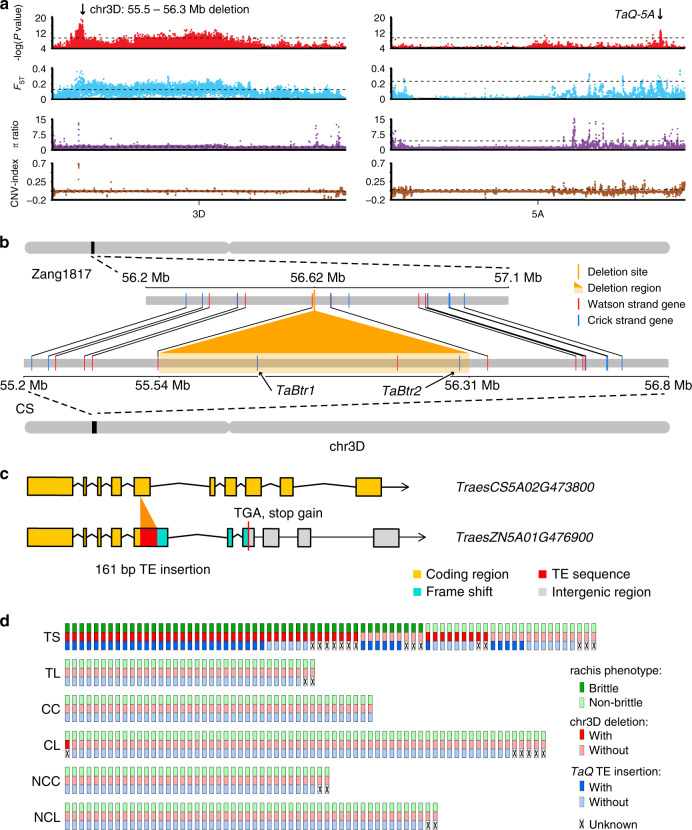
Genomic differentiation during de-domestication process. **a** Genome-wide scanning of candidate regions for rachis brittleness during wheat de-domestication process using GWAS analysis. Dashed lines represent the top 5% thresholds. Two significant divergent regions (0.8-Mb genomic region in chr3D and *TaQ-5A*) are labeled above the dot peaks. **b** Genomic structure comparison of 0.8-Mb genomic region with significant divergence between the Zang1817 assembly (upper panel) and CS reference genome (lower panel). *BRITTLE RACHIS*-LIKE gene potentially related to rachis brittleness locates in the region. Collinearity of high-confidence genes between two genomes were shown in lines. Dashed line indicates un-annotated genes although with intact genomic sequence. **c** Genomic comparison of *TaQ-5A* between Zang1817 and CS genomes. A 161 bp TE insertion (red) was identified in Zang1817, resulting in frame-shift variation and gain of stop codon (red line). **d** Schematic diagram of genomic variations and phenotype alterations related to wheat rachis brittleness. Source data underlying Fig. 4a are provided as a Source Data file.

Principal component analysis (PCA) also showed a close affinity of Tibetan semi-wild wheat with Tibetan landraces (Fig. 3b; Supplementary Fig. 24). This inferred relationship was supported by a maximum likelihood estimation of individual ancestries performed using ADMIXTURE where Tibetan semi-wild wheat accessions shared a greater proportion of genetic composition with Tibetan landrace group than with other wheat samples according to the ancestry states with K from 4 to 5 (Fig. 3c; Supplementary Fig. 25). Taken together, we suggest that Tibetan semi-wild wheat was a de-domesticated form of Tibetan wheat landraces. Furthermore, genome-wide nucleotide diversity was lower in the Tibetan semi-wild wheat population (*π* = 5.38 × 10^−4^) than in landrace-counterparts (*π* = 5.67 × 10^−4^), indicating a limited genetic background of Tibetan semi-wild wheat available during the adaptation process in the Tibetan Plateau. To further confirm our hypothesis, we tested the origin models of Tibetan semi-wild wheat on eight different demographic simulation models (Supplementary Table 3; Supplementary Fig. 26) and found that the model with bottleneck and without any migration (Supplementary Fig. 26) fit the genetic data best (Supplementary Table 3), indicating that initial Tibetan semi-wild wheat accessions are derived from Tibetan landraces by de-domestication followed by a bottleneck period resulting from developed agriculture activities. Our genome-wide association study (GWAS) identified two loci of particular interest as being significantly associated with the rachis brittleness phenotype in Tibetan semi-wide wheat, including a 0.8-Mb deletion region on 3D and a genetic locus containing *TaQ-5A* gene on 5 A (see Fig. 4a, Supplementary Fig. 27–30). The association was also supported by results from *F*_ST_, Pi-ratio, and CNV-index analysis. Approximately 20.51% of the rachis brittleness variation could be explained by a 0.8-Mb deletion region (55.5–56.3 Mb) on chromosome 3D, as identified from the GWAS analysis of the entire population (Fig. 4a). Further investigation showed that the deleted genomic region contained two *BRITTLE RACHIS*-LIKE genes (TraesCS3D02G103200 and TraesCS3D02G103400) (Supplementary Fig. 31), homologs of the *Btr1* and *Btr2* genes controlling rachis brittleness^31^. This finding suggested that the identified locus was a significant contributor to rachis brittleness in Tibetan semi-wild wheat. Consistently, a previous study revealed that brittle rachis of Tibetan semi-wide wheat is governed by a locus on the short arm of chromosome 3D^32^. Further investigation showed that all the homeologs of *Btr1* and *Btr2* gene in subgenome A and B of Zang1817 shared almost the same amino-acid sequence with the respective standards in CS (Supplementary Fig. 32), and genomic variations only occurred as the *Btr1/2* homologs in subgenome D were lost in Zang1817 assembly (Supplementary Fig. 33). However, *Btr1* and *Btr2* gene mutations lead to a non-brittle rachis phenotype in barley according to the previous report^31^, thus the deletion in their homologs of wheat cannot directly explain the brittleness variation. Therefore, the data suggest that there might be other gene interactions controlling rachis brittleness in semi-wild wheat associated with the 0.8-Mb deletion; it requires further biological investigation to develop this issue further. The locus at chromosome 5 A including the *TaQ-5A* gene, is a well-established contributing factor for brittle rachis in wheat^33,34^. GWAS and *F*_ST_ analysis narrowed the candidate region down to 0.1 Mb (chr5A:650.1–650.2 Mb), although several other SNPs located in non-coding region were also found to be associated with the phenotypic variation. In addition, the 161-bp TE insertion has been functionally validated to be contributing to rachis brittleness previously^9^. Therefore, we propose that the 161-bp insertion in the fifth exon of *TaQ-5A* gene (TraesCS5A02G473800) on chromosome 5A^34^ is also a major factor affecting rachis brittleness, accounting for ∼28.96% of the phenotypic variation in the entire population (Fig. 4a, Supplementary Fig. 27). The 0.8-Mb genomic sequence deletion and the 161-bp insertion in *TaQ-5A* gene were evident in Zang1817 genome in the comparison to CS (Fig. 4b, c). However, we also found several wheat accessions with either the deletion or the insertion genomic variations exhibiting rachis non-brittleness (Fig. 4d), indicating that there might be other genomic loci contributing to this phenotypic variation. Yet, this study provides targets for the further investigation of the gene network contributing to rachis brittleness. We conclude that Tibetan semi-wild wheat has undergone a de-domestication-like process during its evolution history with a selective footprint related to rachis brittleness, representing a feral form of Tibetan local wheat. Crop ferality is observed in many crop species, including rice and barley^35–38^ and this report provides a genomic evidence for a de-domestication event in Triticeae species.

## Discussion

It is estimated that wheat was introduced into China ~5000 years ago, and planted in Tibet as early as ~3500 years ago^39^. Here, we report that the colonization of wheat in the Tibetan Plateau appears to have co-selected primitive sequence diversity and post-fixation of haplotypes for adaptation in high-altitude environments including, high light intensities, low temperature and hypoxia stresses in addition to photoperiod response. We propose a *TaHY5-*like regulation hub, featuring two main haplotypes in HA and LA wheat populations, has been advantageous to Tibetan wheat adaptation to HA extremes. In addition, the downstream genes of *TaHY5-like*, including *TaERF4*, *TaPCO1*, *TaCHLH,* and *TaTDP1* involved in cold acclimation, hypoxia adaptation, and high light intensity and UV radiation response, respectively, also showed sequence differentiation in terms of altitude. During the long history of the HA adaptation process, we propose that Tibetan semi-wild wheat has undergone a de-domestication event linked to a genomic footprint including a 0.8-Mb deletion region on 3D containing *Btr1/2* homologs and a genetic loci containing *TaQ-5A* gene on 5A, that correlates with rachis brittleness can be considered a feral form of Tibetan local landrace. Feral forms are observed in many crop species, including rice and barley^37,38^ and this report provides evidence for defining new targets that can delineate the gene network underpinning the de-domestication event in *Triticum* species.

## Methods

### DNA extraction and sequencing

Genomic DNAs were isolated from the leaves of semi-wild wheat Zang1817 (*Triticum aestivum* ssp. *tibetanum* Shao) using the modified CTAB^40^ and subjected to libraries construction. Two PCR-free paired-end shotgun libraries were constructed using DNA template size of ~470 bp, and sequenced on the Hiseq2500 v2 Rapid mode as 2 × 265 bp reads. PCR-free genomic library of ~800 bp DNA template size was prepared using the TruSeq DNA Sample Preparation Kit version 2 according to the manufacturer’s protocol (Illumina, San Diego, CA), and sequenced on an Illumina HiSeq2500 as 2 × 160 bp reads (using the v4 illumina chemistry). Ten genomic DNA libraries ranging from 470 bp to 10 K bp were further constructed and sequenced. Six Mate-Pair (MP) libraries with jump ranges of 2–5 Kb, 5–7 Kb, and 7–10 Kb were prepared utilizing the Illumina Nextera Mate-Pair Sample Preparation Kit (Illumina, San Diego, CA), and sequenced on HiSeq4000 as 2 × 150 bp reads. PE and MP libraries construction and sequencing were conducted at Roy J. Carver Biotechnology Center, University of Illinois at Urbana-Champaign. The 10X genomics library was constructed with DNA fragment longer than 50 Kb and was sequenced with the Gemcode platform (10X genomics, Pleasanton, CA). 10X Chromium library construction and sequencing were conducted at HudsonAlpha Institute for Biotechnology, Huntsville, Alabama. A total of ~3,535 Gb Illumina sequencing data was produced for semi-wild wheat Zang1817 together with ~680 Gb of 10X Chromium library sequencing data.

### Scaffold assembly

Genome assembly was constructed from short sequencing data by the proprietary software package DenovoMAGIC-3.0 (NRgene)^41^. Main steps to construct the Zang1817 assembly scaffolds include: (1) reads pre-processing: removing Illumina adaptors, PCR duplicates and Nextera linkers. Create the stitched reads by merging overlapping reads in PE libraries with minimal overlap of 10 bp. Then scan and filter the putative sequencing errors in stitched reads; (2) contigs assembly: building the De Bruijn graph (k-mer length = 127 or 191 bp) of contigs from stitched reads. For repeat resolving and contigs extension, reliable paths in the graph between contigs were found using PE reads; (3) scaffolds assembly: contigs were linked into scaffolds and gaps in between were estimated with PE and MP information. Then unique path connecting the gap edges was detected utilizing PE and MP links and De Bruijn graph information; (4) scaffolds validation and extension: 10X barcoded reads were mapped to the assembled scaffolds, and clusters of reads were identified to be part of a single molecule when they share same barcode that mapped to adjacent contigs in the scaffolds. Each scaffold was further scanned with a 20 kb window, and the number of distinct clusters covering the entire window was ensured to be statistically significant regarding the number of clusters that span the entire window. A scaffolds graph linking two scaffolds with more than two common barcodes is then generated by comparing the barcodes mapped to the scaffold edges. Linear scaffolds paths in the scaffolds graph formed the final scaffolds.

### Construction of pseudomolecules

The scaffolds of Zang1817 were anchored to chromosomes with the reference of CS-IWGSCv1 assembly. Simulated reads of both Zang1817 scaffolds reference genome and CS-IWGSCv1 reference genome were generated as paired-end short sequences, in length of 500 bp with insertion size of 500 bp and with step size of 1000 bp. The reads were aligned to each other reference genome mutually utilizing the BWA-mem aligner v0.7.15^42^. Each of the Zang1817 scaffolds was first grouped to a specific chromosome, by which chromosome were aligned with most simulated reads from this scaffold. Scaffolds with <22 simulated reads aligned to one chromosome were reserved to build the pseudo-sequence notated as chrUn. For each of the scaffolds grouped in one chromosome, the first five continuously aligned reads and last five continuously aligned reads were identified and then used for deciding the relative-position and the direction. Then the scaffolds grouped in each chromosome were ordered by the related-positions and directions and then were concatenating into a pseudo-chromosome sequence, with insertion gaps of 300 bp. Finally, the 21 pseudochromosomes and a chrUn were generated for the Zang1817 assembly.

### Gene prediction and annotation

To aid genome annotation and perform the transcriptome analysis, we generated the RNA-seq data for four different tissues, including leaf, root, stem, and spike. All fresh tissues were frozen in liquid nitrogen, and stored at −80 °C before processing. Paired-end RNA libraries were constructed using the Illumina RNA-Seq kit (Illumina Inc. San Diego, CA, USA) using pooled RNA, which were sequenced using the pair-ends sequencing module (2 × 150 bp) on HiSeq X Ten platform. In total, sequencing resulted in 465.91 million reads. Reads contaminated with adapters were detected and removed using Trimmomatic v0.36^43^. Poor-quality reads (Phred score <20) were trimmed from both ends with SolexaQA v2.0^44^; Only the reads with lengths ≥25 bp on both sides of the paired-end format were subjected to further analysis.

Protein-coding genes were annotated using a comprehensive strategy by combining results obtained from homology-based prediction, ab initio prediction, and RNA-seq-based prediction methods. Annotated gene protein sequences from *Triticum turgidum*, *Triticum aestivum*, *Triticum urartu*, *Aegilops tauschii*, *Hordeum vulgare*, *Brachypodium distachyon*, *Oryza sativa,* and *Zea mays* were aligned to the *Triticum aestivum* genome assembly using WUblast v2.0 with an *E* value cutoff of 10^−5^ and the hits were conjoined by Solar software. GeneWise v2.4.1^45^ was used to predict the exact gene structure of the corresponding genomic regions for each WUblast hit. Gene structure created by GeneWise was denoted as Homo-set (homology-based prediction gene set). RNA-seq reads were assembly by Trinity v2.4^46^ software; the assembly results were directly mapped to the *Triticum aestivum* genome assembly by BLAT v36^47^ and assembled by PASA v2.1.0^48^. Gene models created by PASA were denoted as the PASA-Iso-set (PASA-Iso-Seq set), and the training data for the ab initio gene prediction programs. Five ab initio gene prediction programs (Augustus v2.5.5^49^, Genscan v1.0^50^, Geneid v1.4^51^, GlimmerHMM v3.0.4^52^, and SNAP v2006-07-28^53^) were used to predict coding regions in the repeat-masked genome. In addition, Illumina RNA-seq data were mapped to the assembly using Tophat v2.0.13^54^, and Cufflinks v2.2.1^55^ was then used to assemble the transcripts into gene models (Cufflinks-set). Gene model evidence from the Homo-set, PASA, Cufflinks-set, ab initio programs were combined by EvidenceModeler v1.1.1^56^ into a non-redundant set of gene annotations. Weights for each type of evidence were set as follows: PASA>Homo-set>Cufflinks-set>Augustus>GeneID=SNAP=GlimmerHMM=Genscan. Gene models were filtered out by these criteria: (1) coding region lengths of 150 bp, (2) supported only by ab initio methods and with FPKM < 1.

All protein-coding genes were aligned to two integrated protein sequence databases: SwissProt (release 2015_08) and NR (release 2015_08). Protein domains were annotated by searching against the InterPro v32 using InterProScan v5^57^ and Pfam v27.0 databases by HMMER v3.1^58^, respectively. The Gene Ontology (GO) terms for each gene were obtained from the corresponding InterPro or Pfam entry. The pathways in which the genes might be involved were assigned by BLAST against the KEGG databases (release 53), with an *E* value cutoff of 10^−5^. Functional annotation results were finally combined from two strategies above.

### Annotation of repetitive DNA

The transposable elements (TE) were annotated through homology-based prediction method. Two TE libraries containing 3050 complete wheat TEs sequences (ClariTeRep: https://github.com/jdaron/CLARI-TE) and 2825 complete plant TE sequences (http://botserv2.uzh.ch/kelldata/trep-db/downloads/trep-db_complete_Rel-16.fasta.gz) were downloaded and combined. RepeatMasker vopen-4.0.5^59^ was used to annotate TEs in *Triticum aestivum* genomes using the combined data sets as searching library.

### Assembly and annotation evaluation

We evaluated the assembly with 1440 Benchmarking Universal Single Copy Orthologs (BUSCOv3) genes from plants set^13^. The whole genome was evaluated together, and meanwhile, each subgenome (A, B, D) was also evaluated individually. To evaluate the quality of the Zang1817 assembly, we first inspected the genome-wide 1,888,547,646 (~20× expected coverage) random selected raw reads. To be compatible with the whole-genome sequencing of CS technically, 150 bp PE sequencing data were generated from the 450 bp PE library. These reads were aligned to Zang1817 assembly using BWA-mem with default parameters, resulting in 1,886,201,978 (99.88%) mapped reads.

### Dot plot for comparing the assemblies

Simulated reads (500 bp PE) of both assemblies were used to be aligned to each other assembly with BWA-mem. Using the aligned position and oriented position of each simulated read, we generated the dot plot showing the collinearity between these two genomes across the pseudochromosomes of each assembly. Beyond the comparison between Zang1817 and CS-IWGSCv1 assemblies, we further compared Zang1817 and *Aegilops tauschii* Aet v4.0 assemblies^12^ in the same way.

### Collinear analysis of α-gliadin

The gliadins genes are amplified in number in wheat, and the gliadin family is important for studying the wheat grain protein. To reveal the genomic difference in gene level, we selected the α-gliadin gene family to construct the collinearity between Zang1817 and CS-IWGSCv1 assemblies. We used the α-gliadin gene sequences from CS as queries to identify all hits in assemblies of CS and Zang1817 using BLAST v2.6.0^60^ with *E* value ≤ 10^−5^, similarity ≥75% and identity ≥75%. The bedtools v2.27.1^61^ were used to annotate all hits with genome annotation (high confidence and low confidence) and generate corresponding sequences. To reveal the collinearity, we used the reciprocal best hits for all these α-gliadin genes from BLAST results, and the gene pairs with multiple best hits in different chromosomes were discarded.

### Identify homologous cluster for collinear analysis

Homologous gene cluster analyzed between Zang1817 and CS were characterized using OrthoMCL v2.0.9^62^. The transcript with the longest protein sequence of each gene was used to represent the gene. The default setting *orthomclFilterFasta* module was used to filter out poor-quality proteins. Mutual similarities were calculated by performing BLASTP. Then Markov clustering was performed based on the similarities in order to define gene groups with default inflation value. Thus, 12,915 homologous gene clusters with at least two members were identified between Zang1817 and CS-IWGSCv1. Finally, the homologous gene clusters were used for collinear analysis.

### Syntenic blocks detection

Syntenic blocks between CS-IWGSCv1 and Zang1817 assemblies were detected by MCScanX v1.0^63^ and further merged by Quota Align v1.0^64^ using high confidence genes as anchors. The longest CoDing Sequence (CDS) was used as the representative sequence of each gene for CS-IWGSCv1. First, pairwise sequence similarities between all CDS sequences of CS-IWGSCv1 and Zang1817 were calculated using BLASTN with an *E* value cutoff of 10^−10^. Then syntenic blocks were detected using MCScanX with default parameters. Quota Align was further used to merge adjacent syntenic blocks. The syntenic depth was set to 1:1. The overlap cutoff was set to 40, and the distance cutoff was set to 20.

### Identification of PAV regions and genes

PAV segments between CS-IWGSCv1 and Zang1817 were defined through a read depth based method. To identify Zang1817-specific regions, we aligned raw resequencing reads of Zang1817 and CS to Zang1817 and CS-IWGSCv1 reference genomes using BWA-mem with default parameters. Then the reads depths were calculated by windows (window size, 5K-bp) crossing Zang1817 reference genome, and then were normalized by the whole-genome average coverages. The windows that could be normally aligned by Zang1817 itself (normalized read depth >0.8) but very low covered by CS resequencing data (normalized read depth <0.2) were identified as Zang1817-retained sequences. The identified windows with adjacent distances <10k-bp were merged together. The finally merged windows were defined as Zang1817-retained regions. CS PAV regions were identified in the same way. Finally, 9385 windows with total length of 345.73 M bp were identified as Zang1817-specific retained regions; and 8265 windows with total length of 389.30 M bp were identified as CS-specific retained regions.

PAV genes were defined in a similar manner but in level of genes. To identify Zang1817-specific retained gene, average read depths were calculated for each annotated gene and normalized by the whole-genome average read depth. Genes that could be covered normally by Zang1817 (normalized read depth >0.8) but low covered by CS resequencing data (normalized read depth <0.2) were classified as Zang1817-specific retained genes. CS-retained genes were identified in the same way.

The MUMmerv4 module NUCmer^65^ was used to detect large structural variations between CS-IWGSCv1 and Zang1817 assemblies. Each chromosome of Zang1817 was aligned to the corresponding chromosome of CS using NUCmer with default parameters. The alignments were further filtered using delta-filter with parameter “−1” to retain only 1-to-1 alignment. The filtered alignments were visualized as dot plot using R.

### Identification of SNPs and INDELs between assemblies

To identify SNPs and INDELs between the CS-IWGSCv1 and Zang1817 assemblies, we first used NUCmer for alignment with the parameters “-c 90 -l 40 -t 4 –mum”. The delta file was further filtered using delta-filter (module of MUMmer) to obtain 1-to-1 alignment block. Then show-snps (module of MUMmer) with parameters “-ClrTH” was used to identify SNPs and INDELs.

### Sampling and sequencing

The re-sequenced collection contains 308 hexaploid wheat accessions in total, covering 74 Tibetan semi-wild accessions, 43 Chinese cultivars accessions and 102 Chinese landrace accessions (including 35 Tibetan landrace accessions), and 89 accessions from countries word-widely beyond China. The re-sequenced 245 wheat accessions in this study and previous published 73 accessions^10^ were combined together in this study. Tibetan semi-wild accessions and chinese landrace accessions were obtained from National center for crop germplasm preservation and Sichuan Agricultural University. Genomic DNA was extracted from young leaves following a standard CTAB protocol. DNA libraries were constructed by Novogene and sequenced with the Illumina Hiseq Xten PE150 platform with insert size of approximately 500 bp.

### Collection of published wheat resequencing data

A panel of previous published resequencing data was used^10^; and the raw genotyping data in GVCF formats of the hexaploid wheats were requested from the authors. To reveal the geographical distribution of haplotypes for important adaptive genes in a large genetic background, we merged the resequencing data in this study with the recently published whole-exome capture (WEC) data set for 1026 hexaploid wheat accessions. The WEC data set was downloaded from http://wheatgenomics.plantpath.ksu.edu/1000EC/, covering ~8.87 million SNP sites.

### Alignment and genomic variation calling

Raw reads were trimmed using Trimmomatic, and high-quality clean reads were mapped to the CS wheat reference genome (IWGSC RefSeq v1.0)^11^ using BWA-MEM. Read pairs with abnormal insert sizes (>10,000 bp or <−10,000 bp or =0 bp) or low mapping qualities (<1) were filtered using Bamtools v2.4.1^66^. Potential PCR duplicates reads were further removed using Samtools v1.3.1^67^. SNPs and INDELs were identified through all 245 samples by the HaplotypeCaller module of GATK v3.8^68^ in GVCF mode. Then joint call was performed using GATK GenotypeGVCFs for all 318 samples. SNPs were preliminarily filtered using GATK VariantFiltration function with the parameter “–filterExpression QD < 2.0| | FS > 60.0| | MQRankSum < −12.5| | ReadPosRankSum < −8.0| | SOR > 3.0| | MQ < 40.0| | DP > 30| | DP < 3.” The filtering settings for INDELs were “QD < 2.0, FS > 200.0,” and “ReadPosRankSum < −20.0| | DP > 30 | | DP < 3”. SNPs and INDELs that did not meet any of the following criterias were further discarded: (1) MAF ≥ 5% (2) Missing rate ≤ 40% (3) bi-allelic sites. Finally, the identified SNPs and INDELs were further annotated with SnpEff v4.3^69^.

### Population genetics analysis

We assessed the overall population genetic structure of 308 hexaploid accessions using neighbor joining (NJ) phylogenetic tree, PCA, and ADMIXTURE v1.3.0^70^ for non-hierarchical, quantitative clustering analyses. For all these analyses, we used a non-redundant SNP data set obtained after removing rare alleles with minor allele frequency <5% and genotype missing ratio >10%, and further pruning out SNPs with intra-chromosomal LD *r*^2^ < 0.4 to remove the bias caused by LD.

### Phylogenetic analysis

The NJ phylogenetic tree was obtained by calculating the pairwise genetic distances using PLINK v1.9^71^ with parameters “–distance square 1-ibs”. The tree was constructed using njs method in the R package ape v5.3^72^. NJ-tree of A&B subgenome was rooted with five wild emmer accessions as outgroup^10^. NJ-tree of D subgenome was rooted with five *Aegilops tauschii* accessions as outgroup^10^.

### Population structure analysis

The population structure analysis was performed using ADMIXTURE, with *K* values from 2 to 6 with 10 replications using different random seeds. The replicate with the highest log-likelihood for each *K* was used for representing population structure.

### PCA analysis

PCA analysis was performed using smartpca program embedded in EIGENSOFT v6.1.4^73^ with default settings.

### Demographic analysis

Fourfold degenerate SNPs on D subgenome of Tibetan semi-wild wheat (TS) and Tibetan landraces (TL) in subcluster I of Fig. 3a were used to minimize bias due to sample population structure. To reduce the possibility of missing data, TL and TS were downsampled to 30 and 40 via projection, respectively. Two-dimension site frequency spectrum was generated using easySFS (https://github.com/isaacovercast/easySFS). Eight demographic models were constructed and fitted by 1000 independent runs with randomized starting points utilizing ∂a∂i v2.0.5^74^ (Supplementary Fig. 26). Given the observed site frequency spectrum, the parameters of each model with highest log-likelihood were selected as best fitting parameters. We scaled population size by *N*_*e*_ (ancestral effective population size) to estimate the population size in number of individuals, and scaled migration rate and time by 2*N*_*e*_ to estimate number of migration per generation and time in years. The ancestral population size was estimated by the formula *θ* = 4 × *N*_e_ × *μ* × *L*, where the neutral mutation rate (*μ*) is set to be 6.5 × 10^−9^ according to Molina et al.^75^, and the generation time (*L*) was assumed to be 1 year. *θ* was computed based on site frequency spectrum of D subgenome using ∂a∂i. Therefore, *N*_*e*_ was estimated as 67,003.

### Rachis brittleness phenotyping

Field trials were conducted for 1 year at China Agricultural University research station located at Beijing, China. At least five spikes per accession were pulled apart at physiological maturity. The rachis brittleness phenotype was identified by applying elevated mechanical pressure by hand.

### Chr3D deletion region identification

As the genomic comparison analysis revealed a ~0.8 M segment deletion in chr3D in Zang1817 as compared with CS-IWGSCv1, that the region ranges chr3D:55.5–56.3 Mbp along with CS-IWGSC1 reference genome. We then identified this segment deletion based on the coverage data in all resequence data, and identified this deletion in 50 Tibetan wheat accessions, which mainly in Tibetan semi-wide wheat accession.

### Identification of homologs to barley *Btr1/2* genes

To identify the *Btr1* and *Btr2* homologs in wheat, we used the barley *Btr1/2* gene sequences^31^ as queries to identify the candidate homologs in the assembly of CS-IWGSCv1, Zang1817, *Triticum dicoccoides* WEWseq v1.0^76^, and *Aegilops tauschii* Aet v4.0^12^ using TBLASTN, with the thresholds *E* value <10^−5^ and identity >60%. The candidate homolog genes were aligned with *Btr1* and *Btr2* of barley with MAFFT v7^77^ to filter out the candidates missing crucial domain. The remaining gene sequences were used to conduct BLASTN analyze against each other with the threshold *E* value <10^−5^. In collinearity analysis, the gene pairs were first determined by selecting homologous gene pairs with highest BLAST scores mutually, and then by selecting the rest genes with its single-side best hits.

### TE insertion identification in *TaQ-5A*

The genomic comparison analysis in local region revealed a TE insertion in *TaQ-5A* gene in Zang1817 comparing with CS-IWGSCv1. To inspect the TE insertion in *TaQ-5A* gene in each of the re-sequenced wheat accessions, we scanned all the mapped reads covering the insertion site (chr5A:650129563 on CS-IWGSCv1) for each sample. Samples with at least two perfect matched reads were classified as non-insertion. For the rest samples with soft-clipped reads covering the insertion site, we extracted these soft-clipped reads, and then aligned these sequences in soft-clipped sequences with the 161 bp TE sequence (detected in Zang1817); if the soft-clipped sequences are 100% match with detected TE sequence and length >10 bp, the sample was classified as with-TE insertion. The remaining samples were classified as unknown, considering insufficient statistical power.

### GWAS analysis

GWAS was performed using a mixed linear model (MLM) incorporating population structure and kinship coefficients in TASSEL v5^78,79^ to find genomic sites significantly associated with rachis brittleness. All non-Tibetan samples were classified as non-brittle to increase statistical power. Principal components (PCs) of the association panel were calculated in PLINK v1.90^71^ using the high-quality data for the 35,045,206 variation sites (SNPs or INDELs) with MAF ≥ 0.05 and missing data <30%. The first five PCs were used to estimate population structure. The variance-covariance kinship matrix was estimated in TASSEL. The SNPs (*P* ≤ 2.85 × 10^−10^, Bonferroni-corrected threshold of 0.01) identified in the GWAS were used to declare significant marker-trait association. To identify phenotypic variances explained by chromosome 3D 0.8-Mb deletion and TE-insertion in *TaQ-5A* gene, we produced a pseudo-VCF file containing two sites for each of these two genomic variances, and set genotype of each sample to “0/0” (variant absence) or “1/1” (variant presence) and performed GWAS analyze as mentioned above. To justify population structure bias on chr3B (Supplementary Fig. 27a), we combined the top five PCs for chr3B and the top five PCs for the whole genome together as covariance representing local population structure for performing GWAS analysis specific on chr3B (Supplementary Fig. 27b).

### Genomic region detection responsive to de-domestication

To detect the differentiation regions during de-domestication process of Tibetan semi-wild wheat, two sample groups DE (de-domestication) and DO (domestication) were defined according to phenotypes. DE group contains all Tibet samples with brittle rachis (a part of TS samples, 50 in total). DO group contains the rest Tibet samples (the other part of TS and all TL samples, 52 in total). Further analyses were performed base on these two groups. Considering the frequently introgression events in AB subgenomes from the tetraploid wheat^19^, the top 5% quantiles for AB subgenomes and D subgenome were calculated separately. Differentiation index (*F*_ST_) was calculated using VCFtools v0.1.13^19^ with window size of 100 kb and window step of 100 kb.

Nucleotide diversity (*π*) was estimated using VCFtools with window size of 100 kb. Pi-ratio was further calculated between two groups as (*π*_DO_/*π*_DE_). To reveal the phenotype-associated genomic sequence loss event, we proposed a coverage depth difference based variable, CNV-index, as a counterpart to *F*_ST_ and Pi-ratio methods those are merely based on single nucleotide polymorphism neglecting situations such as genomic segment deletion. CNV-index was defined as the difference of normalized average read depths in two groups in each size-fixed window, to reflect the coverage differences between groups of samples. To calculate CNV-index between DE and DO groups, we first calculated the mean of Normalized Coverage Number (NCN) in each 100 kb window for both groups; Then CNV-index was calculated as (NCN_DO_-NCN_DE_), which is notated as CNV-index. Positive numbers indicate there are more accessions in DE were detected as genomic segment deletion in this window, and vice versa.

### Candidate region and gene identification

The high-altitude (HA) wheat accessions (99 in total) were reserved to accessions gathered in Tibetan Plateau. The LA wheat accessions (73 in total) were selected globally and clearly known to be in LA. The accessions which might be vague to be identified as HA or LA were excluded. The top 5% quantile distributions of *F*_ST_ (AB subgenomes and D subgenome respectively) between HA and LA groups were classified as candidate genetic differentiation regions, and genes residing in these regions were classified as candidate genes for high-altitude adaption. To exclude the possibility that divergence between HA and LA accessions are caused by population structure variations, we performed the *F*_ST_ analysis with randomly shuffled sample-labels repeatedly for 100 times to generate a series of empirical distributions (expected diversity) of *F*_ST_. As the maximum *F*_ST_ scores (0.22 for A&B subgenome, 0.15 for D subgenome, corresponding to the 5% thresholds) in empirical distributions is still below our threshold, indicating that the thresholds for A&B subgenome and D subgenome as the criteria is able to rule out the source of expected genomic diversity.

### Haplotype analysis in response to high-altitude

Classifying the haplotypes of the genotype-associated candidate genes into HA-prone haplotype and LA-prone haplotype helps understanding the adaption of wheat to the high-altitude environment. To gain enough statistical power in showing the haplotype distributions geographically, we merged the resequencing data in this study with a recently published WEC data set (>1000 accessions)^19^. Only SNPs that are polymorphic in both data sets were retained. For each gene, we identify the haplotypes of all samples based on SNPs residing in gene region using haplotype function in R package pegas v0.12^80^. Haplotype harboring mutations that causing amino-acid changes and more enriched in HA groups were classified as HA-haplotype, and the other haplotype were classified as LA-haplotype. Samples or genes without enough sites or poorly covered were discarded when generating the HapMap graph for specific genes.

### Reporting summary

Further information on research design is available in the Nature Research Reporting Summary linked to this article.

## Supplementary information

Supplementary Information

Description of Additional Supplementary Files

Supplementary Data 1

Supplementary Data 2

Supplementary Data 3

Supplementary Data 4

Supplementary Data 5

Reporting Summary

## Data Availability

Data supporting the findings of this work are available within the paper and its Supplementary Information files. A reporting summary for this Article is available as a Supplementary Information file. The data sets generated and analyzed during the current study and genetic material used for this paper are available from the corresponding author upon request. The genomic sequencing reads and RNA-seq reads have been deposited to NCBI Sequence Read Archive with accession PRJNA596843 and PRJNA596859, respectively. Zang1817 genome assembly has been deposited at DDBJ/ENA/GenBank under the accession JACSIS000000000. The version described in this paper is version JACSIS010000000. The genome assembly and full annotation data are also available from website http://wheat.cau.edu.cn/Zang1817_genome/. The raw reads of 73 previously published re-sequenced accessions are available under NCBI Sequence Read Archive accession PRJNA476679 and https://downloads-qcif.bioplatforms.com/bpa/wheat_cultivars/cultivars/. The whole-exome capture data set containing 1,026 hexaploid wheat accessions is downloaded from http://wheatgenomics.plantpath.ksu.edu/1000EC/. Pfam databases used in this study are available at ftp://ftp.ebi.ac.uk/pub/databases/Pfam/releases/Pfam27.0/. KEGG databases used in this study are available at https://www.kegg.jp/kegg/download/. Source data are provided with this paper.

## Source data

Source Data

## Code availability

The source code and scripts used in the paper have been deposited in GitHub [https://github.com/wangzihell/CAU-TibetanWheatSeq].
