# Supplementary material for: Origin and adaptation to high altitude of Tibetan semi-wild wheat
Source: Nat Commun. 2020 Oct 8;11:5085. doi: 10.1038/s41467-020-18738-5 (PMC7545183; doi:10.1038/s41467-020-18738-5)
Supplement: Supplementary file 1 — Supplementary Information [file 41467_2020_18738_MOESM1_ESM.pdf]

Origin and adaptation to high altitude of Tibetan semi-wild wheat

Guo *et al.*

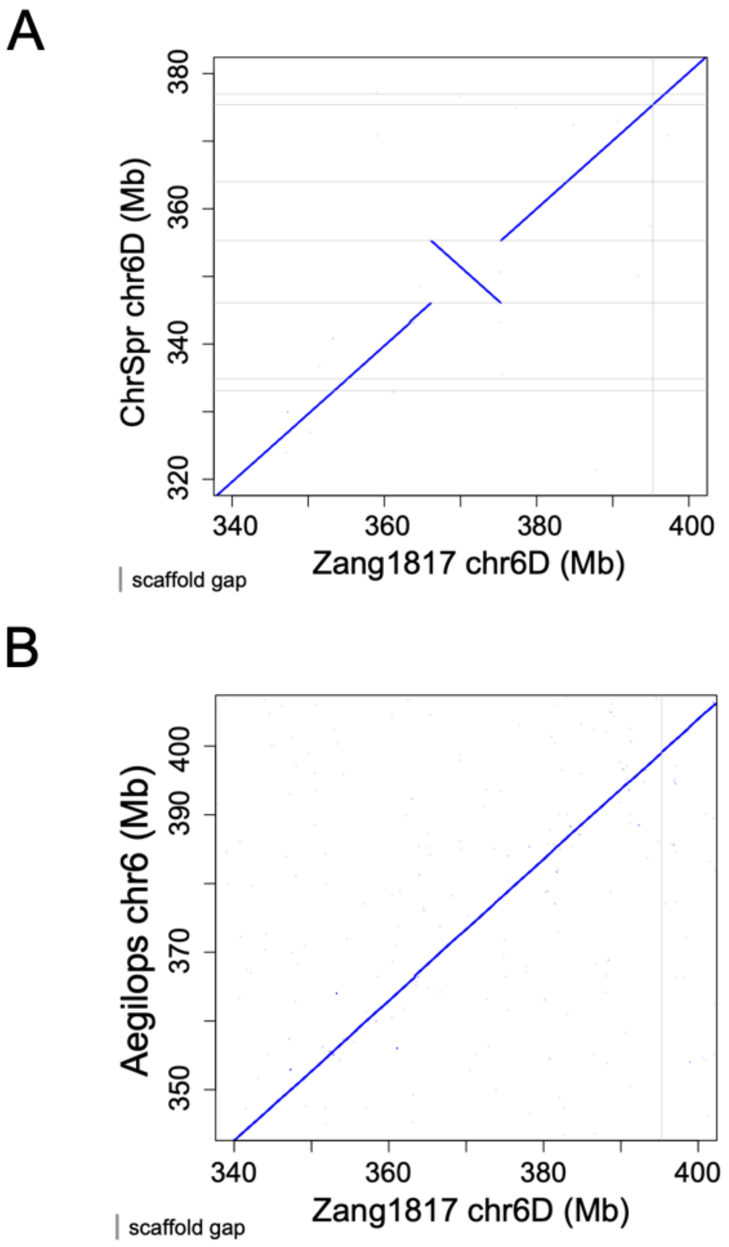

Supplementary Figure 1. Dot plot reveals the collinearity of chromosome 6D region between Zang1817 and CS as well as between Zang1817 and *Aegilops*. (A) The chr6D: 366.14M~375.29M region of Zang1817 assembly showesd genomic inversion compared with chr6D: 346.09-355.29M region of CS genome. (B) The chr6D: 366.14M~375.29M region of Zang1817 assembly exhibites fine collinearity compared with 369.43M~378.71M region of *Aegilops* reference genome. Grey lines indicate the scaffold boundaries.

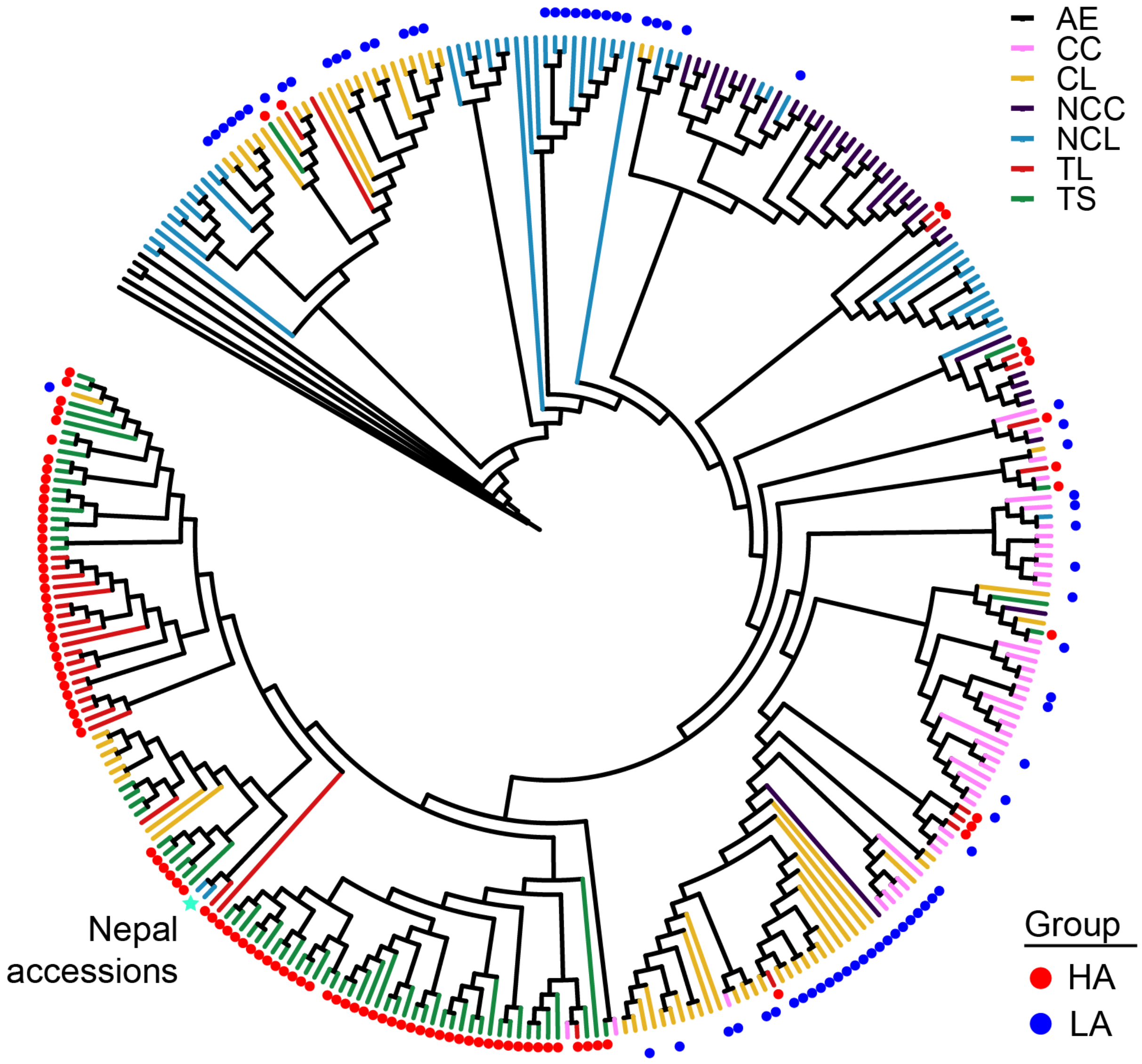

Supplementary Figure 2. A phylogenetic tree plot inferred using SNPs in D subgenome. HA: high-altitude accessions (red dots), LA: low-altitude accessions (cyan dots). Two Nepal accessions were labeled by star.

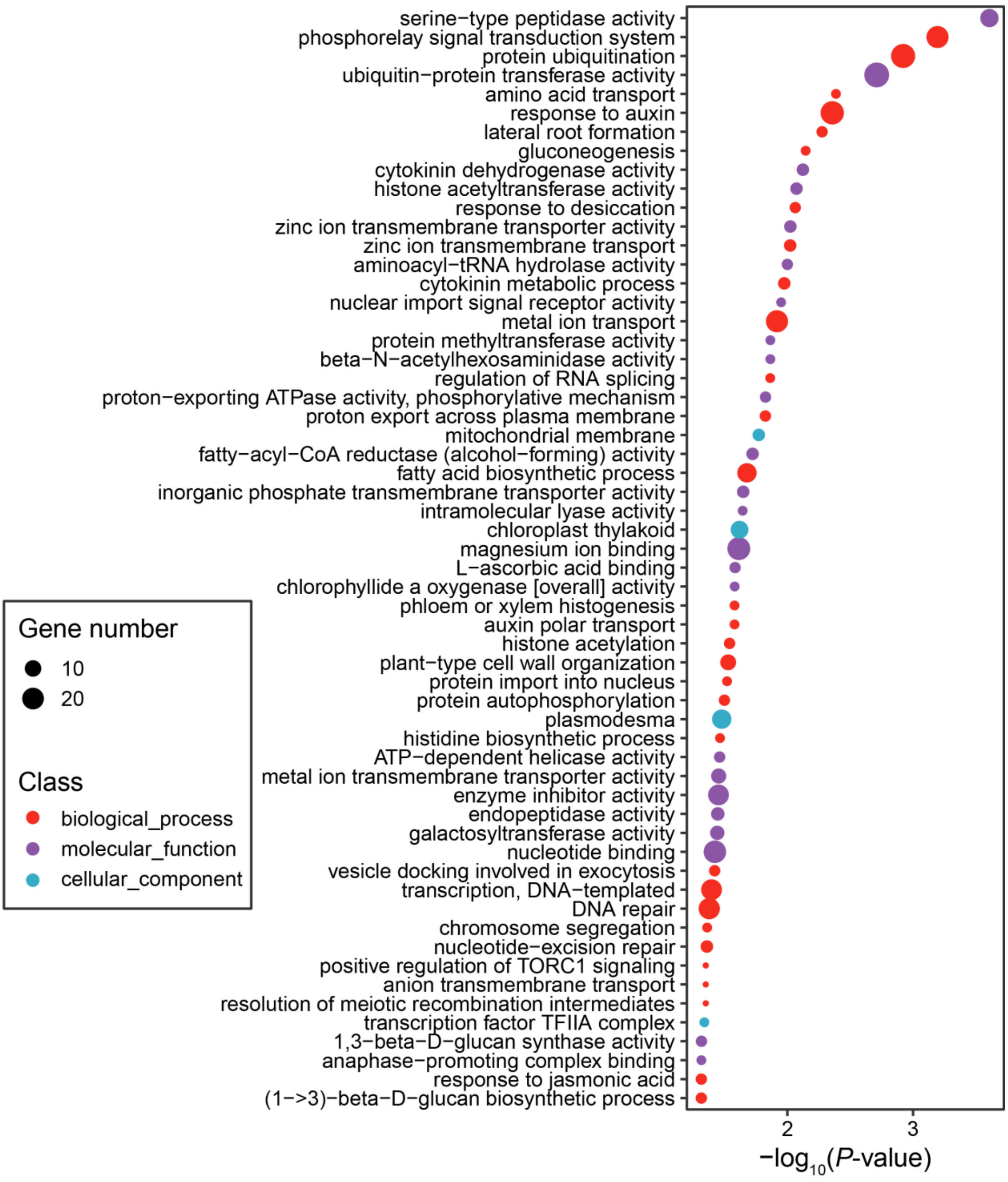

Supplementary Figure 3. GO enrichment analysis for the genes located in the significant divergent genomic regions of F_{ST} analysis between high-altitude (HA) wheat accessions and low-altitude (LA) wheat accessions. Color indicate the classes of GO items. Circle sizes indicated gene numbers overlapped with the GO item group. X-axis, $-\log_{10}$(*P*-value). The items with *P*-value less than 0.05 were shown. Source data are provided as a Source Data file.

```
TraesCS2A02G142800.1      ------------------------------------------------------------  0
Os01t0174000-00           MISLSHSISKNIFVSTRVGVTIHFFSKKKYIYYSNPLYPQRAHMAHSFRSVRERQVVLRF  60
AT5G11260.1               ------------------------------------------------------------  0
Solyc08g061130.3.1        ------------------------------------------------------------  0


TraesCS2A02G142800.1      --------------------------------------------MAIAEASPF-ADLPFP  15
Os01t0174000-00           YEIGWMGMPLRFRGGHRQMRWMRVWCLRTPRSPNPHRRMAPEWTARMTATSPSKTPQPHR  120
AT5G11260.1               ------------------------------------------------------------  0
Solyc08g061130.3.1        ------------------------------------------------------------  0


TraesCS2A02G142800.1      DDLPEFPHG---PVGDD----DAFAL----DGFDL--DDLDIDFDFDLDLD----LLPTD  58
Os01t0174000-00           PLLPISPHLSLSPPIPQQSTQTRFLSPPLSRFLSLSSHPICICFSFPHLCFVFVVVSSSD  180
AT5G11260.1               --------------MQEQAT----------SSLA--------ASSLPSSSERSSSSAPHL  28
Solyc08g061130.3.1        --------------MQEQAT----------SSIA--------ASSLPSSSERSSSSALHH  28
                                          :               :           .:

TraesCS2A02G142800.1      --------DVQLPSPPPPLATSSSSAGSPGGAGDSSSGSGAGADGGLKNDESSET-----  105
Os01t0174000-00           SVAAGVESDEEIRR-VPEM---------GGGGGSASSGAGADERQGKEDGKQQGGGGGGA  230
AT5G11260.1               EIKEGIESDEEIRR-VPEF---------GGEAVGKETSGR-----------------ESG  61
Solyc08g061130.3.1        ELKEGMESDDEIRR-VPEM---------GGEATGTTSASGR----------------DGV  62
                                  * ::    * :          * . .  :..

TraesCS2A02G142800.1      ------SSRSASAGSD-GKAMNGEGEDDKRRARLVRNRESAHLSRQRKKQYVEELEGKVK  158
Os01t0174000-00           AAAGGGQ---EQAPPARKRGRSAGDKEQNRLKRLLRNRVSAQQARERKKAYMTELEAKAK  287
AT5G11260.1               SATGQERTQATVGESQRKRGRTPAEKENKRLKRLLRNRVSAQQARERKKAYLSELENRVK  121
Solyc08g061130.3.1        SAAGQAQ---PSAGTQRKRGRSPADKENKRLKRLLRNRVSAQQARERKKAYLIDLEARVK  119
                                      .     :. .   ::::*  **:*** **: :*:*** *:  :** :.*

TraesCS2A02G142800.1      AMQATIADLSTRISCVTAENAALKQQLAGAG---------GAGV--PPPLPMYPGLYPLP  207
Os01t0174000-00           DLELRNAELEQRVSTLQNENNTLRQILKNTTAHAGKRGGGGGGKGGDGGGGGKKHHFTKS  347
AT5G11260.1               DLENKNSELEERLSTLQNENQMLRHILKNTTGNKR--GGGGGSNA-DASL----------  168
Solyc08g061130.3.1        ELETKNAELEERLSTLQNENQMLRHILKNTTAGAQ--EGRK-------------------  158
                           ::   ::*. *:* :  **  *:: * .:

TraesCS2A02G142800.1      LPWMHPAYAMGAHGSQVPLMPIPRLKTKQPASAAAEPPAKKSRKTKKVASVSLLGLLFLM  267
Os01t0174000-00           ------------------------------------------------------------  347
AT5G11260.1               ------------------------------------------------------------  168
Solyc08g061130.3.1        ------------------------------------------------------------  158


TraesCS2A02G142800.1      MLCGCLVPAVNRMYGAVDSREGIVLGPSQSRHGRVLAVDGPRDGVLEGVDSKLPHNSSET  327
Os01t0174000-00           ------------------------------------------------------------  347
AT5G11260.1               ------------------------------------------------------------  168
Solyc08g061130.3.1        ------------------------------------------------------------  158


TraesCS2A02G142800.1      LPALLYIPRNGKHVKINVNLVIQSVVASEKASSRMCHSDGKTSCNQGQEDTSLAIPGHVA  387
Os01t0174000-00           ------------------------------------------------------------  347
AT5G11260.1               ------------------------------------------------------------  168
Solyc08g061130.3.1        ------------------------------------------------------------  158


TraesCS2A02G142800.1      QLNSGEVMESAKAIKNKLMALPPGDGSIYRDDDELLPQWFSEAMSGPMLSSGMCTEVFQF  447
Os01t0174000-00           ------------------------------------------------------------  347
AT5G11260.1               ------------------------------------------------------------  168
Solyc08g061130.3.1        ------------------------------------------------------------  158


TraesCS2A02G142800.1      DISPTTIVPVYSSGMHNASHNSTENLTSSQSDKVKNRRILHSMAVPLKGSTSNHTDHLKA  507
Os01t0174000-00           ------------------------------------------------------------  347
AT5G11260.1               ------------------------------------------------------------  168
Solyc08g061130.3.1        ------------------------------------------------------------  158


TraesCS2A02G142800.1      HPKNESFAGNKPASSVVVSVLADPRVDADGRISSKSLSRIFVVVLVDSVKYVTYSCVLPF  567
Os01t0174000-00           ------------------------------------------------------------  347
AT5G11260.1               ------------------------------------------------------------  168
Solyc08g061130.3.1        ------------------------------------------------------------  158
```

```
TraesCS2A02G142800.1      KTHSPHL      574
Os01t0174000-00           -------      347
AT5G11260.1               -------      168
Solyc08g061130.3.1        -------      158
```

Supplementary Figure 4. Protein sequence alignment of TraesCS2A02G142800 (*TaHY5-like*) and homologs of *HY5* in rice (Os01t0174000), Arabidopsis (AT5G11260) and soybean (Solyc08g061130). Conserved domain, denoted by red lines, was predicted using NCBI Conserved Domain Database. Alignment was performed using EMBOSS Clustal Omega web server.

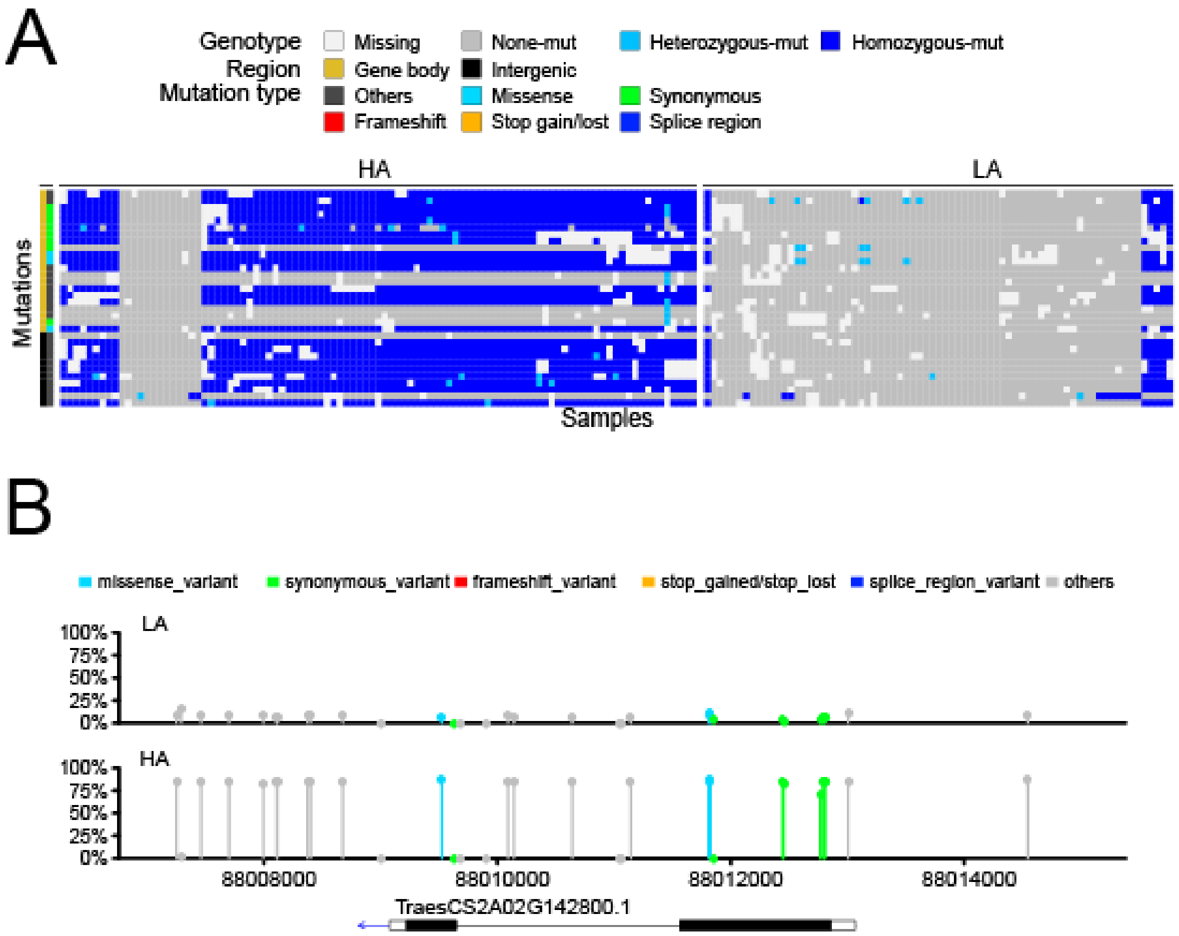

Supplementary Figure 5. Divergent haplotypes of *TaHY5-like* gene (TraesCS2A02G142800) in response to high-altitude environmental extremes shown in heatmap (A) and lollipop graph (B). In heatmap plot (Panel A), first column from left indicates genomic regions of *TaHY5-like* gene and the second column from left indicates mutation types, whereas other columns indicate different wheat accessions; rows indicate SNP/InDel variations; In lollipop graph (Panel B), y-axis indicates mutation frequency in HA and LA wheat accessions; mutation types were distinguished by different colors. HA, high-altitude wheat accessions, LA, low-altitude accessions. Source data are provided as a Source Data file.

```
TraesCS2B02G244300.1        MSSSSRVRVGTLVPFAEGKSGSPNASPPMPSIPILEGSNVVGRSNLVAVDKRVSRKHLSL 60
AT5G15170.1                 ---MAHSQVAYLIPLKADLKE--D--NSSPRITLSEGPNIIGRGNVSIVDKRLSRKHITI 53
OIT34941                    MSTSSQVQVGFLVPLSDNLEE--DEKSSIPKIPLSEGLNWVGRDCIPVTDKRLSRKHLTV 58
OTF90890                    --MEHSSQVGVLVPLSDNLDE--DASNSIPKVQISKGVNILGRDVIPITDKRISRKHLSV 56
                                   :*. *:*:  . .   :     * : : :* * :**. :  .***:****:::

TraesCS2B02G244300.1        RALPDGSVEVVVEGPNPIVVRSEG--QRRKVCAQQKAKVMHDDVLELIPGEYFMKYVNTS 118
AT5G15170.1                 IVSTSGSASLSVDGTNPVVIRSSGDGERKKVKPSEEVSVCNDDLIELIPGHHFFKLVLLN 113
OIT34941                    TATSTGSADVLVEGTNPVVIRLKG--ERKKLLSGERWKIESGDVIELIPGHYLFRYVSTA 116
OTF90890                    TVSDDGSVEVAVEGTNPVVIRSQ---QRKKLLSREKLTIKDGDIIELIPGHYIFKYKAFN 113
                             .   **..: *:* **:*:* .   :*:*:   :. .:  .*::*****.::::

TraesCS2B02G244300.1        DERKSSTSV-ESHDLKKGKRHSEEDSVAAKRNRQVMEDEALARTLQESFAEESTSVTEVL 177
AT5G15170.1                 GRAAK----------------------------------------------KAR------ 121
OIT34941                    PKDETSTINKQKRPFSEESITDKGQMHGKKKAREVFQEEASEK---NMLHNNGQ------ 167
OTF90890                    GVKKRYSPNKQKRPLIEGVQDESL---AQKRSRQVEEDEALARKLQSEMNDDIQ------ 164
                                                                               .

TraesCS2B02G244300.1        SSLDSVGSSERNKERTDSVGPLKDVLPLTFRLMRVQGLPSWTNTSTVTIQDVIQGEVLLA 237
AT5G15170.1                 -------KAEDDVEAIRRFCPPNEKLPSTFRLLSVDALPDWANTSCVSINDVIEGDVVAA 174
OIT34941                    -------ETESSAEAIRQFRVPKHKLPLTFRLMRVRELPAWANTEAVSINDVVQGNVLVA 220
OTF90890                    -------DKEIFVEAIRDFKPPKDKIPLTFRLMRVKGLQPWANTNSVSIGDVIQGNVFVA 217
                                   . *   *    .   :. :* ****: *  *  *:**. *:* **::*:*. *

TraesCS2B02G244300.1        VLSNYMVDMDWLLTACPSLRKVPHVLVLHGEDGASLEHLKKTKPANWILHKPPLPISFGT 297
AT5G15170.1                 ILSNYMVDIDWLMSACPKLANIPQVMVIHGEGDGRQEYIQRKKPANWILHKPRLPISFGT 234
OIT34941                    ILSNYMVDMDWLLSACPALKRIPNVLVIHGEGDGVMEHMKRSKPANWILHKPSLPIAYGT 280
OTF90890                    VLSNYMVDLDWLISECPTLKKIPHVLVVHGESDGRQEHMKRNKPSNWILHKPPLPIAWGT 277
                            :*******:***:: ** * .:*:*:*:***...  *::::.**:******* ***::**

TraesCS2B02G244300.1        HHSKAMLLVYPQGIRVVVHTANLIHVDWNNKSQGLWTQDFPWKEAN-DMSTNIGFENDLV 356
AT5G15170.1                 HHSKAIFLVYPRGVRVVVHTANLIHVDWNNKSQGLWMQDFPWKDDDKDPPKGCGFEGDLI 294
OIT34941                    HHSKAMILVYPTGVRVIVHTANLIYVDWNNKSQALWMQDFPWKDQN-NLGKDGGFENDLV 339
OTF90890                    HHSKAMFLVYPQGVRIIVHTANLIYVDWNNKSQGLWMQDFPWKHDG--SDKGSGFESDLI 335
                            *****::**** *:*::*******:********.** ******. .    .. ***.**:

TraesCS2B02G244300.1        DYLRALKWPEFRVNLPVAGDVNINAAFFRKFDYSSSTVRLIGSVPGYHVGPNMKKWGHMK 416
AT5G15170.1                 DYLNVLKWPEFTANLPGRGNVKINAAFFKKFDYSDATVRLIASVPGYHTGFNLNKWGHMK 354
OIT34941                    DYLSVLKWPEFSANIPAFGNCKINSLFFKRFDYSSAAVRLIASVPGYHSGPSLKKWGHMK 399
OTF90890                    DYLSALKLPEFSVQFPERGTFSLNSSFFKKFDYSKATVRLIASVPGYHSGSNIKKWGHMK 395
                            *** .** *** .::*  *  .:*: **::****.::****.****** * .::******

TraesCS2B02G244300.1        LRSVLEECVFEKQFCKSPLIYQFSSLGSLDEKWMSEFACSLSAGKADDGSQLGIGKPLIV 476
AT5G15170.1                 LRTILQECIFDREFRRSPLIYQFSSLGSLDEKWLAEFGNSLSSGITEDKTPLGPGDSLII 414
OIT34941                    LRTILQECTFSKEFQKSPLIYQFSSLGSLDEKWMAELASSMSAGVSEDKRPLGIGEPLIV 459
OTF90890                    IRSVLQDCVFDKEFRNAPLIYQFSSLGSLDEKWMAEFAFSMSSGVSDDKKPLGLGEPLIV 455
                            :*::*::* *.::* .:****************::*:. *:*:* ::*   ** *. **:

TraesCS2B02G244300.1        WPTVEDVRCSIEGYAAGSCIPSPQKNVEKDFLRKYWSRWKADHVGRCRAMPHIKTFTRYN 536
AT5G15170.1                 WPTVEDVRCSLEGYAAGNAIPSPLKNVEKPFLKKYWARWKADHSARGRAMPHIKTFTRYN 474
OIT34941                    WPNVEDVRCSLEGYAAGNAVPSPLKNVEKEFLKKYWARWKASHTGRCRAMPHIKTFLRYN 519
OTF90890                    WPTVEDIRCSLEGYAAGSCVPSPQKNVEKAFLKKYWGKWKATHTGRCRAMPHIKTFTRYN 515
                            **.***:***:******..:*** ***** **:***.:*** * .* ********* ***

TraesCS2B02G244300.1        GQNIAWFLLTSSNLSKAAWGALQKNNSQLMIRSYELGVLFLPKTLQ-SVPQFSCTEKSRS 595
AT5G15170.1                 DQKIAWFLLTSSNLSKAAWGALQKNNSQLMIRSYELGVLFLPSPIKTQGCVFSCTESNPS 534
OIT34941                    GQSLAWLLLTSSNLSKAAWGSLQKNNSQLMIRSYELGVLFLPSSVK-RGCGFSCTDNGYP 578
OTF90890                    GQNLAWFLLTSSNLSKAAWGALQKNNTQLMIRSYELGVLFLPSSTS-HAHVFSCTENGST 574
                            .*.:**:*************:*****:***************.  .     ****:..

TraesCS2B02G244300.1        NLDKL-----ALGKNIKTKLVTLCWKGDEEKDPSAEVVRLPVPYQLPPQLYGPEDVPWSW 650
AT5G15170.1                 VMK--AKQETKDEVEKRSKLVTMTWQGDR---DLPEIISLPVPYQLPPKPYSPEDVPWSW 589
OIT34941                    S-----GEETSMHEGKRIKLVTLAWKGKEN-DDSAEVVKLPVPYELPPKPYSPEDIPWSW 632
OTF90890                    SENKYSGSPLKTHEAKRTKLVTLASPHSKK-MHLPDVVTLPVPYELPPARYSSEDVPWSW 633
                                            : ****:    ..      ::: *****:***  *. **:****

TraesCS2B02G244300.1        DRRYTKKDVYGSVWPRHG--------    668
AT5G15170.1                 DRGYSKKDVYGQVWPR----------    605
OIT34941                    DRRYTKKDVYGQVWPRQVKLYTNQDS    658
OTF90890                    DKQYKKRDVLGQVWP-----------    648
                            *: *.*:** *.***
```

Supplementary Figure 6. Protein sequence alignment of TraesCS2B02G244300 (*TaTDP1*) and homologs of *TDP1* in *Arabidopsis thaliana* (AT5G15170)*, Nicotiana attenuate* (OIT34941) and *Helianthus annuus* (OTF9-890). Conserved domain, denoted by red lines, was predicted on NCBI Conserved Domain Database. Alignment was performed using EMBOSS Clustal Omega web server.

```
TraesCS2B02G371600.1    ------MGGSSACDGTLSLDEFNASAKALVTKWRGIDVDDALPDWEWRPCRKMGV---PS    51
OTG19232                --MAVMVSNNATCDGSLSSTDFQIVANAFAEKWKKFN--SDFPEWLWIDCSNRLGFAAHG    56
AT3G07525.1             MDS-----AREVSDGRLTVEGFSVASRAFADKWKIHN--QSFPPWSWVPLINRTLLVSKK    53
OIT32125                MSSDKAIINISTWDGTITSAEFHVAAANFAEQWNNLD--LGFPHWSWINCPKGPRFAASK    58
                                   . ** ::   *   :  :. :*.  :    :* * *    :

TraesCS2B02G371600.1    ETEGFVALEGVYRIGAGGQVEGSSDDP----------VPDDGVEYDTSVQSSRDNVHLYD    101
OTG19232                VSDGYLSVQNVFFKVQSSLKEEHGECSSN--------DYEEPFDNATLVQSTSDDGHWYD    108
AT3G07525.1             -EEGYLSLEKIIIL--SSLEEEIPEDESL-NVATDCLEKEETVDHTILVPTMENEAHYYD    109
OIT32125                -VEGYLSLENMILP--GSTEEDRNQCGLAGIVDLSCSNEDVFTDTAILVQKHSQERHHYD    115
                          :*::::: :     ..  *   :              :   :    * .  :: * **

TraesCS2B02G371600.1    FHIVYSFSYKVPVLYFRGLQSGGQLLTLDEIKKDLPSHSLQLLNESKWTFITREEHPHLS    161
OTG19232                FHIVYSSSYNVPVLYFRAHSSDGQLLNQDEIEKNLPTESAKVLTESKWTFITQQEHPYLN    168
AT3G07525.1             FHIVYSASYKVPVLYFRGYCSGGEPLALDVIKKDVPSCSVSLLLESKWTFITQEEHPYLN    169
OIT32125                FHIVYSSSFRVPVLYFRAYCSDGEPLAIEDLEKEFPAYTTQELAISKWTFITQEEHPYLN    175
                        ****** *:.*******.  *.*: *  : ::*:.*: : . *  *******::***:*.

TraesCS2B02G371600.1    RPWFTLHPCGTSDWMKLLLHKPG-----DKDRSLQYLSAWLSVAGQAVGLKIPLKLYCSS    216
OTG19232                RPWYTLHPCGTSEWMKLLLAGENST---AENRAEQYLISWFSVVGQVFGLKLPLDMVKSD    225
AT3G07525.1             RPWFKLHPCGTEDWIKLLSQSSSS--SGCQMPIVLYLVSWFSVVGQVVGLRIPLEMLN--    225
OIT32125                RPWYTLHPCGTGDWMKLLFSNEHSVVSQGGVAVEKYLISWFSVICPIFGLKIPLKLFLNA    235
                        ***:.****** :*:***                 ** :*:**    .**::**.:

TraesCS2B02G371600.1    -----------   216
OTG19232                G----------   226
AT3G07525.1             -----------   225
OIT32125                SNKSENVSYVT   246
```

Supplementary Figure 7. Protein sequence alignment of TraesCS2B02G371600 (*TaATG10*) and homologs of *ATG10* in *Arabidopsis thaliana* (AT3G07525), *Nicotiana attenuate* (OIT32125) and *Helianthus annuus* (OTG19232). Conserved domain, denoted by red lines, was predicted on NCBI Conserved Domain Database. Alignment was performed using EMBOSS Clustal Omega web server.

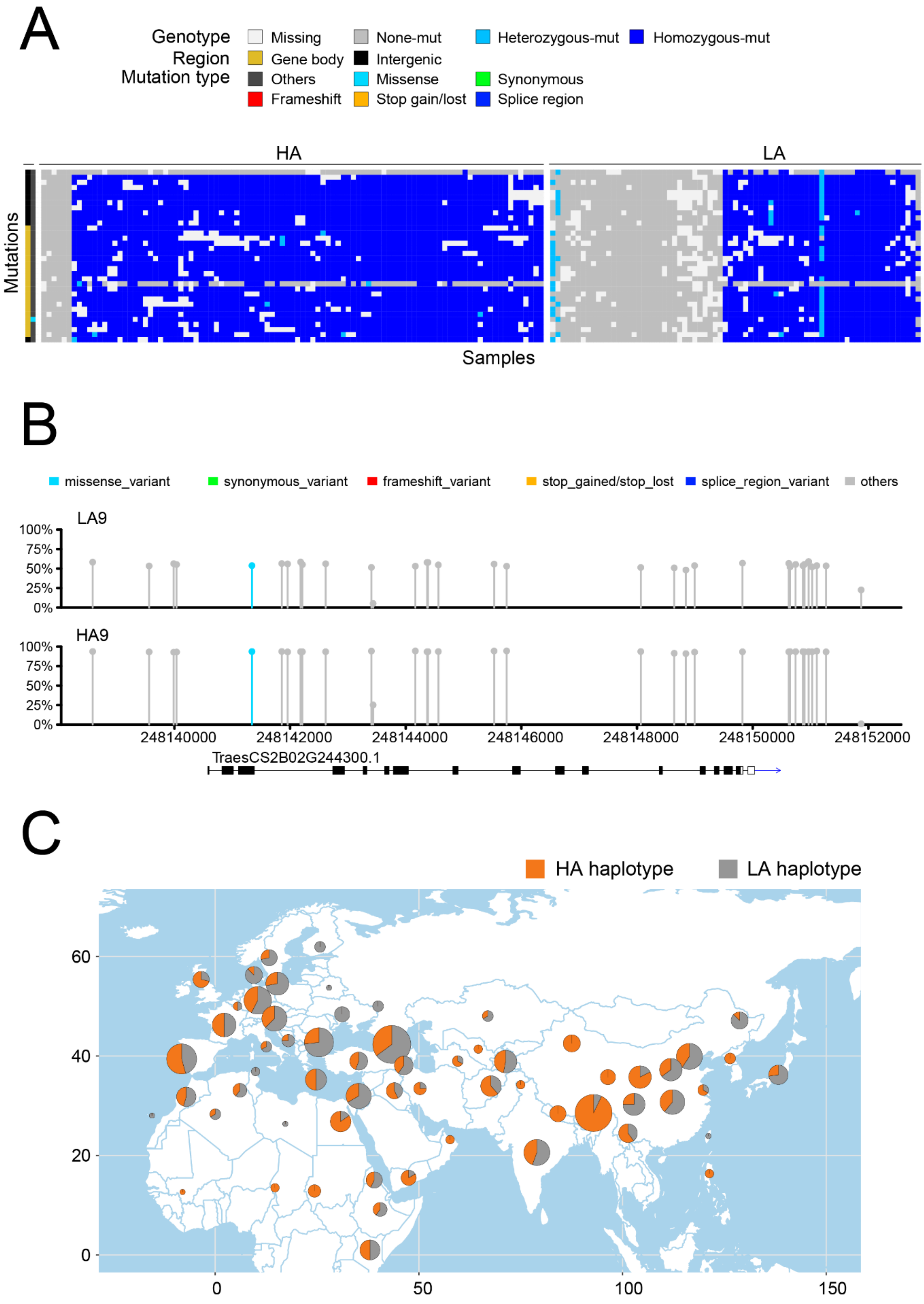

A
Genotype
Region
Mutation type
Missing
None-mut
Heterozygous-mut
Homozygous-mut
Gene body
Intergenic
Others
Missense
Synonymous
Frameshift
Stop gain/lost
Splice region
HA
LA
Mutations
Samples
B
missense_variant
synonymous_variant
frameshift_variant
stop_gained/stop_lost
splice_region_variant
others
LA9
HA9
100%
75%
50%
25%
0%
248140000
248142000
248144000
248146000
248148000
248150000
248152000
TraesCS2B02G244300.1
C
HA haplotype
LA haplotype
60
40
20
0
0
50
100
150

Supplementary Figure 8. Divergent haplotypes of *TaTDP1* (TraesCS2B02G244300) in response to high-altitude environmental extremes shown in heatmap (A), lollipop graph (B) and HapMap chart (C). In heatmap plot (Panel A), first column from left indicates genomic regions of *TaTDP1* gene and the second column from left indicates mutation types, whereas other columns indicate different wheat accessions; rows indicate SNP/InDel variations; In lollipop graph (Panel B), y-axis indicates mutation frequency in HA and LA wheat accessions; mutation types were distinguished by different colors. In HapMap graph (Panel C), geographic distribution of different haplotypes of *TaTDP1* gene. The haplotype distribution analysis was determined by using 308 resequencing accessions and 1,026 wheat exome-capture samples. Orange indicates the proportion of HA adapted haplotype of *TaTDP1* gene. HA, high-altitude wheat accessions, LA, low-altitude accessions. Geographic map in C was generated using R packages, including maps, ggmaps, ggplot2 and sf, with geographic information data edited from original version of the Natural Earth project database (version 2013). Source data are provided as a Source Data file.

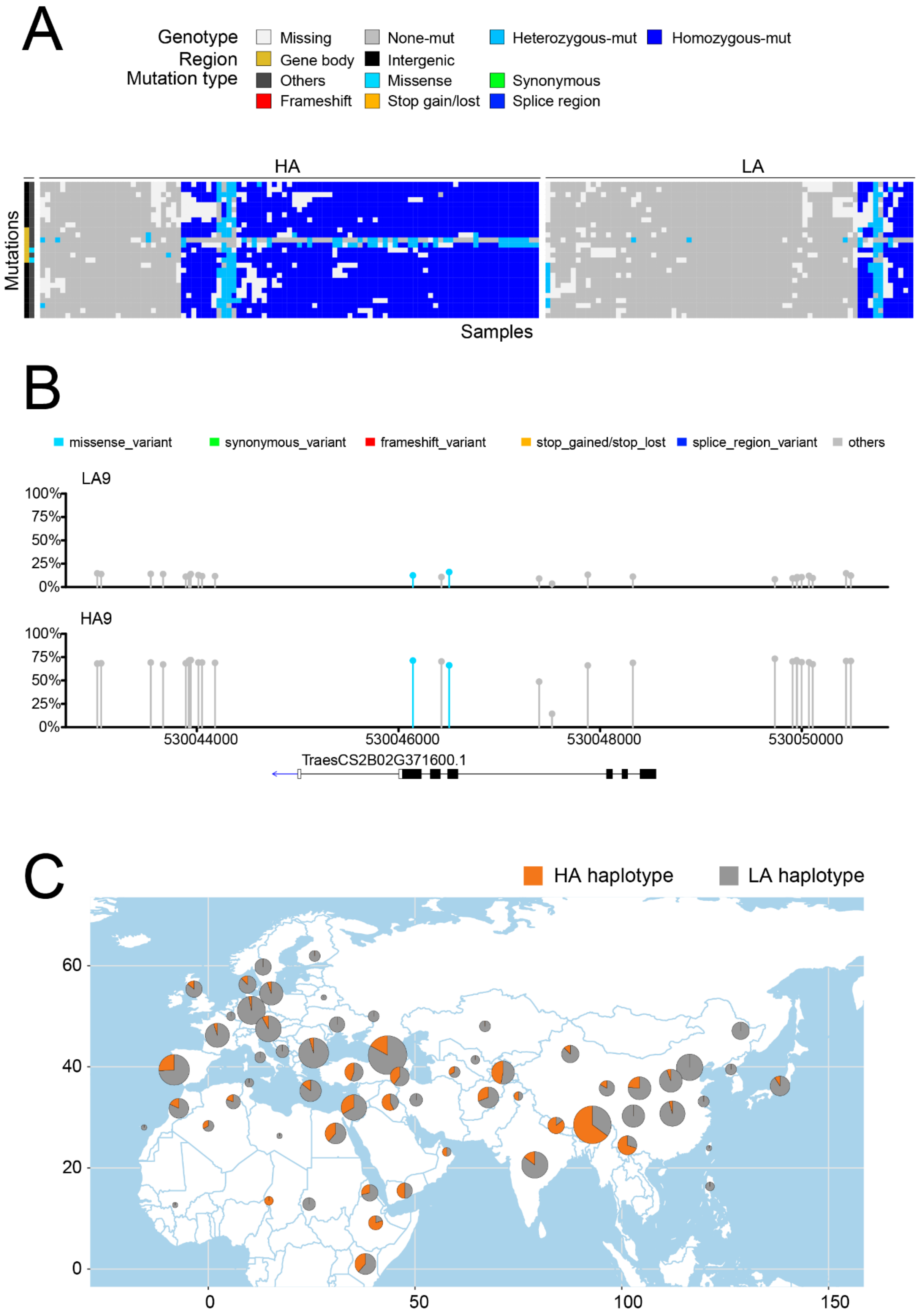
A
Genotype
Missing
None-mut
Heterozygous-mut
Homozygous-mut
Region
Gene body
Intergenic
Mutation type
Others
Missense
Synonymous
Frameshift
Stop gain/lost
Splice region
HA
LA
Mutations
Samples
B
missense_variant
synonymous_variant
frameshift_variant
stop_gained/stop_lost
splice_region_variant
others
LA9
HA9
100%
75%
50%
25%
0%
530044000
530046000
530048000
530050000
TraesCS2B02G371600.1
C
HA haplotype
LA haplotype
60
40
20
0
0
50
100
150

Supplementary Figure 9. Divergent haplotypes of *TaATG10* (TraesCS2B02G371600) in response to high-altitude environmental extremes shown in heatmap (A), lollipop graph (B) and HapMap chart (C). In heatmap plot (Panel A), first column from left indicates genomic regions of *TaATG10* gene and the second column from left indicates mutation types, whereas other columns indicate different wheat accessions; rows indicate SNP/InDel variations; In lollipop graph (Panel B), y-axis indicates mutation frequency in HA and LA wheat accessions; mutation types were distinguished by different colors. In HapMap graph (Panel C), geographic distribution of different haplotypes of *TaATG10* gene. The haplotype distribution analysis was determined by using 308 resequencing accessions and 1,026 wheat exome-capture samples. Orange indicates the proportion of HA adapted haplotype of *TaATG10* gene. HA, high-altitude wheat accessions, LA, low-altitude accessions. Geographic map in C was generated using R packages, including maps, ggmaps, ggplot2 and sf, with geographic information data edited from original version of the Natural Earth project database (version 2013). Source data are provided as a Source Data file.

```
Os12t0582900-01             QPHSLTQAKLAIDRSSSMPPSMATARVASPSTDRKRKASSEDDIDAAAAAAALTSPLAPA  60
AT3G15210.1                 ----------------------------------------MAKM-GLKPDPATTNQTHNN  19
TraesCS3B02G357500.1        -----------------MAPRAAEKAPV----------SPPTRL-GLG----VGGGVGVV  28
Zm00001d000339_T001         -----------------MAPRTSEKTMA-PAA------AAATGL-ALS----VGG--GGG  29
                                                                       : .        .

Os12t0582900-01             RGRKRFVGVRQRPSGRWVAEIKDTIQKIRVWLGTFDTAEEAARAYDEAACLLRGANTRTN  120
AT3G15210.1                 AKEIRYRGVRKRPWGRYAAEIRDPGKKTRVWLGTFDTAEEAARAYDTAARDFRGAKAKTN  79
TraesCS3B02G357500.1        AGGAHYRGVRKRPWGRYAAEIRDPAKKSRVWLGTYDTAEEAARAYDTAAREFRGAKAKTN  88
Zm00001d000339_T001         AGGPHYRGVRKRPWGRYAAEIRDPAKKSRVWLGTYDTAEDAARAYDAAAREYRGAKAKTN  89
                                :: ***:** **:.***:*  :* ******:****:****** **   ***:::**

Os12t0582900-01             FWPRPSPPPAAVAPPETTTTPALPSKVTNLLLLRLKARNQQVVDAGAAAPQEAALLQLQQ  180
AT3G15210.1                 FPTFLELSDQKVPTG-FARSPSQSSTL-----------------DCASPPTLVVPSATAG  121
TraesCS3B02G357500.1        FPFPSSSSSSPVAGG-GS--PSSNSTLDSS--------GGG-SGGCAQAPMQA------I  130
Zm00001d000339_T001         FPYPSCVPLSA---A-GC--RSSNSST-----------VES-FSSDAQAPMQA------M  125
                            *                    :  *.                    *  *  .

Os12t0582900-01             QTPASCQEVFVRSHGEEYGFHVDDFLSDACSNEQYSGDSSLGLDDDDGEVEDEEEEELDF  240
AT3G15210.1                 NVP----------------------------------------------------PQLEL  129
TraesCS3B02G357500.1        PLP----------------------------------------------------PALDL  138
Zm00001d000339_T001         PLP----------------------------------------------------PSLEL  133
                              *                                                      *::

Os12t0582900-01             QFMDVAPGASSAAAEAGLGGEGALCSPFEMVAAELGGA------------------VDVA  282
AT3G15210.1                 SLG---------------GGGGGSCYQ---IPM-----SRPVYFLDLMGIGNVGRGQPPP  166
TraesCS3B02G357500.1        DLFH------RAAAVTAV-AGGGMRFPFNGYPVASRQPLHPYFFYEQAAAAA-------A  184
Zm00001d000339_T001         DLFH------RAAAAATGTGAAAVRFPFGSIPV-----THPYYFFGQAAAAA--------  174
                            .:                 . ..

Os12t0582900-01             AHDAMRQMDYERKISASLYALTGVSECLRIRAAAGATAAARDHLTGLREACRKKQKFAAA  342
AT3G15210.1                 VTSAFRSPVVHVATKMAC-GAQSDSDSSSVVDFEGG-------------MEKRSQLLDLD  212
TraesCS3B02G357500.1        AASGYRALKVAQPVTVAA-VAQSDSDSSSVVDLSPSPPA--------V-TAHKAVTFDLD  234
Zm00001d000339_T001         AEAGCRVLKLAPAV---T-VAQSDSDCSSVVDLSPSPPA--------AVSARKPAAFDLD  222
                            .  . *                . *:.  :     .               ::   :

Os12t0582900-01             AAAPPQQQEPSPPPQQPETPASSGGGMSETASGGGGDGDVLMWSSLDLAPICHMA       397
AT3G15210.1                 LNL--------PPPSEQA-------------------------------------       222
TraesCS3B02G357500.1        LNG--------PPPSED--------------------------------------       243
Zm00001d000339_T001         LNC--------SPPTEAEA------------------------------------       233
                                        ** :
```

Supplementary Figure 10. Protein sequence alignment of TraesCS3B02G357500 (*TaERF4*) and homologs of *ERF4* in *Arabidopsis thaliana* (AT3G15210.1), *Oryza sativa* Japonica (Os12g0582900) and *Zea mays* (Zm00001d000339_T001). Conserved domain, denoted by red lines, was predicted on NCBI Conserved Domain Database. Alignment was performed using EMBOSS Clustal Omega web server.

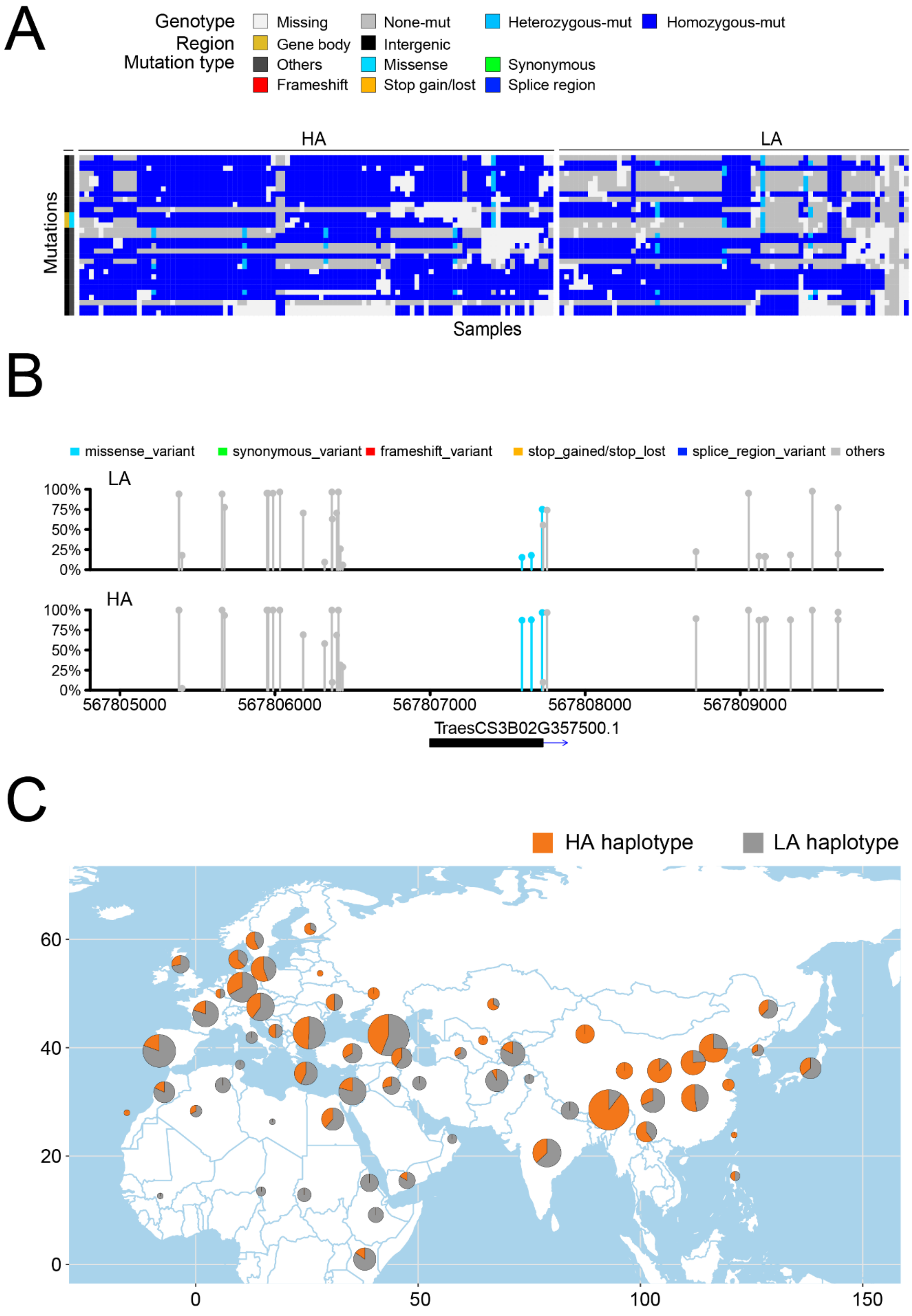
A
Genotype
Missing
None-mut
Heterozygous-mut
Homozygous-mut
Region
Gene body
Intergenic
Mutation type
Others
Missense
Synonymous
Frameshift
Stop gain/lost
Splice region
HA
LA
Mutations
Samples
B
missense_variant
synonymous_variant
frameshift_variant
stop_gained/stop_lost
splice_region_variant
others
LA
HA
100%
75%
50%
25%
0%
567805000
567806000
567807000
567808000
567809000
TraesCS3B02G357500.1
C
HA haplotype
LA haplotype
60
40
20
0
0
50
100
150

Supplementary Figure 11. Divergent haplotypes of *TaERF4* (TraesCS3B02G357500) in response to high-altitude environmental extremes shown in heatmap (A), lollipop graph (B) and HapMap chart (C). In heatmap plot (Panel A), first column from left indicates genomic regions of *TaERF4* gene and the second column from left indicates mutation types, whereas other columns indicate different wheat accessions; rows indicate SNP/InDel variations; In lollipop graph (Panel B), y-axis indicates mutation frequency in HA and LA wheat accessions; mutation types were distinguished by different colors. In HapMap graph (Panel C), geographic distribution of different haplotypes of *TaERF4* gene. The haplotype distribution analysis was determined by using 308 resequencing accessions and 1,026 wheat exome-capture samples. Orange indicates the proportion of HA adapted haplotype of *TaERF4* gene. HA, high-altitude wheat accessions, LA, low-altitude accessions. Geographic map in C was generated using R packages, including maps, ggmaps, ggplot2 and sf, with geographic information data edited from original version of the Natural Earth project database (version 2013). Source data are provided as a Source Data file.

```
AT5G15120.1             -MASLVYSPFTLSTSKAEHLSSLTNSTKHSFLRKKHRSTKPAKSFFKVKSAVSGNGLFTQ  59
TraesCS2A02G134000.1    -MSSLVSAPFATATGARKKAR--APAPLHSFLLAGRRGRRAAAA-TIRCAVA-GNGLFTQ  55
Zm00001d026603_T002     MSSSLVSTPFAAAAQ-KRLLA--APVPLHSFLLSGRRQPPRRAG-TIRCAVAGGNGLFTQ  56
                          :*** :**: ::   .     :    ****   :*      .     :.. *******

AT5G15120.1             TNPEVRRIVPIKRDNVPTVKIVYVVLEAQYQSSLSEAVQSLNKTS-RFASYEVVGYLVEE  118
TraesCS2A02G134000.1    TNPDVRRVVPA-ERGLPRVRVVYVVLEAQYQSSVTAAVMQLNADPRRAAEFEVVGYLVEE  114
Zm00001d026603_T002     TKPEVRRVVPSDPRGLPRVKVVYVVLEAQYQSSVTAAVQQLNADPRRAAAFEVVGYLVEE  116
                        *:*:***:**    .:* *::************:: ** .**    * * :*********

AT5G15120.1             LRDKNTYNNFCEDLKDANIFIGSLIFVEELAIKVKDAVEKERDRMDAVLVFPSMPEVMRL  178
TraesCS2A02G134000.1    LRDVDTYAAFCDDVAVANVFIGSLIFVEELALKVRDAVAAHRDRMDAVLVFPSMPEVMRL  174
Zm00001d026603_T002     LRDEDTYATFCADLADANVFIGSLIFVEELALKVKAAVEKERDRMDAVLVFPSMPEVMRL  176
                        *** :**  ** *:  **:************:**: **  .*******************

AT5G15120.1             NKLGSFSMSQLGQSKSPFFQLFKRKKQGSAGFADSMLKLVRTLPKVLKYLPSDKAQDARL  238
TraesCS2A02G134000.1    NKLGSFSMAQLGQSKSPFFQLFKRNKKDSSGFADSMLKLVRTLPKVLKYLPSDKAQDARL  234
Zm00001d026603_T002     NKLGSFSMSQLGQSKSPFFQLFKRNKANSSNFADSMLKLVRTLPKVLKYLPSDKAQDARL  236
                        ********:***************:* .*:.*****************************

AT5G15120.1             YILSLQFWLGGSPDNLQNFVKMISGSYVPALKGVKIEYSDPVLFLDTGIWHPLAPTMYDD  298
TraesCS2A02G134000.1    YILSLQFWLGGSPDNLQNFLKMIAVSYVPALKGADIKYSDPVLFLDTGIWHPLAPAMFDD  294
Zm00001d026603_T002     YILSLQFWLGGSPDNLQNFLKMIAGSYVPALKGAGIKYDDPVLYLDSGIWHPLAPTMYED  296
                        *******************:***: ********. *:*.****:**:********:*::*

AT5G15120.1             VKEYWNWYDTRRDTNDSLKRKDATVVGLVLQRSHIVTGDDSHYVAVIMELEARGAKVVPI  358
TraesCS2A02G134000.1    VKEYLNWYGTRRDANDRLKNPEAPVIGLVLQRSHIVTGDDGHYVAVIMELEARGAKVIPI  354
Zm00001d026603_T002     VKEYLNWYGTRRDANDRLKDPKAPIIGLVLQRSHIVTGDDGHYVAVIMELEAKGAKVIPI  356
                        **** ***.****:** **  .* ::**************.***********:****:**

AT5G15120.1             FAGGLDFSGPVEKYFVDPVSKQPIVNSAVSLTGFALVGGPARQDHPRAIEALKKLDVPYL  418
TraesCS2A02G134000.1    FAGGLDFSGPIERYLVDPITKKPFVNAVVSLTGFALVGGPARQDHPKAIASLMKLDVPYI  414
Zm00001d026603_T002     FAGGLDFSGPTQRYLVDPITGKTFVNAVVSLTGFALVGGPARQDHPKAIAALQKLDVPYI  416
                        ********** ::*:***:: : :**:.******************:** :* ******:

AT5G15120.1             VAVPLVFQTTEEWLNSTLGLHPIQVALQVALPELDGAMEPIVFAGRDPRTGKSHALHKRV  478
TraesCS2A02G134000.1    VALPLVFQTTEEWLNSTLGLHPIQVALQVALPELDGGMEPIVFAGRDPRSGKSHALHKRV  474
Zm00001d026603_T002     VALPLVFQTTEEWLNSTLGLHPIQVALQVALPELDG---------------KSHALHKRV  461
                        **:*********************************               *********

AT5G15120.1             EQLCIRAIRWGELKRKTKAEKKLAITVFSFPPDKGNVGTAAYLNVFASIFSVLRDLKRDG  538
TraesCS2A02G134000.1    EQLCTRAIRWAELKRKTKMDKKLAITVFSFPPDKGNVGTAAYLNVFSSIYSVLKDLKKDG  534
Zm00001d026603_T002     EQLCTRAIRWAELKRKTKEEKRLAITVFSFPPDKGNVGTAAYLNVFSSIYSVLSDLKKDG  521
                        **** *****.******* :*:************************:**:*** ***:**

AT5G15120.1             YNVEGLPENAETLIEEIIHDKEAQFSSPNLNVAYKMGVREYQDLTPYANALEENWGKPPG  598
TraesCS2A02G134000.1    YNVEGLPETPEELIEEVIHDKEAQFNSPNLNVVYRMNVREYQALTPYANMLEENWGKPPG  594
Zm00001d026603_T002     YNVEGLPDTPEALIEEVIHDKEAQFNSPNLNVAYRMNVREYQSLTSYASLLEENWGKPPG  581
                        *******:. * ****:********.******.*:*.***** ** **. **********

AT5G15120.1             NLNSDGENLLVYGKAYGNVFIGVQPTFGYEGDPMRLLFSKSASPHHGFAAYYSYVEKIFK  658
TraesCS2A02G134000.1    HLNSDGENLLVYGKQYGNIFIGVQPTFGYEGDPMRLLFSKSASPHHGFAAYYTFVEKIFK  654
Zm00001d026603_T002     HLNSDGENLLVYGKQYGNVFIGVQPTFGYEGDPMRLLFSKSASPHHGFAAYYTFVEKIFQ  641
                        :************* ***:*********************************::*****:

AT5G15120.1             ADAVLHFGTHGSLEFMPGKQVGMSDACFPDSLIGNIPNVYYYAANNPSEATIAKRRSYAN  718
TraesCS2A02G134000.1    ADAVLHFGTHGSLEFMPGKQVGMSDACFPDSLIGNIPNIYYYAANNPSEATVAKRRSYAN  714
Zm00001d026603_T002     ADAVLHFGTHGSLEFMPGKQVGMSDACFPDSLIGNIPNIYYYAANNPSEATVAKRRSYAN  701
                        **************************************:************:********

AT5G15120.1             TISYLTPPAENAGLYKGLKQLSELISSYQSLKDTGRGPQIVSSIISTAKQCNLDKDVDLP  778
TraesCS2A02G134000.1    TISYLTPPAENAGLYKGLKQLSELIASYQSLKDTGRGNQIVSSIISTAKQCNLDKDVDLP  774
Zm00001d026603_T002     TISYLTPPAENAGLYKGLKQLSELISSYQSLKDTGRGPQIVSSIVSTAKQCNLDKDVPLP  761
                        *************************:*********** ******:************ **

AT5G15120.1             DEGLELSPKDRDSVVGKVYSKIMEIESRLLPCGLHVIGEPPSAMEAVATLVNIAALDRPE  838
TraesCS2A02G134000.1    DEGEELPANERDLVVGKVYGKLMEIESRLLPCGLHVIGEPPTAVEAVATLVNIAALDRPE  834
Zm00001d026603_T002     EEGEELPPKERDLVVGKVYAKIMEIESRLLPCGLHVIGEPPSAIEAVATLVNIAALDRPE  821
                        :** **  ::** ******.*:*******************:*:****************

AT5G15120.1             DEISALPSILAECVGREIEDVYRGSDKGILSDVELLKEITDASRGAVSAFVEKTTNSKGQ  898
TraesCS2A02G134000.1    ENIFSLPGILAATVGRTIEDVYRGSDKGILADVELLKQITEASRGAVSAFVEKTTNSKGQ  894
Zm00001d026603_T002     DGITSLPGILAATVGRDIEDVYRGSDKGILADVELLRQITEASRGAITAFVEKTTNSKGQ  881
                        : * :**.***  *** *************:*****::**:*****::************
```

```
AT5G15120.1                 VVDVSDKLTSLLGFGINEPWVEYLSNTKFYRANRDKLRTVFGFLGECLKLVVMDNELGSL  958
TraesCS2A02G134000.1        VVDVTNKLSSILGFSLSEPWVEYLSQTKFIRADRDKLRTLFGFLGECLKLIVADNELGAL  954
Zm00001d026603_T002         VVNVANNLSKILGFGLSEPWVQYLSATKFVRADREKMRVLFGFLGECLRLVVQDNELGSL  941
                            **:*:::*:.:***.:.****:*** *** **:*:*:*.:********:*:* *****:*

AT5G15120.1                 MQALEGKYVEPGPGGDPIRNPKVLPTGKNIHALDPQAIPTTAAMASAKIVVERLVERQKL  1018
TraesCS2A02G134000.1        KTALEGSYVEPGPGGDPIRNPKVLPTGKNIHALDPQSIPTAAAMKSAKIVVERLLERQKA  1014
Zm00001d026603_T002         KLALEGSYVEPGPGGDPIRNPKVLPTGKNIHALDPQAIPTTAALKSAKIVVDRLLERQKA  1001
                              ****.*****************************:***:**: ******:**:****

AT5G15120.1                 ENEGKYPETIALVLWGTDNIKTYGESLGQVLWMIGVRPIADTFGRVNRVEPVSLEELGRP  1078
TraesCS2A02G134000.1        DNGGKYPETIALVLWGTDNIKTYGESLAQVMWMLGVEPVTDGLGRVNRVEPVSIEELGRP  1074
Zm00001d026603_T002         DNGGKYPETVALVLWGTDNIKTYGESLAQVLWMIGVRPVADTFGRVNRVEPVSLEELGRP  1061
                            :* ******:*****************.**:**:**.*::* :**********:******

AT5G15120.1                 RIDVVVNCSGVFRDLFINQMNLLDRAIKMVAELDEPVEQNFVRKHALEQAEALGIDIREA  1138
TraesCS2A02G134000.1        RIDVVVNCSGVFRDLFINQMNLLDRAVKMVAELDEPIEMNYVRKHAQEQAEELGVSVREA  1134
Zm00001d026603_T002         RIDVVVNCSGVFRDLFINQMNLLDRAVKMVAELDEPAEMNYVRKHAQEQAEELGVSLREA  1121
                            **************************:********* * *:***** **** **:.:***

AT5G15120.1                 ATRVFSNASGSYSANISLAVENSSWNDEKQLQDMYLSRKSFAFDSDAPGAGMAEKKQVFE  1198
TraesCS2A02G134000.1        ATRIFSNASGSYSSNVNLAVENASWTDEKQLQDMYLSRKSFAFDSDAPGVGMLEKRKTFE  1194
Zm00001d026603_T002         ATRVFSNASGSYSSNVNLAVENASWTDEKQLQDMYLSRKSFAFDSDAPGAGMKEKRKAFE  1181
                            ***:*********:*:.*****:**.***********************.** **::.**

AT5G15120.1                 MALSTAEVTFQNLDSSEISLTDVSHYFDSDPTNLVQSLRKDKKKPSSYIADTTTANAQVR  1258
TraesCS2A02G134000.1        LALATADATFQNLDSSEISLTDVSHYFDSDPTKLVQGLRKDGRAPSSYIADTTTANAQVR  1254
Zm00001d026603_T002         LALATADATFQNLDSSEISLTDVSHYFDSDPTKLVQGLRKDGRAPSSYIADTTTANAQVR  1241
                            :**:**:.************************:***.**** : ****************

AT5G15120.1                 TLSETVRLDARTKLLNPKWYEGMMSSGYEGVREIEKRLSNTVGWSATSGQVDNWVYEEAN  1318
TraesCS2A02G134000.1        TLSETVRLDARTKLLNPRWYEGMMKSGYEGVREIEKRLTNTVGWSATSGQVDNWVYEEAN  1314
Zm00001d026603_T002         TLSETVRLDARTKLLNPKWYEGMMKSGYEGVREIEKRLTNTVGWSATSGQVDNWVYEEAN  1301
                            *****************:******.*************:*********************

AT5G15120.1                 STFIQDEEMLNRLMNTNPNSFRKMLQTFLEANGRGYWDTSAENIEKLKELYSQVEDKIEG  1378
TraesCS2A02G134000.1        TTFIEDEEMRKRLMDTNPNSFRKLLQTFLEANGRGYWETSEDNLERLRELYSEVEDKIEG  1374
Zm00001d026603_T002         STFIEDEAMRKRLMDTNPNSFRKLVQTFLEASGRGYWETTEENLDRLRELYSEVEDKIEG  1361
                            :***:** * :***:********::******.*****:*: :*:::*:****:*******

AT5G15120.1                 IDR   1381
TraesCS2A02G134000.1        IDR   1377
Zm00001d026603_T002         IDR   1364
                            ***
```

Supplementary Figure 12. **Protein sequence alignment of TraesCS2A02G134000 (*TaCHLH*) and homologs of *CHLH* in *Arabidopsis thaliana* (AT5G15120.1), *Zea mays* (Zm00001d026603_T002).** Conserved domain, denoted by red lines, was predicted on NCBI Conserved Domain Database. Alignment was performed using EMBOSS Clustal Omega web server.

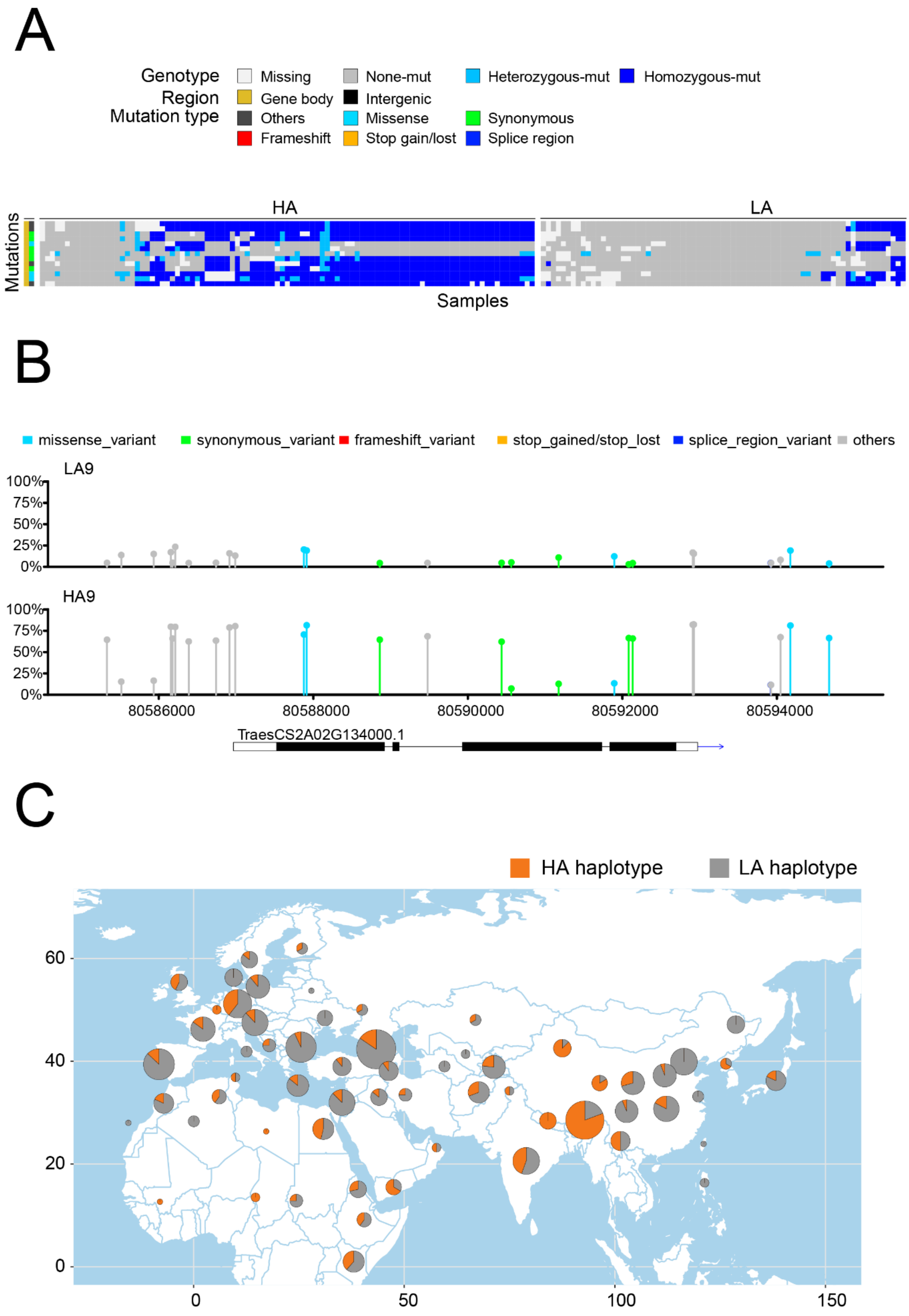
A
Genotype
Region
Mutation type
Missing
None-mut
Heterozygous-mut
Homozygous-mut
Gene body
Intergenic
Others
Missense
Synonymous
Frameshift
Stop gain/lost
Splice region
HA
LA
Mutations
Samples
B
missense_variant
synonymous_variant
frameshift_variant
stop_gained/stop_lost
splice_region_variant
others
LA9
HA9
100%
75%
50%
25%
0%
80586000
80588000
80590000
80592000
80594000
TraesCS2A02G134000.1
C
HA haplotype
LA haplotype
60
40
20
0
0
50
100
150

Supplementary Figure 13. Divergent haplotypes of *TaCHLH* (TraesCS2A02G134000) in response to high-altitude environmental extremes shown in heatmap (A), lollipop graph (B) and HapMap chart (C). In heatmap plot (Panel A), first column from left indicates genomic regions of *TaCHLH* gene and the second column from left indicates mutation types, whereas other columns indicate different wheat accessions; rows indicate SNP/InDel variations; In lollipop graph (Panel B), y-axis indicates mutation frequency in HA and LA wheat accessions; mutation types were distinguished by different colors. In HapMap graph (Panel C), geographic distribution of different haplotypes of *TaCHLH* gene. The haplotype distribution analysis was determined by using 308 resequencing accessions and 1,026 wheat exome-capture samples. Orange indicates the proportion of HA adapted haplotype of *TaCHLH* gene. HA, high-altitude wheat accessions, LA, low-altitude accessions. Geographic map in C was generated using R packages, including maps, ggmaps, ggplot2 and sf, with geographic information data edited from original version of the Natural Earth project database (version 2013). Source data are provided as a Source Data file.

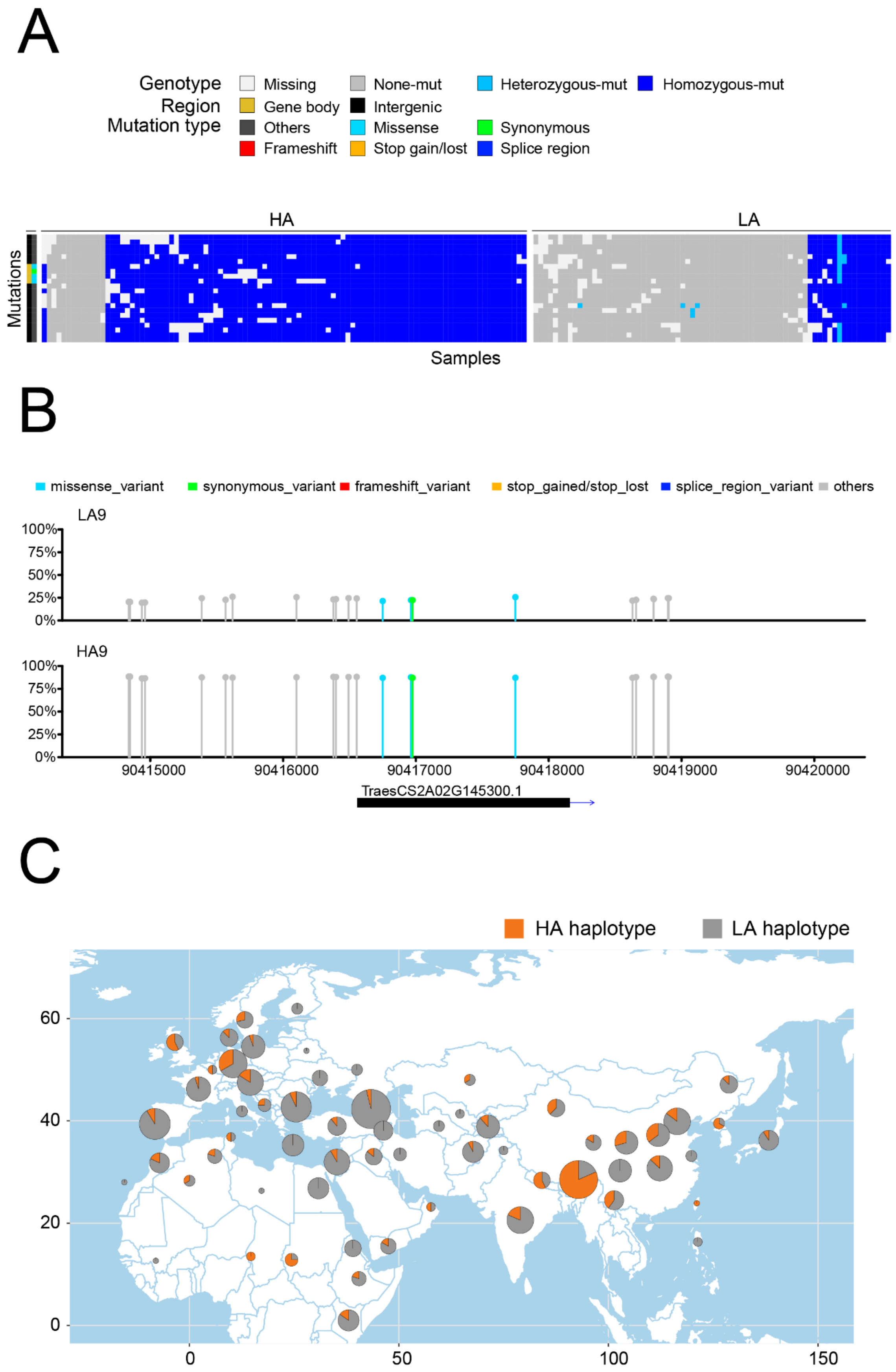
A
Genotype
Missing
None-mut
Heterozygous-mut
Homozygous-mut
Region
Gene body
Intergenic
Mutation type
Others
Missense
Synonymous
Frameshift
Stop gain/lost
Splice region
HA
LA
Mutations
Samples
B
missense_variant
synonymous_variant
frameshift_variant
stop_gained/stop_lost
splice_region_variant
others
LA9
100%
75%
50%
25%
0%
HA9
90415000
90416000
90417000
90418000
90419000
90420000
TraesCS2A02G145300.1
C
HA haplotype
LA haplotype
60
40
20
0
0
50
100
150

Supplementary Figure 14. Divergent haplotypes of TraesCS2A02G145300 in response to high-altitude environmental extremes shown in heatmap (A), lollipop graph (B) and HapMap chart (C). In heatmap plot (Panel A), first column from left indicates genomic regions of TraesCS2A02G14530 gene and the second column from left indicates mutation types, whereas other columns indicate different wheat accessions; rows indicate SNP/InDel variations; In lollipop graph (Panel B), y-axis indicates mutation frequency in HA and LA wheat accessions; mutation types were distinguished by different colors. In HapMap graph (Panel C), geographic distribution of different haplotypes of TraesCS2A02G14530 gene. The haplotype distribution analysis was determined by using 308 resequencing accessions and 1,026 wheat exome-capture samples. Orange indicates the proportion of HA adapted haplotype of TraesCS2A02G14530 gene. HA, high-altitude wheat accessions, LA, low-altitude accessions. Geographic map in C was generated using R packages, including maps, ggmaps, ggplot2 and sf, with geographic information data edited from original version of the Natural Earth project database (version 2013). Source data are provided as a Source Data file.

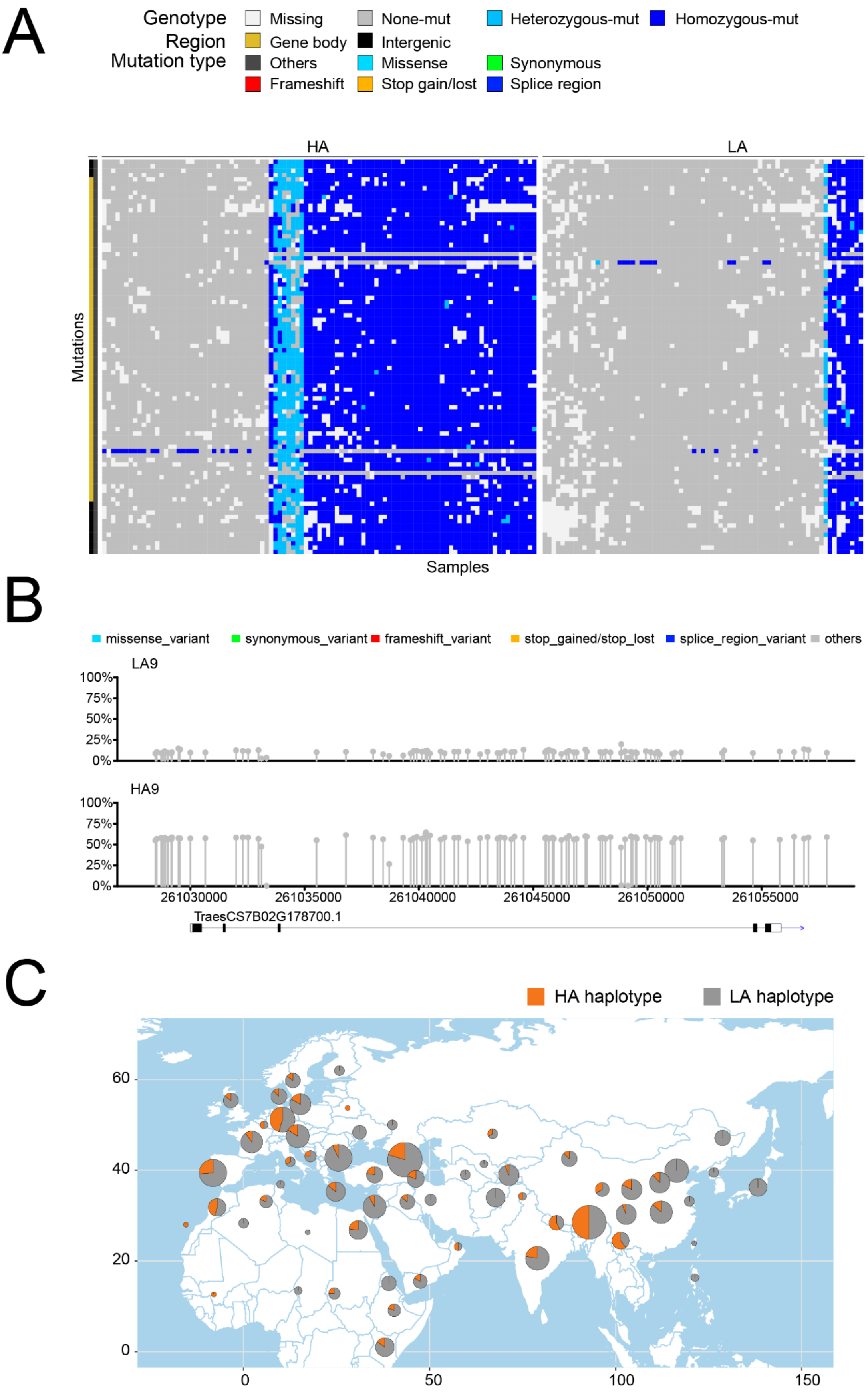

A
Genotype
Region
Mutation type
Missing
None-mut
Heterozygous-mut
Homozygous-mut
Gene body
Intergenic
Others
Missense
Synonymous
Frameshift
Stop gain/lost
Splice region
HA
LA
Mutations
Samples
B
missense_variant
synonymous_variant
frameshift_variant
stop_gained/stop_lost
splice_region_variant
others
LA9
HA9
100%
75%
50%
25%
0%
261030000
261035000
261040000
261045000
261050000
261055000
TraesCS7B02G178700.1
C
HA haplotype
LA haplotype
60
40
20
0
0
50
100
150

Supplementary Figure 15. Divergent haplotypes of TraesCS7B02G178700 in response to high-altitude environmental extremes shown in heatmap (A), lollipop graph (B) and HapMap chart (C). In heatmap plot (Panel A), first column from left indicates genomic regions of TraesCS7B02G178700 gene and the second column from left indicates mutation types, whereas other columns indicate different wheat accessions; rows indicate SNP/InDel variations; In lollipop graph (Panel B), y-axis indicates mutation frequency in HA and LA wheat accessions; mutation types were distinguished by different colors. In HapMap graph (Panel C), geographic distribution of different haplotypes of TraesCS7B02G178700 gene. The haplotype distribution analysis was determined by using 308 resequencing accessions and 1,026 wheat exome-capture samples. Orange indicates the proportion of HA adapted haplotype of TraesCS7B02G178700 gene. HA, high-altitude wheat accessions, LA, low-altitude accessions. Geographic map in C was generated using R packages, including maps, ggmaps, ggplot2 and sf, with geographic information data edited from original version of the Natural Earth project database (version 2013). Source data are provided as a Source Data file.

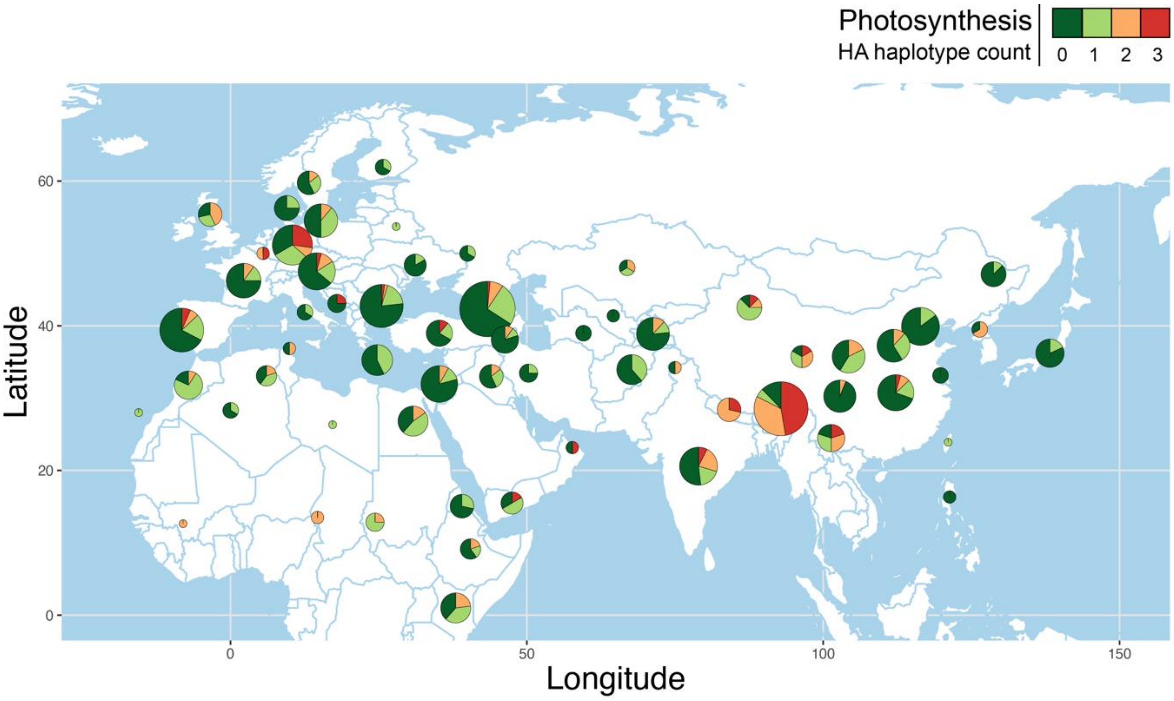

Supplementary Figure 16. Haplotype distributions of photosynthesis related genes (*TaCHLH*, *TraesCS2A02G145300*, *TraesCS7B02G178700*) according to geography for divergent genes between HA and LA wheat groups. The haplotype distribution analysis was determined by using 308 resequencing accessions and 1,026 wheat exome-capture samples. The numbers (0~3) represent the pyramiding amount of HA adapted haplotype indicated by different colors. The pie program showed haplotype distribution of divergent genes related to photosynthesis. Geographic map was generated using R packages, including maps, ggmaps, ggplot2 and sf, with geographic information data edited from original version of the Natural Earth project database (version 2013).

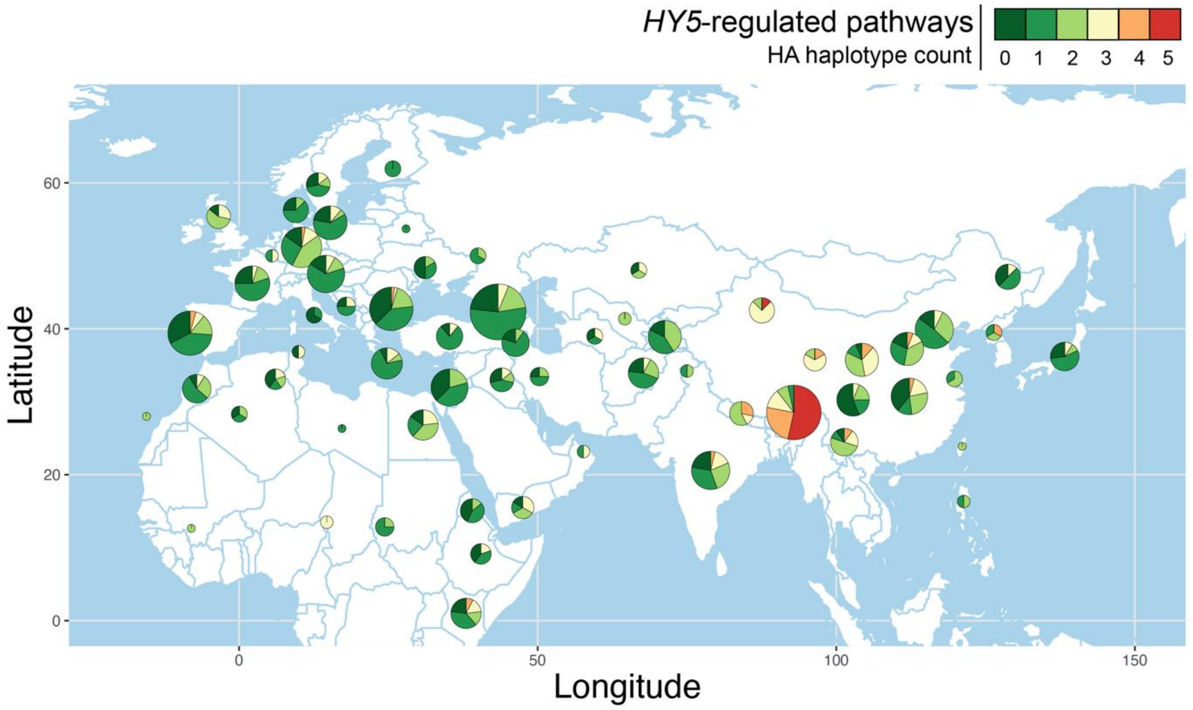

Supplementary Figure 17. Haplotype distributions of *HY5*-regulated pathways (including *TaHY5-like*, *TaERF4*, *TaPCO1*, *TaCHLH* and *TaTDP1*) according to geography for divergent genes between HA and LA wheat groups. The haplotype distribution analysis was determined by using 308 resequencing accessions and 1,026 wheat exome-capture samples. The numbers (0~3) represent the pyramiding amount of HA adapted haplotype indicated by different colors. The pie program showed haplotype distribution of divergent genes related to *HY5*-regulated pathways. Geographic map was generated using R packages, including maps, ggmaps, ggplot2 and sf, with geographic information data edited from original version of the Natural Earth project database (version 2013).

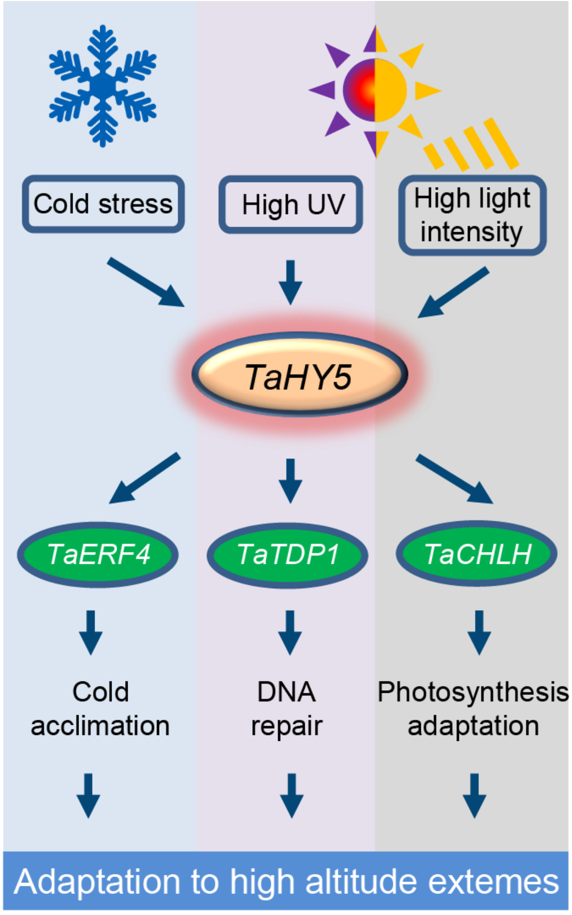

Supplementary Figure 18. The proposed model of *TaHY5-like* mediated signaling pathway to adapt to high-altitude extremes in Tibetan wheat. The snowflake icon was downloaded from freepik.com (under Freepik License) with modification.

```
TraesCS2A02G081900.1    ---------------------MRPRPPPP--SAMDRHHHHHQQQQQQPPSPQEEHAAQPR  37
TraesCS2D02G079600.1    ---------------------------------MDRHH------HQQPPSPQGEHAAQPR  21
AT4G15510.1             MASSAFAFPSYIITKGASTDSFKSTSLSSSRSLVTDFHLLFSRPISSGPKYQSAKSAKPE  60
OIT29357                -MRILQASTSYSVGFGISSISTRPKSFTQRHSTIAE-GQALDKPVQTTRKFSEQHTLKKG  58
                                                         :           .   . .  :: :

TraesCS2A02G081900.1    CWEEFLHRKTIRVLLVETDDSTRQVVTALLRHCMYQVIPAENGHQAWAYLQDMQSNIDLV  97
TraesCS2D02G079600.1    CWEEFLHRKTIRVLLVETDDSTRQVVTALLRHCMYQVIPAENGHQAWAYLQDMQSNIDLV  81
AT4G15510.1             SPVA-------------------------I-NCLT-------------------------  69
OIT29357                DVVK-------------------------EGTSVV-------------------------  68
                                                        .:

TraesCS2A02G081900.1    LTEVFMHGGLSGIDLLGRIMNHEVCKDIPVIMMSSHDSMGTVLSCLSNGA-----ADFLA  152
TraesCS2D02G079600.1    LTEVFMHGGLSGIDLLGRIMNHEVCKDIPVIMMSSHDSMGTVLSCLSNGA-----ADFLA  136
AT4G15510.1             -------------------DAKQVCAV----GRRKSMMMGLLMSGLIVSQANLP-TAFAS  105
OIT29357                -------------------LEKKDFAV----PRRK--AMALIFSSLVLSNYELPKVAYAQ  103
                                             ::           .   *. ::* *  .      . :

TraesCS2A02G081900.1    KPIRK------NELKNLWAHVWRRSHSSSGSGSGSAIQTQKCTKSKSGDDSNNNSNNRND  206
TraesCS2D02G079600.1    KPIRK------NELKNLWAHVWRRSHSSSGSGSGSAIQTQKCTKSKSGDDSNNNSNNRND  190
AT4G15510.1             TPVFREYIDTFDGYSFKYPQNWIQVR-----GAGADIFFRDPVV-------------LDE  147
OIT29357                STEFREYIDTFDGYSFLYPRNWIQVR-----GAGADIFFRDPLV-------------LDE  145
                        .   :      :  .  : : * : :     *:*: *  :.                 ::

TraesCS2A02G081900.1    DASMGLNARDGSDNGSGT--QSSWTKRAVEIDSPQD--MSPDQSIDPPDSTCAHV-SHLK  261
TraesCS2D02G079600.1    DASMGLNARDGSDNGSGTQAQSSWTKRAVEIDSPQD--MSPDQSVDPPDSTCAHV-SHLK  247
AT4G15510.1             NLS-------------------------VEFSSPSSSNYTSLEDLGSPEEVGKRVLRQYL  182
OIT29357                NLS-------------------------MNISSPSSSAYKSVEDLGPPEKAGKGVLRQYL  180
                        : *                         :::.**..   .  :.:. *:..   *  :

TraesCS2A02G081900.1    SEICSNRLRGDEFKGKELEIGAPGNLNTDDQSSPNESSVKPT-DGRCEYLPQNNSNDTVM  320
TraesCS2D02G079600.1    SEICSNRLRA-EFKGKELEIGAPGNLNTEDQSSPNESSVKPADNGRCEYLPQNNSNDTVM  306
AT4G15510.1             T----------EFMSTRLGV--------KRQANILSTSSRVADDGKLYYQVEVNIKSY--  222
OIT29357                T----------EFMSTRLGV--------RRESSILSTSSRVADDGKLYYEVEVNIKSY--  220
                        :          ** ...* :          ::.  .:* : : :*:  *  : * :.

TraesCS2A02G081900.1    ENSDEPIVRAADLIGSMAKNMDAQQA--ARAIDAPNCSSQVPEGKDADRENAMPYLELSL  378
TraesCS2D02G079600.1    ENSDEPIVRAADLIGSMAKNMDAQQA--ARAIDAPNCSSQAPQGKDTDRENAMPYLELSL  364
AT4G15510.1             ANNNELAVMPQDRVARLEWNRRYLAVLGVENDRLYSIRLQTPEKVFLEEEKDLRRV----  278
OIT29357                ANNNELAVMPQDRVARLEWDRRYLSVLGVENNRLYELRIQTPEKTFADEESDIRAI----  276
                         *.:*  *   * :. :  :     .  ..     .   *.*:    :.*. :  :

TraesCS2A02G081900.1    KRSRSTADDADAAIQEEQRNVVRRSDLSAFTRYNTCAVSNQGRAGFVGSCSPNGNSSEAA  438
TraesCS2D02G079600.1    KRSRSTADGADAAIQEEQRNVVRRSDLSAFTRYNTCAVSNQGGAGFVGSCSPNGNSSEAA  424
AT4G15510.1             ------------------------MD--SF-RVEKI------------------------  287
OIT29357                ------------------------MD--SF-RVNKIEV----------------------  287
                                                 *  :* * :.

TraesCS2A02G081900.1    KTDAAQMKQGSNGSSNNNDMGSTTKSVVTKPAGGNNKVSPINGNTHTSAFHRVQPWTPAT  498
TraesCS2D02G079600.1    KTDAAQMKQGSNGSSNNNDMGSTTKSVVTKPAGGNNKVSPINGNTHTSAFHRVQPWTPAT  484
AT4G15510.1             ------------------------------------------------------------  287
OIT29357                ------------------------------------------------------------  287

TraesCS2A02G081900.1    AAGKDKADETSKKNAATAAAAAKDMSGEAQSKHPCAAAHDANGGS-AGGTAQSSLVNPSG  557
TraesCS2D02G079600.1    AAGKDKADEMSKKNAATAA-AAKDNGGEAQSKRPCAAAHDTNGGSSAGGTAQSNVVNPSG  543
AT4G15510.1             ------------------------------------------------------------  287
OIT29357                ------------------------------------------------------------  287

TraesCS2A02G081900.1    PVEGHAANYGSNSGSNNNTNNGSTAATAAGAAAAVHAETGGIDKRSNMMHMKRERRVAAV  617
TraesCS2D02G079600.1    PVEGHAANYGSNSGSNNNTNNGSTAAT---AAAAVHAETGGIDKRSNMMHMKRERRVAAV  600
AT4G15510.1             ------------------------------------------------------------  287
OIT29357                ------------------------------------------------------------  287

TraesCS2A02G081900.1    NKFREKRKERNFGKKVRYQSRKRLAEQRPRVRGQFVRQPPPPAAVER-      664
TraesCS2D02G079600.1    NKFREKRKERNFGKKVRYQSRKRLAEQRPRVRGQFVRLPLRDNLPPHT      648
AT4G15510.1             ------------------------------------------------      287
OIT29357                ------------------------------------------------      287
```

Supplementary Figure 19. Protein sequence alignment of two wheat *PPD1* homologs (*TaPPD1-2A*, TraesCS2A02G081900; *TaPPD1-2D*, TraesCS2D02G079600) and homologs of *PPD1* in *Arabidopsis thaliana* (AT4G15510.1), *Nicotiana attenuate* (OIT29357). Conserved domain, denoted by red lines, was predicted on NCBI Conserved Domain Database. Alignment was performed using EMBOSS Clustal Omega web server.

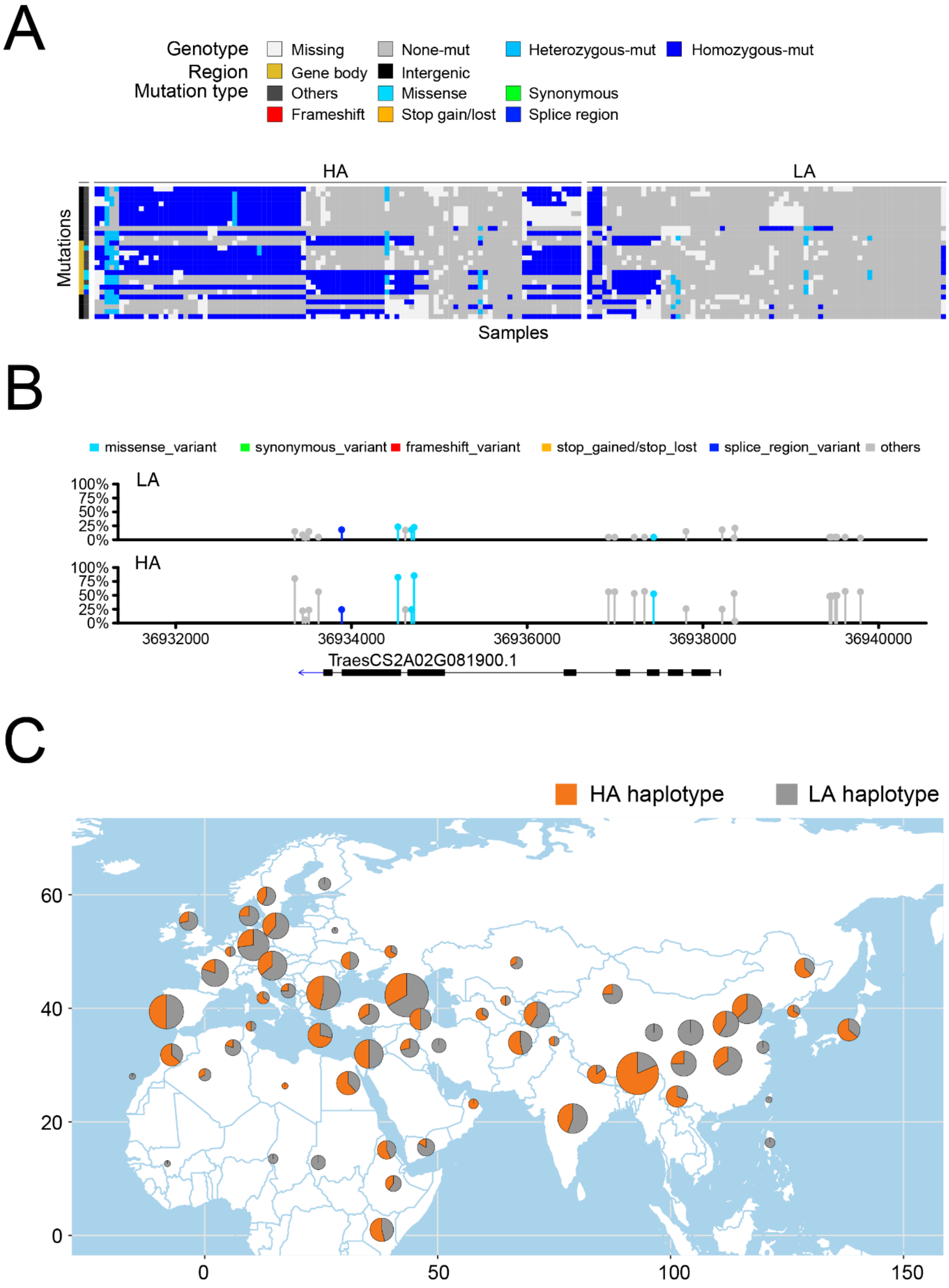
A
Genotype
Missing
None-mut
Heterozygous-mut
Homozygous-mut
Region
Gene body
Intergenic
Mutation type
Others
Missense
Synonymous
Frameshift
Stop gain/lost
Splice region
HA
LA
Mutations
Samples
B
missense_variant
synonymous_variant
frameshift_variant
stop_gained/stop_lost
splice_region_variant
others
LA
HA
100%
75%
50%
25%
0%
36932000
36934000
36936000
36938000
36940000
TraesCS2A02G081900.1
C
HA haplotype
LA haplotype
60
40
20
0
0
50
100
150

Supplementary Figure 20. Divergent haplotypes of *TaPPD1-2A* (TraesCS2A02G081900) in response to high-altitude environmental extremes shown in heatmap (A), lollipop graph (B) and HapMap chart (C). In heatmap plot (Panel A), first column from left indicates genomic regions of *TaPPD1-2A* gene and the second column from left indicates mutation types, whereas other columns indicate different wheat accessions; rows indicate SNP/InDel variations; In lollipop graph (Panel B), y-axis indicates mutation frequency in HA and LA wheat accessions; mutation types were distinguished by different colors. In HapMap graph (Panel C), geographic distribution of different haplotypes of *TaPPD1-2A* gene. The haplotype distribution analysis was determined by using 308 resequencing accessions and 1,026 wheat exome-capture samples. Orange indicates the proportion of HA adapted haplotype of *TaPPD1-2A* gene. HA, high-altitude wheat accessions, LA, low-altitude accessions. Geographic map in C was generated using R packages, including maps, ggmaps, ggplot2 and sf, with geographic information data edited from original version of the Natural Earth project database (version 2013). Source data are provided as a Source Data file.

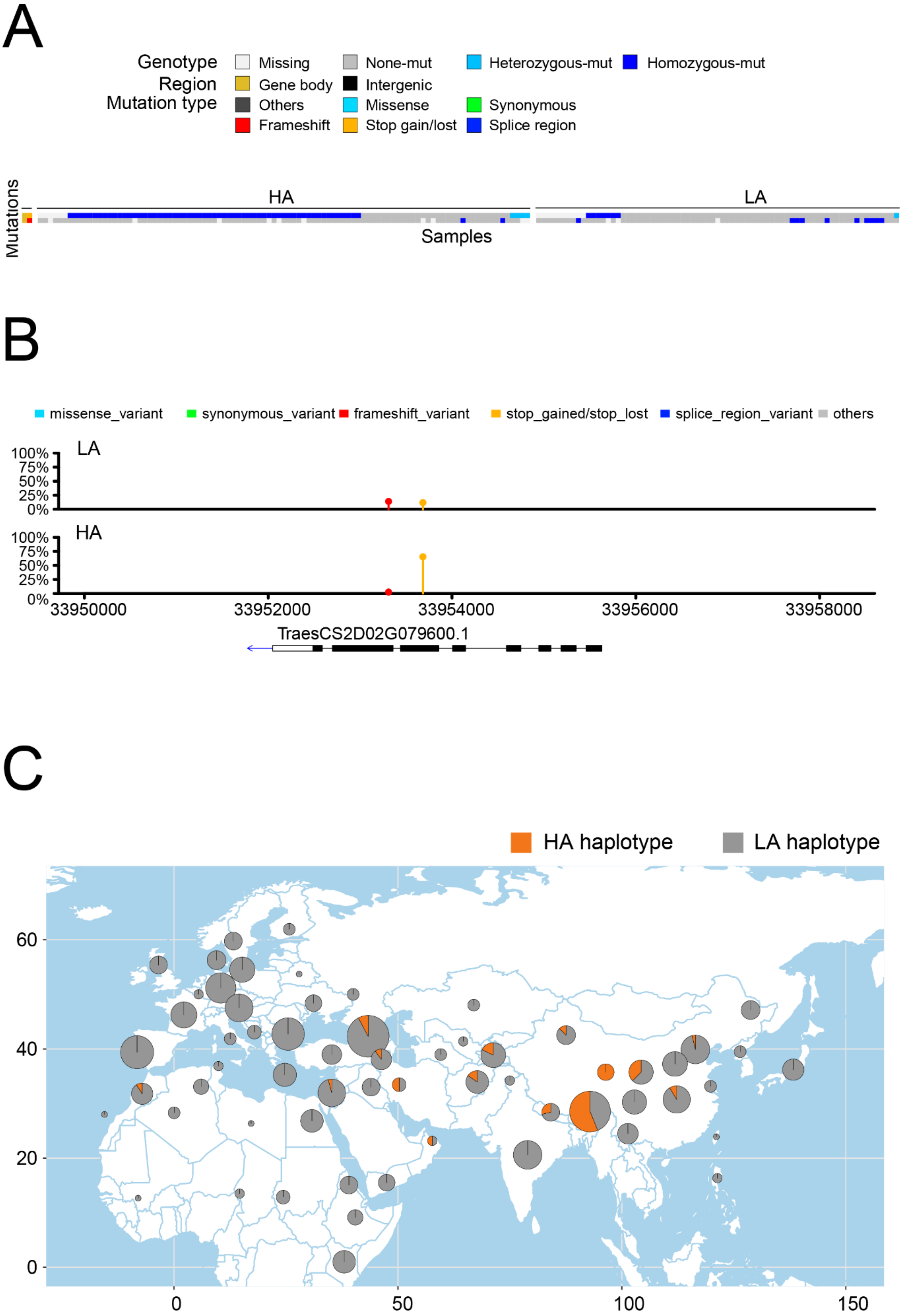
A
Genotype
Missing
None-mut
Heterozygous-mut
Homozygous-mut
Region
Gene body
Intergenic
Mutation type
Others
Missense
Synonymous
Frameshift
Stop gain/lost
Splice region
Mutations
HA
LA
Samples
B
missense_variant
synonymous_variant
frameshift_variant
stop_gained/stop_lost
splice_region_variant
others
LA
HA
100%
75%
50%
25%
0%
33950000
33952000
33954000
33956000
33958000
TraesCS2D02G079600.1
C
HA haplotype
LA haplotype
60
40
20
0
0
50
100
150

Supplementary Figure 21. Divergent haplotypes of *TaPPD1-2D* (TraesCS2D02G079600) in response to high-altitude environmental extremes shown in heatmap (A), lollipop graph (B) and HapMap chart (C). In heatmap plot (Panel A), first column from left indicates genomic regions of *TaPPD1-2D* gene and the second column from left indicates mutation types, whereas other columns indicate different wheat accessions; rows indicate SNP/InDel variations; In lollipop graph (Panel B), y-axis indicates mutation frequency in HA and LA wheat accessions; mutation types were distinguished by different colors. In HapMap graph (Panel C), geographic distribution of different haplotypes of *TaPPD1-2D* gene. The haplotype distribution analysis was determined by using 308 resequencing accessions and 1,026 wheat exome-capture samples. Orange indicates the proportion of HA adapted haplotype of *TaPPD1-2D* gene. HA, high-altitude wheat accessions, LA, low-altitude accessions. Geographic map in C was generated using R packages, including maps, ggmaps, ggplot2 and sf, with geographic information data edited from original version of the Natural Earth project database (version 2013). Source data are provided as a Source Data file.

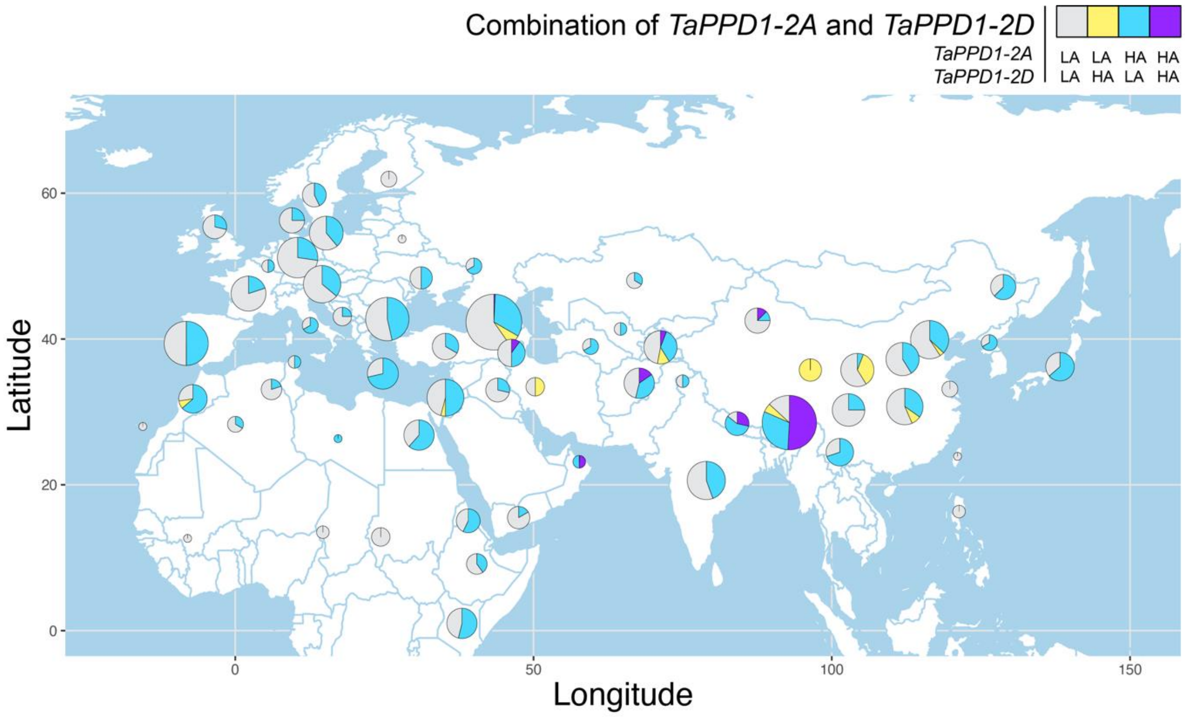

Supplementary Figure 22. Haplotype distributions of the combination of *TaPPD1-2A* (*TraesCS2A02G081900*) and *TaPPD1-2D* (*TraesCS2D02G079600*) according to geography for divergent genes between HA and LA wheat groups. The haplotype distribution analysis was determined by using 308 resequencing accessions and 1,026 wheat exome-capture samples. The numbers (0~3) represent the pyramiding amount of HA adapted haplotype indicated by different colors. The pie program showed haplotype distribution of *TaPPD1-2A* and *TaPPD1-2D* homeologs in response to high-altitude extremes. Geographic map was generated using R packages, including maps, ggmaps, ggplot2 and sf, with geographic information data edited from original version of the Natural Earth project database (version 2013).

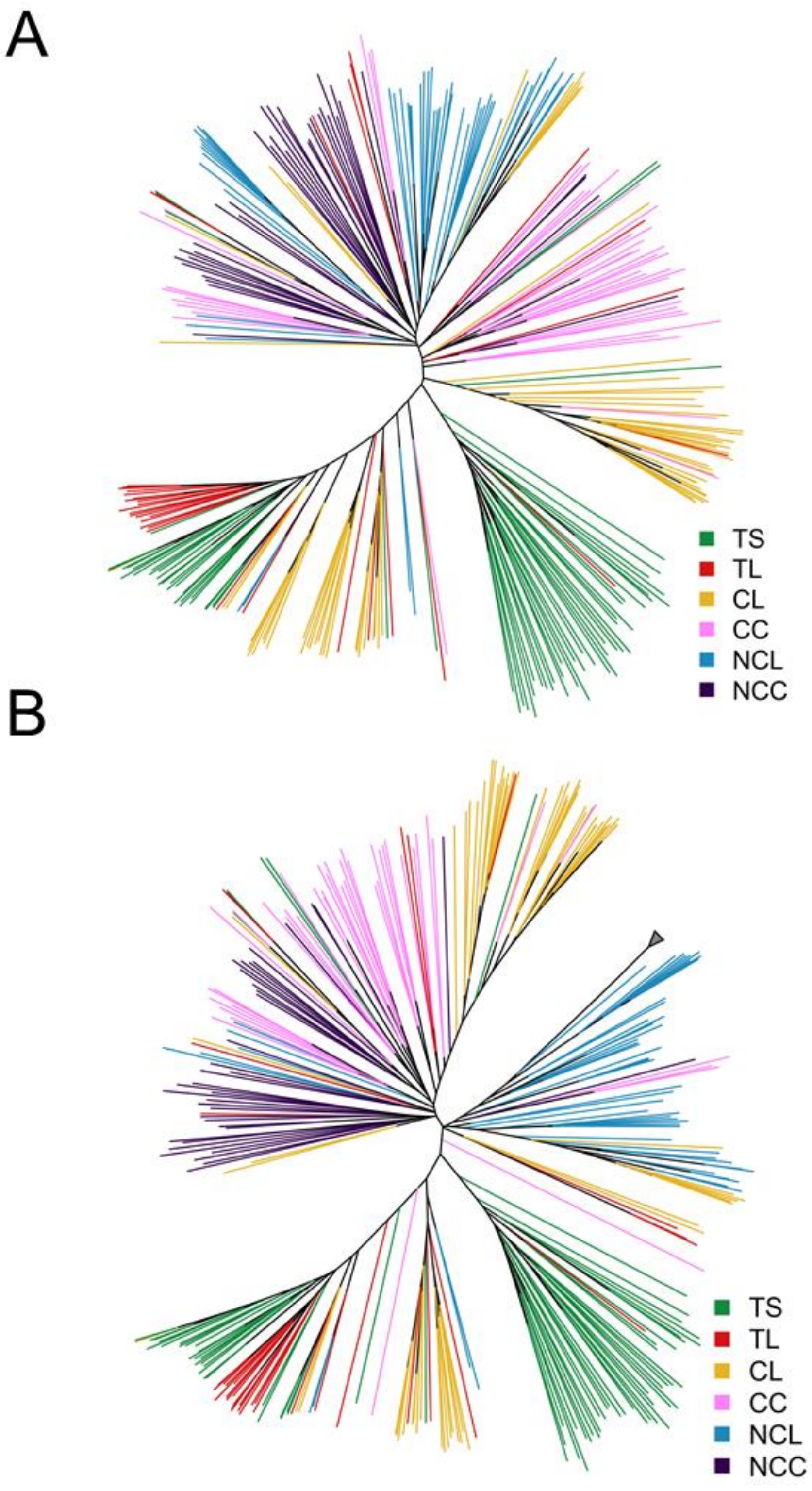

Supplementary Figure 23. Phylogenetic analysis of wheat resequencing samples using high-confidence 36,667,563 SNPs in whole genome (A) and 33,333,627 SNPs in A&B subgenomes. The gray triangle indicates outgroup of wild emmer (wild progenitors for tetraploid wheat, AABB) accessions (B). NCL, Non-Chinese landrace; NCC, Non-Chinese cultivar; CL, Chinese landrace (excluding Tibetan landrace); CC, Chinese cultivar; TS, Tibetan semi-wild wheat; TL, Tibetan landrace. Source data are provided as a Source Data file.

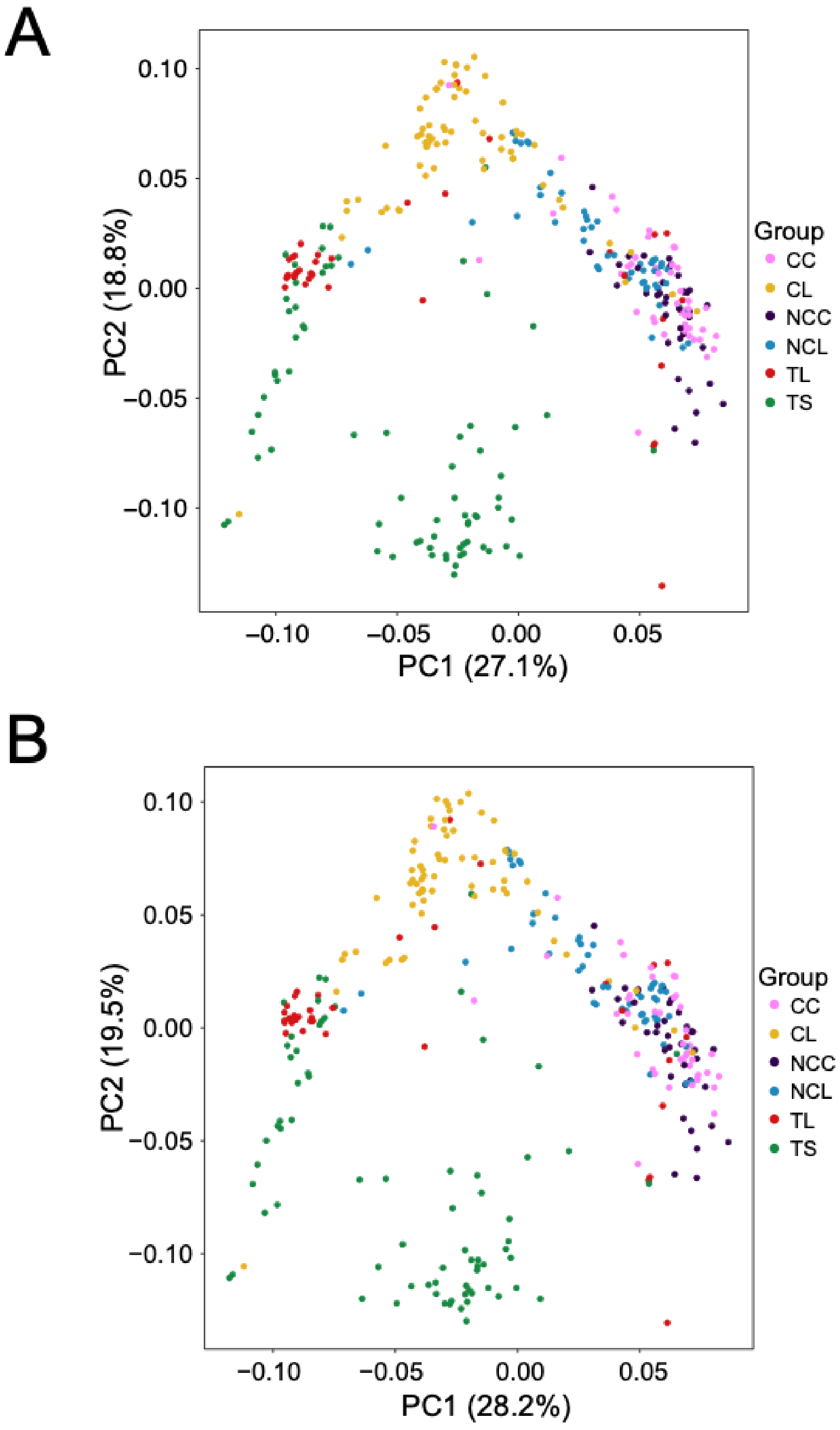

Supplementary Figure 24. PCA plots of all re-sequenced samples based on SNPs in whole genome (A) and in A&B subgenomes (B). NCL, Non-Chinese landrace; NCC, Non-Chinese cultivar; CL, Chinese landrace (excluding Tibetan landrace); CC, Chinese cultivar; TS, Tibetan semi-wild wheat; TL, Tibetan landrace. Source data are provided as a Source Data file.

A

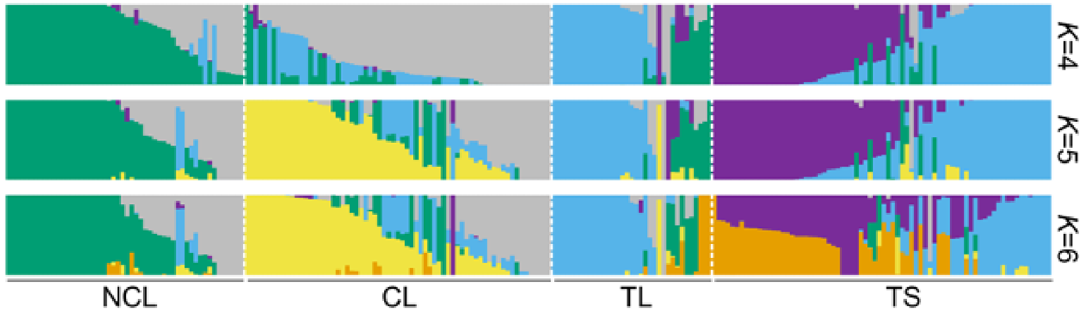

B

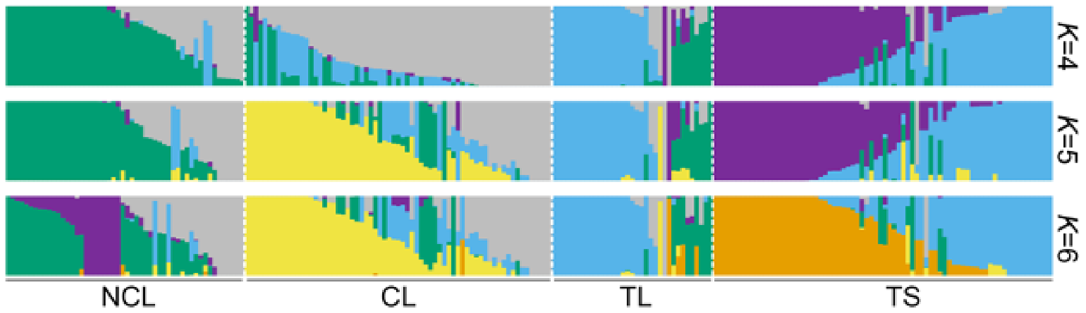

Supplementary Figure 25. Individual ancestry coefficients of resequencing accessions inferred using ADMIXTURE *K*=4, 5 and 6 based on SNPs in whole genome (A) and in A&B subgenomes (B). Each color represents one population. The length of segment in each vertical bar represents the proportion contributed by ancestral populations. Source data are provided as a Source Data file.

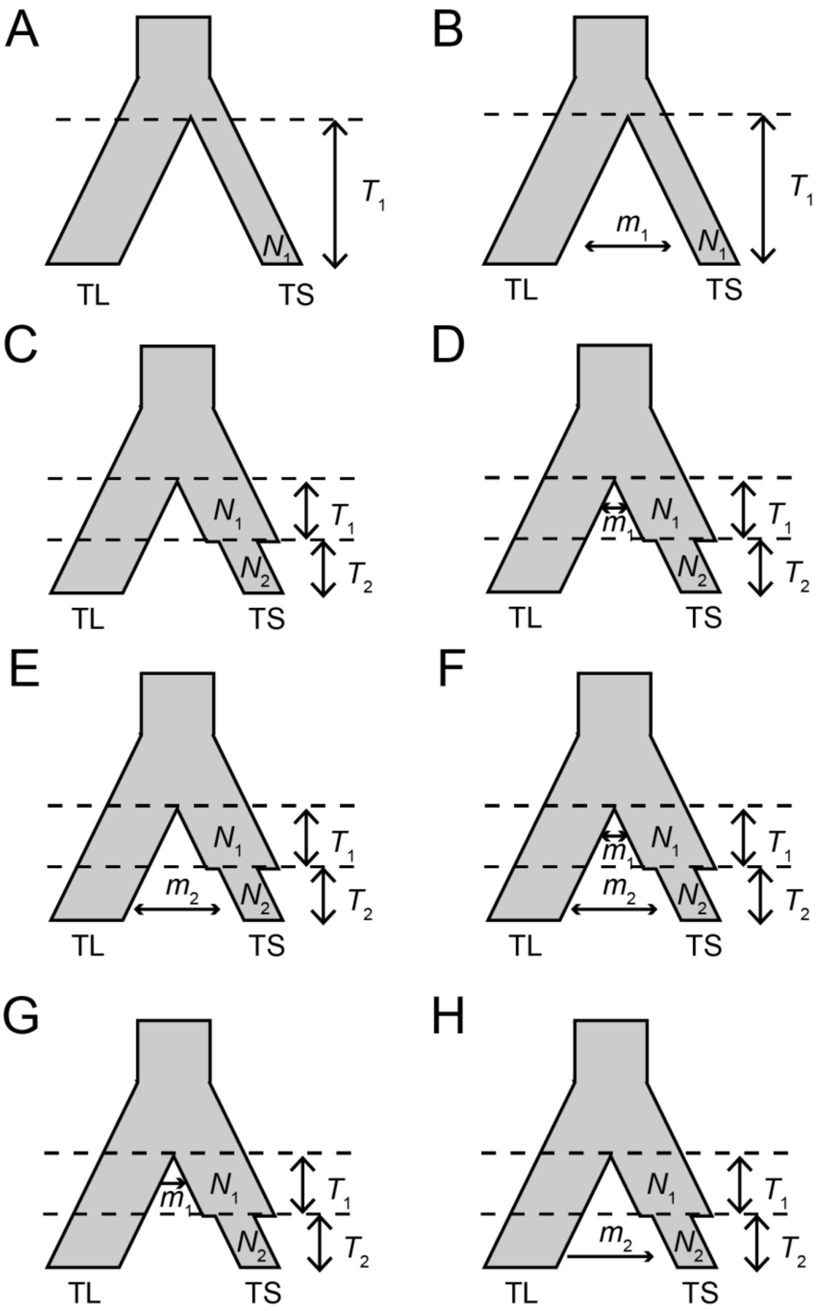

Supplementary Figure 26. Eight demographic models considered in the demographic analysis on the origin of the Tibetan semi-wild wheat. A. Model assumes that the initiate Tibetan semi-wild wheat (TS) split from Tibetan landrace (TL) without bottleneck and not allow migration. B. Model assumes that the initiate TS split from TL without bottleneck and allow migration. C. Model assumes that the initiate TS split from TL with bottleneck and not allow migration. D. Model assumes that the initiate TS split from TL with bottleneck and allow migration. E. Model assumes that the initiate TS split from TL with bottleneck and allow migration after bottleneck. F. Model assumes that the initiate TS split from TL with bottleneck and allow migration during and after bottleneck. G. Model assumes that the initiate TS split from TL with bottleneck and allow migration from TL to TS during bottleneck. H. Model assumes that the initiate TS split from Tibetan landrace with bottleneck and allow migration from TL to TS after bottleneck.

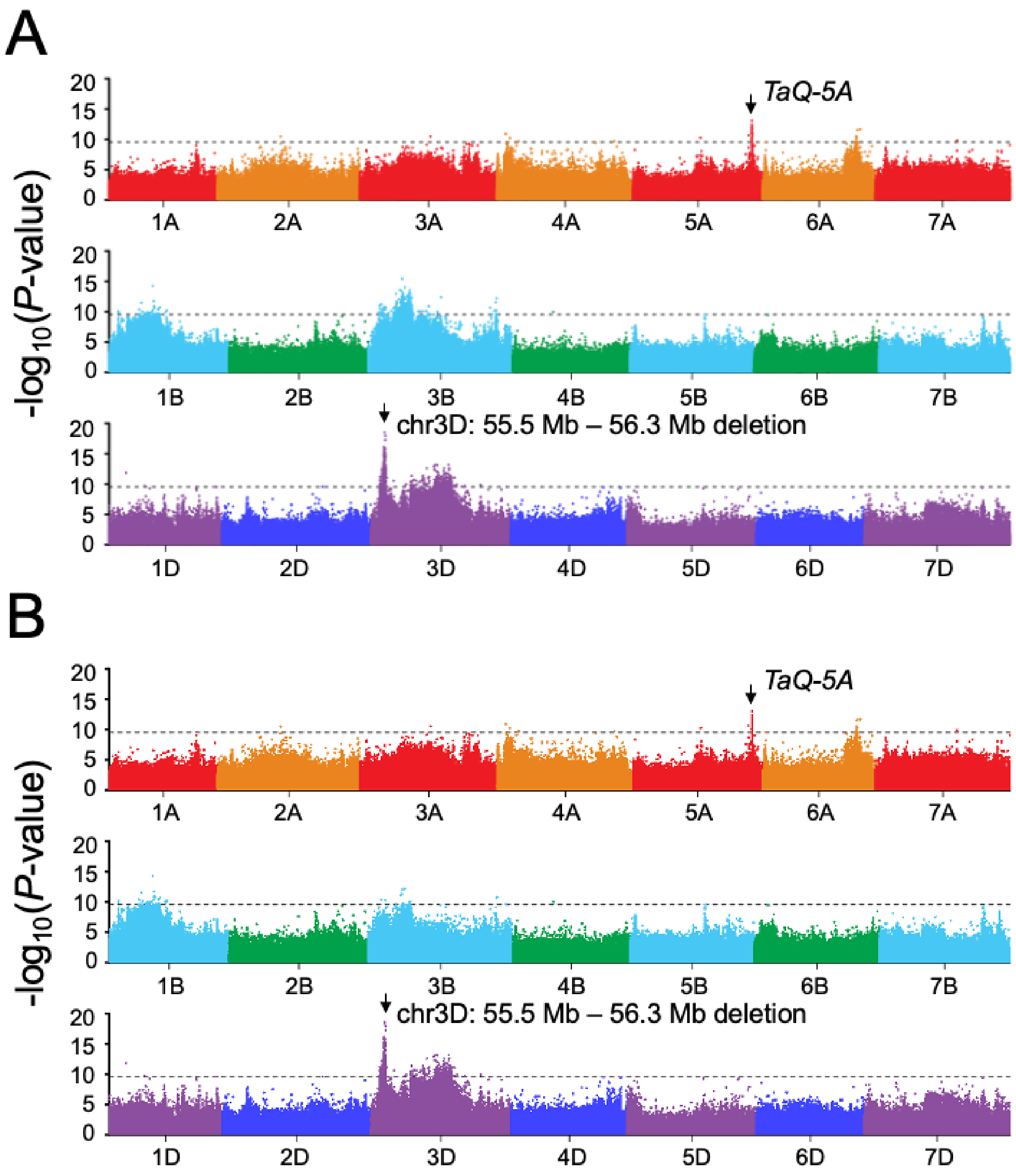

Supplementary Figure 27. Genome-wide scanning of candidate regions for rachis brittleness during wheat de-domestication process using GWAS analysis. Dashed lines represent the Bonferroni adjusted *P*-value at the level of 0.01 (A). For chr3B, the combined top five PCs of both whole genome and chr3B were used to estimate population structure. The detailed method for chr3B analysis is stated in method (B). Source data are provided as a Source Data file.

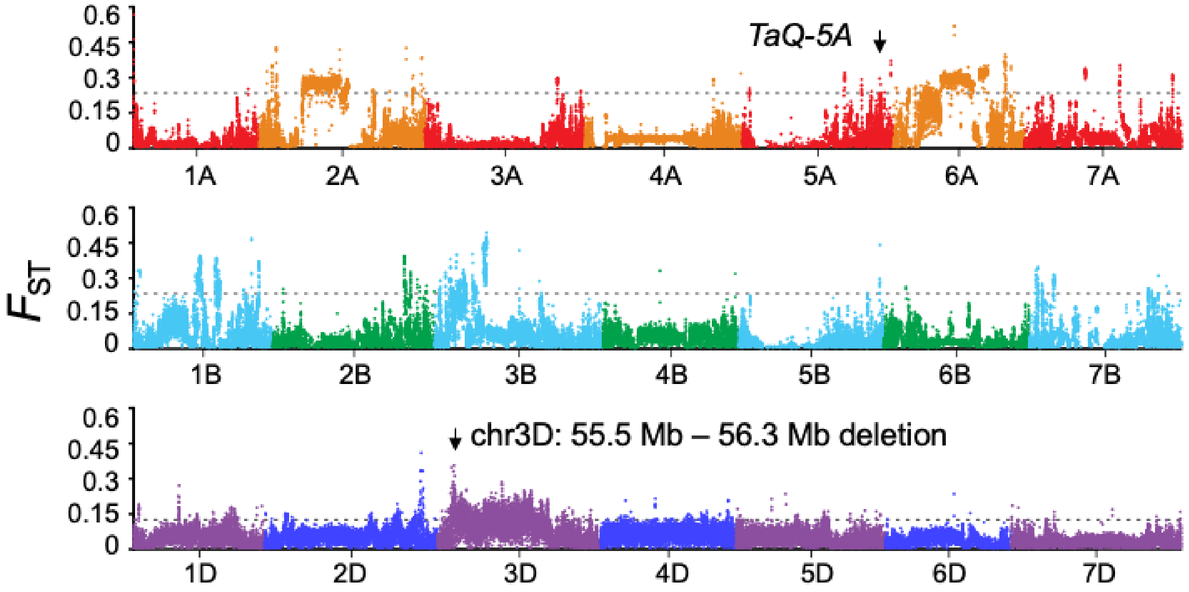

Supplementary Figure 28. Genomic differentiation during de-domestication process of Tibetan semi-wild wheat revealed by F_{ST}. Dashed lines represent the top 5% thresholds for A&B subgenome (as a whole) and D subgenome, respectively. The genomic position of 0.8 Mb deleted region and *TaQ-5A* gene were labeled with arrows. Source data are provided as a Source Data file.

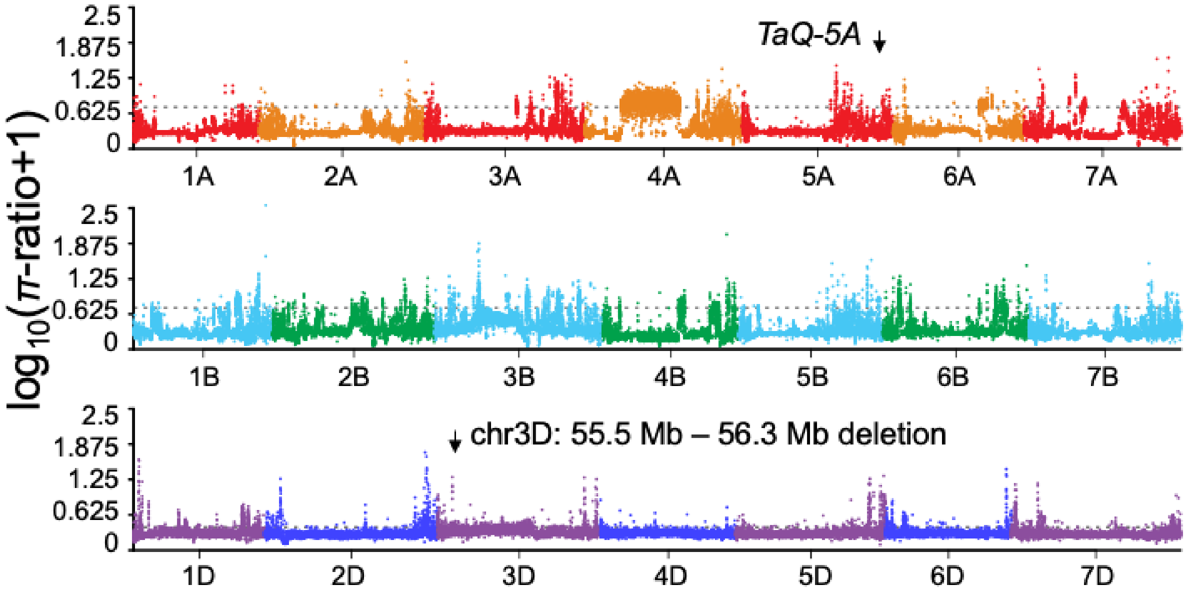

Supplementary Figure 29. Genomic differentiation during de-domestication process of Tibetan semi-wild wheat revealed by π-ratio. Y-axis, π-ratio=$\log_{10}(\pi_{DO}/\pi_{DE}+1)$. Dashed lines represent the top 5% thresholds for A&B subgenome (as a whole) and D subgenome, respectively. The genomic positions of 0.8 Mb deleted region and *TaQ-5A* gene were labeled with arrows. DE: de-domesticated wheat accessions; DO: domesticated wheat accessions. Source data are provided as a Source Data file.

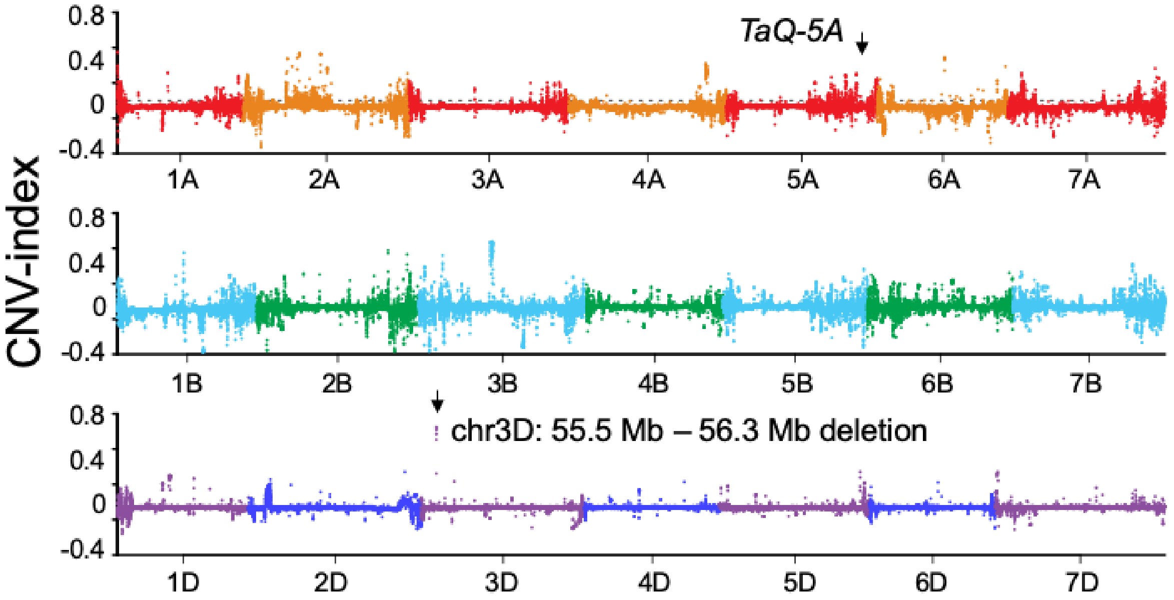

Supplementary Figure 30. Genomic differentiation during de-domestication process of Tibetan semi-wild wheat revealed by CNV-index. Y-axis, CNV-index values (NCN_{DO}-NCN_{DE}) for inter-group coverage depth. NCN_{DO} and NCN_{DE} represent normalized read coverage for DO and DE wheat accessions, respectively. Dashed lines represent the top 5% thresholds for A&B subgenome (as a whole) and D subgenome, respectively. The genomic positions of 0.8 Mb deleted region and *TaQ-5A* gene were labeled with arrows. NCN: normalized coverage number, DE: de-domesticated wheat accessions; DO: domesticated wheat accessions. Source data are provided as a Source Data file.

A

```
TraesCS3D02G103200    1 MAQPPPWKAMYLYVASQAREDCARVKQSVAAAAFAAVRGALASPEVLDTR  50
                        |||||.|||||.|||.:|.:.||||::|||||     |||||:|.|||||
HvBtr1                1 MAQPPQWKAMYQYVARRAHDGCARVEESVAAA-----RGALATPMVLDTR  45

TraesCS3D02G103200   51 DAEGRYTLLDSALTHIEHASDSLSGFIVNMVVAERLALHGCGAVPSEAVA  100
                        ||.||.|||.||:||:|||||.||||||::||||.|.|||||||||..||
HvBtr1               46 DAAGRCTLLHSAVTHVEHASDCLSGFIVSVVVAELLVLHGCGAVPSRPVA  95

TraesCS3D02G103200  101 RIGDL---RDDHDYEWLGLIRLEAAREHAQEALRGVEGAFTLLASVRFML  147
                        .||.|   ||||| |||.|.||||||||.|:|||||||||||||||||||
HvBtr1               96 SIGGLRRNRDDHD-EWLALSRLEAAREHGQDALRGVEGAFTLLASVRFML  144

TraesCS3D02G103200  148 HSRNPDAPGRRQAMEGQLRAAALELQPVVISVENMCALAFMATERAIRDR  197
                        .||.|||.|||||||.||.|||:|||.||.||.||.||||:||:.|||:|
HvBtr1              145 RSRTPDAAGRRQAMEEQLHAAAVELQAVVGSVANMSALAFLATQPAIRNR  194

TraesCS3D02G103200  198 IQ 199
                        ||
HvBtr1              195 IQ 196
```

B

```
TraesCS3D02G103400    1 MEQWRNTAAEASARSFTYINGTNAVVEAINGASQQYRLAAEDCRRFRPGV  50
                        ||.||.|||||||||.||||.||||||||..|.|:|.||:||||||||||
HvBtr2                1 MEVWRKTAAEASARSLTYINATNAVVEAIMAARQRYALASEDCRRFRPGV  50

TraesCS3D02G103400   51 HPLPNAGQGASAGGLIIDLAIGRIKRISRFHAVLGIVFSLCA--AHMGL-  97
                        ||.|||||||||||..::|||.||||.|||.||||.||||||  |.:||
HvBtr2               51 HPPPNAGQGASAGGDFVELAIDRIKRFSRFQAVLGNVFSLCAAPARIGLQ  100

TraesCS3D02G103400   98 -QANAPWWWDRWQLHHADPARHAETALQCLHSAKSHGHAALGVFHVMLRP  146
                         |.|||||..||||||||.|.||||||.||||||||||||||||||||||
HvBtr2              101 VQGNAPWWRHRWQLHHADAAHHAETALHCLHSAKSHGHAALGVFHVMLRP  150

TraesCS3D02G103400  147 PSPRAVAHAWAPAAEQLLRRAMDDLAMAEAAVERMRPAIVAQYSDAWMLL  196
                        |||||:||||||||||||||.|||||||||||:|||||||||:.||.|||
HvBtr2              151 PSPRALAHAWAPAAEQLLRRVMDDLAMAEAAVDRMRPAIVAQFFDASMLL  200

TraesCS3D02G103400  197 HG    198
                        ||
HvBtr2              201 HG    202
```

Supplementary Figure 31. Pairwise protein sequence alignment of barley Btr1/2 and its wheat homolog genes. (A) Pairwise protein sequence alignment of TraesCS3D02G103200 and *HvBtr1*. (B) Pairwise protein sequence alignment of TraesCS3D02G103400 and *HvBtr2*. Alignment was performed using EMBOSS Needle web server.

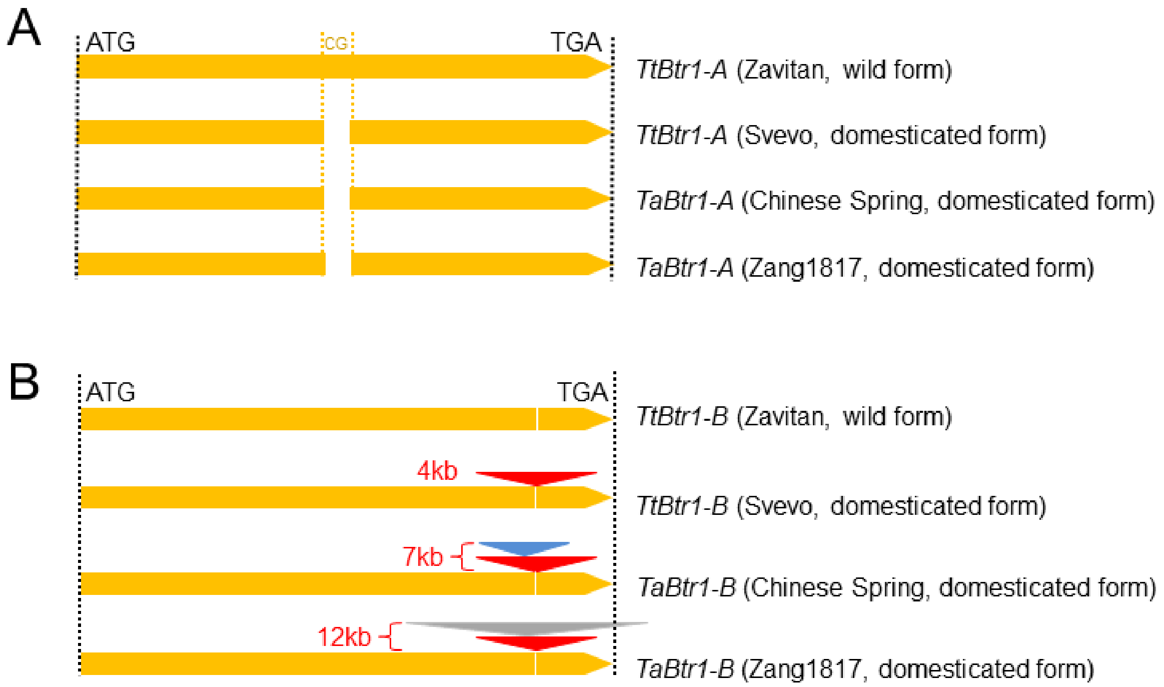

Supplementary Figure 32. Schematic representation of the wild-type Zavitan allele of *TtBtr1-A*, and its loss-of-function domesticated allele in Svevo, which carrying a CG dinucleotide deletion. Chinese Spring and Zang1817 harbors the same domesticated haplotype of *Btr1* as Svevo (A). Analogous illustration of the wild-type Zavitan allele of *TtBtr1-B* and its loss-of-function domesticated alleles in Svevo, Chinese Spring and Zang1817 assemblies. Comparing with the wild-type, the loss-of-function domesticated alleles of *Btr1* showed 4kb, 7kb and 12kb large insertions in their gene body, respectively.

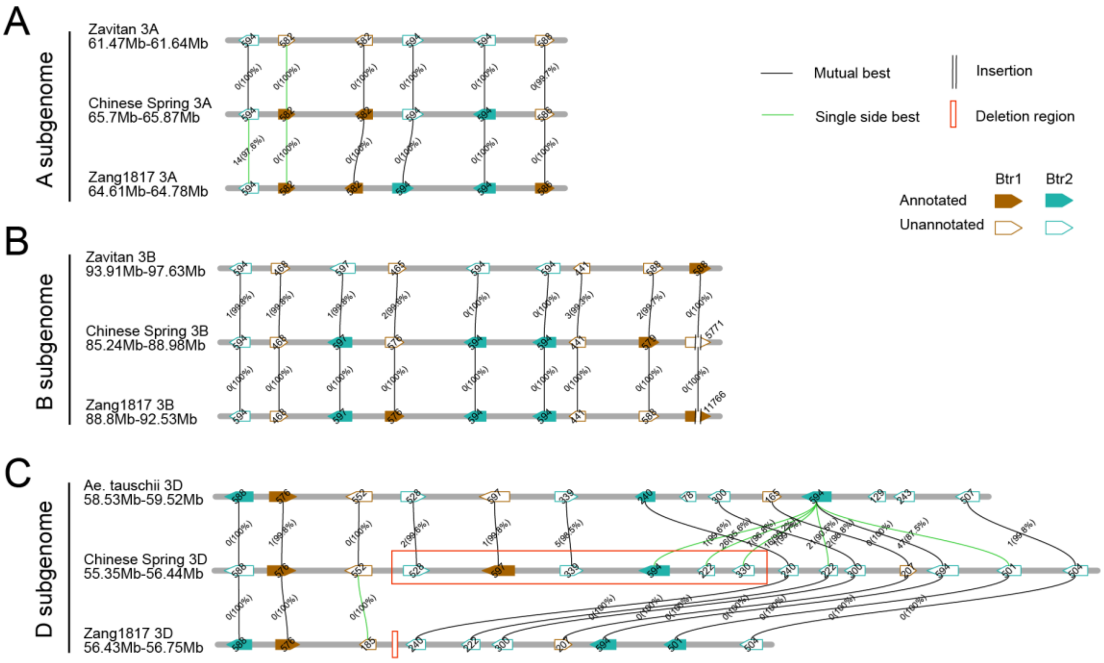

Supplementary Figure 33. A full survey of the *Btr1/2* genes along with its homologs with the collinearity analysis acrossing Zang1817, Chinese Spring and progenitor of wheat wild emmer on A subgenome (A), B subgenome (B), Aegilops tauschii on D subgenome (C). Lengths in nucleotides of gene (filled box) and pseudogene (empty box) are shown on the gene symbols. Genes (or pseudogenes) linked by black lines indicates they are best hit of each other by BLASTN analyze. Green lines indicate best hit from single direction. Number of different nucleotides for each gene pair is shown on the connection line, together with the identity in percentage (in parentheses).

Supplementary Table 1. Statistics of transposon elements for the Zang1817 assembly.

Class	Subclass	Superfamily	Percentage (%)
Class1			63.91
	LTR-retrotransposons		62.76
		Copia	15.28
		Gypsy	44.16
	Non-LTR-retrotransposons		
		Long interspersed nuclear	1.15
Class2			18.82
	DNA transposons		18.45
		CACTA	17.16
		hAT	0.01
		Tc1	0.50
		Mutator	0.46
		PIF	0.22
		MITE	0.10
	Helitron		0.01
Total TEs			82.74
Other repeats			1.57
Size of TE-related sequences (Gb)			11.62
Assembled sequence assigned to chromosomes (Gb)			14.05

Supplementary Table 2. Assessment of gene coverage rate using BUSCO.

	Whole genome	A subgenome	B subgenome	D subgenome
Complete (C)	1433 (99.5%)	1385 (96.2%)	1359 (94.4%)	1394 (96.8%)
Complete and single-copy (S)	30 (2.1%)	1338 (92.9%)	1327 (92.2%)	1368 (95.0%)
Complete and duplicated (D)	1403 (97.4%)	47 (3.3%)	32 (2.2%)	26 (1.8%)
Fragmented (F)	3 (0.2%)	27 (1.9%)	24 (1.7%)	20 (1.4%)
Missing (M)	4 (0.3%)	28 (1.9%)	57 (3.9%)	26 (1.8%)

Supplementary Table 3. Best fitting parameters for the eight models of demographic analysis on the origin of Tibetan semi-wild wheats.

Model	# epoch	Migration in epoch 1	Migration in epoch 2	Migration direction	N_1	N_2	T_1	T_2	m_1	m_2	Log likelihood
A	2	no			16172.5		1423.1				-1891.6752
B	2	yes		both	16212.0		1425.8		41.5		-1891.6775
**C**	**3**	**no**	**no**		**67092.1**	**12126.2**	**536.0**	**958.1**			**-1891.1458**
D	3	yes	no	both	230673.9	15999.0	24.1	1405.7	373.9		-1891.7657
E	3	no	yes	both	79562.0	6946.2	1070.7	499.8		3248.3	-1891.4512
F	3	yes	yes	both	82697.8	12098.7	536.0	967.5	37.5	56.3	-1891.2068
G	3	yes	no	from TL to TS	594744.1	11334.2	536.0	968.9	13.4		-1891.6673
H	3	no	yes	from TL to TS	85020.1	12217.3	536.0	978.2		2654.7	-1891.2461

The parameters by demographic units were scaled (N_e=67003 for effective population size, $2N_e$=134006 for time and migration rate) as to transform into biologically meaningful estimates. N_1: effective population size in epoch 1; N_2: effective population in epoch 2; T_1: the time during of epoch 1; T_2: the time during of epoch 2; m_1: migration in epoch 1; m_2: migration in epoch 2.
