# Supplementary material for: Origin and adaptation to high altitude of Tibetan semi-wild wheat
Source: Nat Commun. 2020 Oct 8;11:5085. doi: 10.1038/s41467-020-18738-5 (PMC7545183; doi:10.1038/s41467-020-18738-5)
Supplement: Supplementary file 2 — Description of Additional Supplementary Files [file 41467_2020_18738_MOESM2_ESM.docx]

**Description of Additional Supplementary Files**

File name: Supplementary Data 1.

Description: List of CS-specific retained genes and annotations

File name: Supplementary Data 2.

Description: List of Zang1817-specific genes and annotations.

File name: Supplementary Data 3.

Description: List and description of the 308 wheat accessions used in this study.

File name: Supplementary Data 4.

Description: List of the SNP/InDel polymorphisms leading to gain of stop codons across all wheat accessions.

File name: Supplementary Data 5.

Description: Geographical information of samples in high-altitude (HA) and lowaltitude (LA) group.
