# Supplementary material for: Origin and adaptation to high altitude of Tibetan semi-wild wheat
Source: Nat Commun. 2020 Oct 8;11:5085. doi: 10.1038/s41467-020-18738-5 (PMC7545183; doi:10.1038/s41467-020-18738-5)
Supplement: Supplementary file 8 — Reporting Summary [file 41467_2020_18738_MOESM8_ESM.pdf]

nature research

Corresponding author(s): Qixin Sun

Last updated by author(s): Aug 7, 2020

Reporting Summary

Nature Research wishes to improve the reproducibility of the work that we publish. This form provides structure for consistency and transparency in reporting. For further information on Nature Research policies, see Authors & Referees and the Editorial Policy Checklist .

Statistics

For all statistical analyses, confirm that the following items are present in the figure legend, table legend, main text, or Methods section.

n/a	Confirmed	
☐	☒	The exact sample size (*n*) for each experimental group/condition, given as a discrete number and unit of measurement
☐	☒	A statement on whether measurements were taken from distinct samples or whether the same sample was measured repeatedly
☐	☒	The statistical test(s) used AND whether they are one- or two-sided<br>*Only common tests should be described solely by name; describe more complex techniques in the Methods section.*
☐	☒	A description of all covariates tested
☐	☒	A description of any assumptions or corrections, such as tests of normality and adjustment for multiple comparisons
☐	☒	A full description of the statistical parameters including central tendency (e.g. means) or other basic estimates (e.g. regression coefficient) AND variation (e.g. standard deviation) or associated estimates of uncertainty (e.g. confidence intervals)
☒	☐	For null hypothesis testing, the test statistic (e.g. *F*, *t*, *r*) with confidence intervals, effect sizes, degrees of freedom and *P* value noted<br>*Give P values as exact values whenever suitable.*
☒	☐	For Bayesian analysis, information on the choice of priors and Markov chain Monte Carlo settings
☒	☐	For hierarchical and complex designs, identification of the appropriate level for tests and full reporting of outcomes
☒	☐	Estimates of effect sizes (e.g. Cohen's *d*, Pearson's *r*), indicating how they were calculated

Our web collection on statistics for biologists contains articles on many of the points above.

Software and code

Policy information about availability of computer code

Data collection	No software was used for data collection.
Data analysis	Softwares used in this study include the followings: Trimmomatic(v0.36), Bamtools(v2.4.1), Samtools(v1.3.1), GATK(v3.8), ADMIXTURE (v1.3.0), PLINK(v1.9), EIGENSOFT(v6.1.4), TASSEL(v5), VCFtools(v0.1.13), OrthoMCL(v2.0.9), MCScanX(http://chibba.pgml.uga.edu/mcscan2/), Quota Align(https://github.com/tanghaibao/quota-alignment), BWA-mem(v0.7.15), MUMmer(v4), BLAST(v2.6.0), bedtools (v2.27.1), BUSCO(v3), MAFFT(v7), SnpEff(v4.3), SolexaQA(v2.0), WUblast(v2.0), GeneWise(v2.4.1), Trinity(v2.4), BLAT(v36), PASA(v2.1.0), Augustus(v2.5.5), Genscan(v1.0), Geneid(v1.4), GlimmerHMM(v3.0.4), SNAP(v2006-07-28), Tophat(v2.0.13), Cufflinks(v2.2.1), EvidenceModeler(v1.1.1), InterProScan(v5), HMMER(v3.1), RepeatMasker(vopen-4.0.5).

For manuscripts utilizing custom algorithms or software that are central to the research but not yet described in published literature, software must be made available to editors/reviewers. We strongly encourage code deposition in a community repository (e.g. GitHub). See the Nature Research guidelines for submitting code & software for further information.

Data

Policy information about availability of data

All manuscripts must include a data availability statement. This statement should provide the following information, where applicable:

- Accession codes, unique identifiers, or web links for publicly available datasets
- A list of figures that have associated raw data
- A description of any restrictions on data availability

All data and genetic material used for this paper are available from the authors on request. The genome assembly and annotation of the Zang1817 genome have been deposited at the Genome Warehouse in BIG Data Center under accession number GWHAMKB00000000 [https://bigd.big.ac.cn/gwh/Assembly/8807/show]. The genome assembly and full annotation data are also available from website http://wheat.cau.edu.cn/Zang1817_genome/. The genomic sequencing reads and RNA-seq reads have been deposited under NCBI Sequence Read Archive with Project ID PRJNA596843 and PRJNA596859, respectively. The raw reads of 73 previously published re-sequenced accessions were available under NCBI Sequence Read Archive code PRJNA476679 [https://www.ncbi.nlm.nih.gov/bioproject/PRJNA476679] and from https://down- loads-qcif.bioplatforms.com/bpa/wheat_cultivars/cultivars/. The Whole Exome Capture dataset containing 1,026 hexaploid

wheat accessions is downloaded from http://wheatgenomics.plantpath.ksu.edu/1000EC/. Pfam databases used in this study were available at ftp://ftp.ebi.ac.uk/pub/databases/Pfam/releases/Pfam27.0/. KEGG databases used in this study were available at https://www.kegg.jp/kegg/download/. A reporting summary for this article is available as a Supplementary Information file. The source data underlying Figures. 1C-D, 2B, 3A-C and 4A and Supplementary Figures. 3, 5, 8-9, 11, 13-15, 20-21, 23-25 and 27-30 are provided as a Source Data file.

Field-specific reporting

Please select the one below that is the best fit for your research. If you are not sure, read the appropriate sections before making your selection.

- [x] Life sciences
- [ ] Behavioural & social sciences
- [ ] Ecological, evolutionary & environmental sciences

For a reference copy of the document with all sections, see nature.com/documents/nr-reporting-summary-flat.pdf

Life sciences study design

All studies must disclose on these points even when the disclosure is negative.

Sample size	The re-sequenced hexaploid wheat collection contains 318 accessions in total, including 245 newly sequenced accessions by authors, and 73 other accessions were downloaded from published database as detailed in the Methods.
Data exclusions	No data were excluded.
Replication	Not applicable. For genome assembly, annotation, comparision, whole genome sequencing, no replication is needed.
Randomization	Not applicable. For sequencing and analyzing, no randomizaion is needed.
Blinding	Not applicable. Sequencing and phenotype data were collected/analyzed in systematic way which involved no bias.

Reporting for specific materials, systems and methods

We require information from authors about some types of materials, experimental systems and methods used in many studies. Here, indicate whether each material, system or method listed is relevant to your study. If you are not sure if a list item applies to your research, read the appropriate section before selecting a response.

Materials & experimental systems

n/a	Involved in the study	
x		Antibodies
x		Eukaryotic cell lines
x		Palaeontology
x		Animals and other organisms
x		Human research participants
x		Clinical data

Methods

n/a	Involved in the study	
x		ChIP-seq
x		Flow cytometry
x		MRI-based neuroimaging
